# mESC: An Enhanced Escape Algorithm Fusing Multiple Strategies for Engineering Optimization

**DOI:** 10.3390/biomimetics10040232

**Published:** 2025-04-08

**Authors:** Jia Liu, Jianwei Yang, Lele Cui

**Affiliations:** 1 Faculty of Mechanical Engineering, Shaanxi University of Technology, Hanzhong 723000, China; 2 Faculty of Art and Design, Xi’an University of Technology, Xi’an 710054, China; 1230211018@stu.xaut.edu.cn; 3 Department of Applied Mathematics, Xi’an University of Technology, Xi’an 710054, China; cuilele2024@sina.com

**Keywords:** escape algorithm, multi-strategy enhanced version, meta-heuristic algorithm, realistic optimization problems

## Abstract

A multi-strategy enhanced version of the escape algorithm (mESC, for short) is proposed to address the challenges of balancing exploration and development stages and low convergence accuracy in the escape algorithm (ESC). Firstly, an adaptive perturbation factor strategy was employed to maintain population diversity. Secondly, introducing a restart mechanism to enhance the exploration capability of mESC. Finally, a dynamic centroid reverse learning strategy was designed to balance local development. In addition, in order to accelerate the global convergence speed, a boundary adjustment strategy based on the elite pool is proposed, which selects elite individuals to replace bad individuals. Comparing mESC with the latest metaheuristic algorithm and high-performance winner algorithm in the CEC2022 testing suite, numerical results confirmed that mESC outperforms other competitors. Finally, the superiority of mESC in handling problems was verified through several classic real-world optimization problems.

## 1. Introduction

### 1.1. Research Background

The optimization problem is a type of problem that optimizes the objective function that meets the constraints [1]. This type of problem involves many fields, such as physical chemistry [2], biomedical [3], economics and finance [4], logistics and operations management [5], science and technology, and machine learning [6]. Optimization problems are commonly present in the real world, such as path planning, image processing, feature selection, etc. Through optimization, specific excellent parts can be extracted to achieve the goal of improving overall performance.

### 1.2. Literature Review

The traditional methods for solving optimization problems such as the conjugate gradient method and momentum method have obvious disadvantages such as low efficiency and unsatisfactory optimization results. In this regard, metaheuristic algorithms can provide a novel and efficient approach. Metaheuristic algorithms include some classic algorithms inspired by the concept of selective elimination, such as particle swarm optimization (PSO) [7], differential evolution (DE) [8], etc. It also includes some algorithms inspired by animals, such as the zebra optimization algorithm (ZOA) proposed based on zebra foraging and predator avoidance behavior [9], the moth flame optimization (MFO) inspired by the nature of moths [10], spider wasp optimization (SWO) inspired by the survival behavior of spider bees [11], the seahorse optimization (SHO) [12] proposed based on the biological habits of seahorses in the ocean, the artificial hummingbird algorithm (AHA) [13] proposed based on the flight and foraging of hummingbirds, and the dwarf mongoose optimization algorithm (DMOA) [14] proposed based on the collective foraging behavior of dwarf mongoose. Metaheuristic algorithms also include algorithms inspired by physics and chemistry, such as the multi-verse optimization (MVO) proposed based on the concept of physical motion of celestial bodies in the universe, and the planetary optimization algorithm (POA) inspired by Newton’s law of gravity [15], algorithms proposed under the influence of human development behavior, such as the imperialist competition algorithm (ICA) inspired by weak strong cannibalism between countries [16], teaching–learning based optimization (TLBO) that simulates the process of human “teaching” and “learning” [17], in addition to sled dog optimization (SDO) [18], gray wolf optimization (GWO) [19], osprey optimization algorithm (OOA) [20], dung beetle optimization (DBO) [21], gravity search algorithm (GSA) [22], big bang big crunch (BBBC) [23], and other algorithms. In addition, there are also algorithms that have been improved on existing algorithms, such as the enhanced bottlenose dolphin optimization (namely EMBDO) for drone path planning with four constraints [24], the improved Kepler optimization algorithm (namely CGKOA) for handling engineering optimization problems [25], the superior eagle optimization algorithm (namely SEOA) for path planning [26], and the artificial rabbit optimization (namely MNEARO) for optimizing several engineering problems [27].

However, it is unrealistic to use one algorithm to solve all problems, and constantly proposing new algorithms and improving them is the most effective approach. The proposal of ESC to compound this demand was inspired by crowd evacuation behavior, and ESC [28] simulated three types of crowd behavior. ESC validates its superiority and competitiveness by comparing it with other competitors on two testing suites and several optimization problems. However, when balancing exploration and development, as well as handling high-dimensional situations, ESC may fall into local optima due to insufficient performance. Therefore, in order to better unleash the potential of ESC and further improve its performance, this article proposes mESC.

In mESC, the proposed adaptive perturbation factor strategy, boundary adjustment strategy based on the elite pool, dynamic centroid reverse learning strategy, and a proposed restart mechanism are used to enhance the overall performance of ESC. Use an adaptive perturbation factor strategy to balance population diversity during algorithm iteration. The restart mechanism enhances the exploration capability of mESC and prevents excessive convergence in the later stages of iteration. The boundary adjustment strategy based on an elite pool can screen more outstanding individuals as candidate solutions and accelerate convergence speed. Local development of dynamic centroid can reverse learning strategy balance algorithm to improve convergence accuracy and enhance local optimization.

### 1.3. Research Contribution

This study proposes a multi-strategy-based escape algorithm, mESC. This algorithm improves the original algorithm and its performance is validated through multiple experimental metrics on a test suite with 26 competitors. In addition, mESC is used for truss topology optimization and five engineering design optimization problems to affirm its superiority. The proposal of this improved algorithm provides more methods for optimizing problems, greatly improving the accuracy of optimization problems.

### 1.4. Chapter Arrangement

Section 2 first describes the concept of ESC, then presents the proposed improvement strategy for mESC, and finally presents relevant numerical experiments to verify the performance of the proposed algorithm. Section 3 confirms the practicality of the proposed algorithm through truss topology optimization and 5 engineering optimizations. Section 4 provides a summary of the entire text.

## 2. Related Work

### 2.1. Inspiration Source

The escape algorithm is proposed based on the response of the crowd during evacuation in emergency situations. This algorithm simulates various survival states and behaviors of crowds during an emergency evacuation, dividing them into three groups: calm, gathered, and panicked. A calm crowd can steadily move towards a safe zone, while a gathering crowd is in a hesitant state, and a panic crowd cannot smoothly move towards a safe zone.

### 2.2. Mathematical Modeling of ESC

(1) Initialization

Using random initialization in ESC,

(1) $$y_{i,d}=L_{d}+Rand_{i,d}\times (U_{d}-L_{d}),$$

In the formula, $L_{d},\;U_{d}$ represent the lower and upper bounds of the dth dimension, the value of the random variable $Rand_{i,d}$ follows a uniform distribution between 0 and 1.

Then, calculate the fitness values of the population and arrange them in ascending order. Store the optimal individuals in the elite pool *Epool*, where these elites are the best possible solutions that have been discovered, The specific expression is as follows:

(2) $$Epool=\left\{y_{\left(1\right)},y_{\left(2\right)},\ldots ,y_{\left(ex\right)}\right\}.$$

(2) Panic index

This value represents the panic level of the crowd during the evacuation process, expressed as follows:

(3) panic(it)=cos(πit/6Tmax),

The larger the value, the more chaotic the behavior becomes. As the iteration progresses, the panic level decreases and the crowd gradually adapts to the environment. In the equation, it represents the current iteration count and Tmax represents the maximum iteration count.

(3) Exploration stage

The three states of calmness, gathering, and panic are grouped according to the a=0.15, b=0.35, c=0.5 ratio, which is in line with the actual behavioral state of the crowd in emergency situations. Most people are in a state of panic, and only a small number of people are calm.

Calm down group update:

(4) $$y_{i,d}^{new}=y_{i,d}+m_{1}\times (\omega_{1}\times (C_{d}-y_{i,d})+Vec_{a,d})\times panic(it),$$

in the formula, $C_{d}$ represents the mean of all calm groups in the dth dimension, $Vec_{a,d}$ is obtained according to the following equation,

(5) $$Vec_{a,d}=R_{a,d}-y_{i,d}+\sigma_{d},$$

In the formula, $R_{a,d}=r_{\min,d}^{a}+r_{i,d}\times (r_{\max,d}^{a}-r_{\min,d}^{a})$ represents the group position of the calm group, $r_{\max,d}^{a},\;r_{\min,d}^{a}$ represents the maximum and minimum values of the calm group in the dth dimension, respectively, $\sigma_{d}=zd/50$ represents the individual’s adjustment value that satisfies zd∈N(0,1), $m_{1}$ is a random value of 0 or 1, and $\omega_{1}$ is the adaptive Levy weight.

Aggregation group update:

(6) $$\begin{array}{l} y_{i,d}^{new}=y_{i,d}+m_{1}\times (\omega_{1}\times (C_{d}-y_{i,d})+m_{2}\times \omega_{2}\times (y_{b,d}-y_{i,d})\\ \;\;\;\;\;\;\;\;\;\;\;\;\;\;\;\;\;\;\;\;\;\;\;\;\;\;\;\;\;\;\;\;\;\;\;\;\;+Vec_{b,d}\times panic(it)) \end{array},$$

In the equation, $y_{b,d}$ is a random individual within the panic group, and the vector $Vec_{b,d}$ is obtained according to the following equation,

(7) $$Vec_{b,d}=R_{b,d}-y_{i,d}+\sigma_{d},$$

In the formula, $R_{b,d}=r_{\min,d}^{b}+r_{i,d}\times (r_{\max,d}^{b}-r_{\min,d}^{b})$ is a random position, $r_{\max,d}^{b},\;r_{\min,d}^{b}$ is the maximum and minimum values of the cooling group, and $m_{2},\omega_{2}$ is similar to $m_{1},\omega_{1}$.

Panic Group Update: 

The impact of random indications on the fertilization pool of individuals in the panic group and other individuals,

(8) $$\begin{array}{l} y_{i,d}^{new}=y_{i,d}+m_{1}\times (\omega_{1}\times (Epool_{d}-y_{i,d})+m_{2}\times \omega_{2}\times (y_{rand,d}-y_{i,d})\\ \;\;\;\;\;\;\;\;\;\;\;\;\;\;\;\;\;\;\;\;\;\;\;\;\;\;\;\;\;\;\;\;\;\;\;\;\;\;\;\;\;\;\;\;+Vec_{c,d}\times panic(it)) \end{array},$$

In the formula, $Epool_{d}$ represents individuals in the elite pool, $y_{rand,d}$ represents randomly selected individuals in the population, and the vector $Vec_{c,d}$ is obtained according to the following equation,

(9) $$Vec_{c,d}=R_{c,d}-y_{i,d}+\sigma_{d},$$

In the formula, $R_{c,d}=r_{\min,d}^{c}+r_{i,d}\times (r_{\max,d}^{c}-r_{\min,d}^{c})$ is the group position of the panic group, and $r_{\max,d}^{c},\;r_{\min,d}^{c}$ is the upper and lower bounds of the panic group.

(4) Development stage

At this stage, all individuals remain calm and improve their position by approaching members of the elite pool. This process simulates the crowd gradually gathering towards the determined optimal exit,

(10) $$y_{i,d}^{new}=y_{i,d}+m_{1}\times \omega_{1}\times (Epool_{d}-y_{i,d})+m_{2}\times \omega_{2}\times (y_{rand,d}-y_{i,d}),$$

In the formula, $y_{i,d}$ represents the individual’s position, and $Epool_{d}$ represents the currently obtained best solution or exit.

### 2.3. The Proposed mESC Algorithm

ESC performs well in solving simple problems in low dimensions, but its performance significantly decreases when solving complex multimodal and combinatorial functions. This means that it is more prone to getting stuck in local solution spaces, and the continuous reduction in population diversity makes it difficult for the algorithm to maintain synchronous exploration and development, ultimately leading to overall performance degradation. Therefore, this article proposes some improvement strategies and develops the mESC algorithm to address these shortcomings. The specific improvement strategies are as follows.

#### 2.3.1. Adaptive Perturbation Factor Strategy

Generally speaking, the population diversity of algorithms will decrease as the algorithm runs, and the significant reduction in diversity in the later stages of iteration is not conducive to the full exploration of the population. This article proposes an adaptive perturbation factor to overcome this drawback, which adjusts the perturbation probability of individuals in the population as the iteration progresses; furthermore, it enriches the diversity of the population. The specific expression is as follows:

(11) $$ADF=0.5+0.2\times RAND\times {\left(1-\frac{it}{T\max}\right)}^{\left(2\times \frac{it}{T\max}\right)}.$$

RAND is a random number in the equation.

As ADF increases, it enhances the global search capability and avoids falling into local optima. However, as ADF gradually decreases, it improves the local search accuracy and accelerates convergence. For continuous functions, when an individual has not yet found the optimal solution, there are better solutions nearby. The adaptive perturbation factor can adjust the individual extremum, global extremum, and position term to increase the possibility of finding the global optimal solution.

#### 2.3.2. Restart Mechanism

ESC has the drawbacks of premature stagnation and getting stuck in local space, and this article uses a restart mechanism [29] to improve this. This mechanism can enhance the robustness of ESC while improving the global search capability, and effectively eliminating some poor individuals, thereby achieving the goal of enhancing algorithm convergence.

We set the worst individual factor in this article to 0.05, select the worst quantity as 0.1 Num, and Num as the population size. The position update equation for these worst individuals is as follows:

(12) $$y_{i}=Gaussian\;(Y_{best},\tau )+(RAND\times y_{i}-RAND\times Y_{best}).$$

τ satisfies the formula in the equation,

(13) $$\tau =\left|\frac{\log(it+1)}{it+1}\times (y_{i}-Y_{best})\right|,$$

In the formula, $Gaussian\;(Y_{best},\tau )$ follows a Gaussian distribution, $Y_{best}$ represents the optimal individual, and RAND is a random number.

In this strategy, the restart mechanism gives the algorithm the opportunity to jump out of local optima and explore other solution spaces, thereby increasing the probability of finding the global optimum. In addition, as the iteration progresses, the convergence speed may slow down. However, the restart mechanism is equivalent to using the fast convergence characteristics of the population in the initial stage of the iteration, and this mechanism can prompt the algorithm to continue optimization when facing local stagnation.

#### 2.3.3. Boundary Adjustment Strategy Based on Elite Pool

According to Equation (10), we have obtained the position of the entire population. Next, we will perform boundary processing on the obtained positions to ensure the correctness and robustness of the algorithm by applying specific processing methods to the boundaries or extreme cases. Generally, when an individual exceeds the population boundary, they are indiscriminately assigned a critical value to the boundary, which can lead to local aggregation of individuals on the boundary and affect the search for the global optimal solution. This article introduces the boundary adjustment strategy of the elite pool, which updates the boundaries of individuals in the population based on the selected members in the elite pool.

(14) $$y_{i,d}^{new}=\left\{\begin{array}{l} (Epool_{d}+y_{i,d})/2,y_{i,d}(it+1)>U\;or\;y_{i,d}(it+1)<L\\ y_{i,d}\;\;\;\;\;\;\;\;\;\;\;\;\;\;\;\;\;\;\;\;\;\;\;\;else \end{array}\right..$$

Therefore, the position formula of the population is updated to,

(15) $$y_{i,d}^{new}=(y_{i,d}^{new}\times \neg \;(FlagU+FlagL))+U\times FlagU+L\times FlagL,$$

In the formula, $FlagU=y_{i,d}^{new}>U,FlagL=y_{i,d}^{new}<L$.

In this strategy, the elite pool ensures that the optimal solutions of each iteration are not eliminated by storing them, and these elite solutions reduce invalid searches by concentrating on the search potential area. By retaining diverse elite solutions, it is possible to prevent the population from prematurely falling into local solutions and enhance global search capabilities. Because elite solutions come from different regions of the population, this can maintain a balance between exploration and development for the algorithm. After mastering the position of the elite solution, the boundary can be adjusted to search in the area that is most likely to find the global optimal solution.

#### 2.3.4. Dynamic Centroid Reverse Learning Strategy

This article proposes a random centroid reverse learning strategy to improve ESC. Based on the idea of reverse learning, while considering existing solutions, opposite solutions are also taken into account. By comparing with the reverse solution, choose the better solution to guide other individuals in the population to seek optimization. While balancing the concepts of adversarial learning and centroids, the robustness of mESC is improved by introducing randomness elements, in the following specific form: 

Generate integer B∈[2, Num], which is the number of randomly selected populations. Then, randomly select B individuals from the current population and calculate their centroids, expressed as follows:

(16) $$M=\frac{\sum_{a=1}^{B}V_{a}}{B}.$$

Then, generate the population’s ortho solutions about the centroid, as shown below:

(17) $$V_{a}^{*}=2\times M-V_{a}.\;a=1,\;2,\;\cdots ,\;Num.$$

Finally, using the greedy rule, the top Num individuals with the best fitness values were selected from the original improved population and the population $V_{a}\cup V_{a}^{*}$ that underwent random centroid reverse learning as the new generation population. The random centroid reverse learning strategy utilizes the excellent solutions obtained from the previous generation to guide population initialization, improve algorithm accuracy, accelerate convergence speed, and enable faster search near potentially optimal positions.

In this strategy, the centroid will dynamically update with changes in the population to ensure that the algorithm can respond to changes in the population in a timely manner to avoid premature convergence. After integrating reverse learning, the reverse solution expands the search space and increases the diversity of the population, which helps to escape from local optima

#### 2.3.5. Specific Steps of mESC

**Figure 1:**
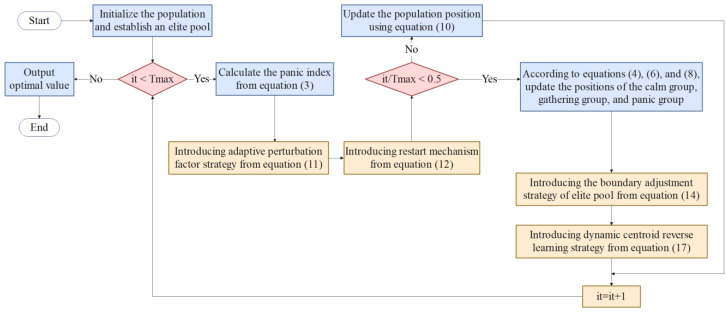
Proposed mESC flowchart.

The pseudocode of the improved escape algorithm incorporating adaptive perturbation factor, restart mechanism, boundary adjustment strategy based on elite pool, and dynamic centroid reverse learning strategy, is listed in Algorithm 1. Figure 1 shows the flowchart of mESC.
**Algorithm 1** The proposed mESCInput: Dimension D, Population $y_{i}(i=1,2,\cdots ,Num)$, Population size Num, Maximum number of iterations $T_{\max}$, The worst individual ratio Pworst is 0.1Output: Optimal fitness value $fit_{best}$
1:  Randomly initialize the individual positions of the population using Equation (1)2:  Sort the fitness values of the population in ascending order and record the current optimal individual3:  Store the refined individuals in the elite pool of Equation (2)4:  while $\left(it<T_{\max}\right)$ do5:      Calculate the panic index from Equation (3)6:      $ADF=0.5+0.2\times RAND\times {\left(1-\frac{it}{T\max}\right)}^{\left(2\times \frac{it}{T\max}\right)}.$
7:      for i=1:Num do8:          for j=1:D do9:              $\tau =\left|\frac{\log(it+1)}{it+1}\times (y_{i}-Y_{best})\right|,$
10:             $y_{i}=Gaussian\;(Y_{best},\tau )+(RAND\times y_{i}-RAND\times Y_{best}).$
11:         end for12:      end for13:      if $it/T_{\max}\leq 0.5$ then14:          Divide the population into three groups and update the three groups according to Equations (4), (6) and (8)15:      else 16:          Equation (10) updates the population17:      end18:      for i=1:Num do19:          $y_{i,d}^{new}=\left\{\begin{array}{l} (Epool_{d}+y_{i,d})/2,y_{i,d}(it+1)>U\;or\;y_{i,d}(it+1)<L,\\ y_{i,d},\;\;\;\;\;\;\;\;\;\;\;\;\;\;\;\;\;\;\;\;\;\;\;else. \end{array}\right.$
20:          $y_{i,d}^{new}=(y_{i,d}^{new}\times \neg \;(FlagU+FlagL))+U\times FlagU+L\times FlagL.$
21:      end22:      $V_{a}^{*}=2\times M-V_{a}.\;a=1,\;2,\;\cdots ,\;Num$
23:  Calculate individual fitness values and update the elite pool based on the optimal value24:  it=it+1
25:  end while26:  Return the optimal solution from the elite pool

### 2.4. Complexity Analysis of mESC

The time complexity of mESC consists of three parts: initialization, main iteration process, and dynamic centroid reverse learning: population size of N, number of iterations of $T_{\max}$, dimension of D, restart times of R, and elite pool size of K. The time complexity of mESC is as follows:

$$\begin{array}{l} O(mESC)=O(initialization)+T_{\max}(O(iterativeprocess)\\ \;\;\;\;\;\;\;\;\;\;\;\;\;\;\;\;\;\;\;\;\;+O(elite\;Pool)+O(restart\;mechanism))\\ \;\;\;\;\;\;\;\;\;\;\;\;\;\;\;\;\;=O(ND+T_{\max}((ND+N\log N)+(ND+KD)\\ \;\;\;\;\;\;\;\;\;\;\;\;\;\;\;\;\;\;\;\;\;+(ND+RND))\\ \;\;\;\;\;\;\;\;\;\;\;\;\;\;\;\;\;=O(T_{\max}N(\log N+k+R+D)) \end{array}.$$

The spatial complexity of an algorithm refers to the trend of the required storage space during its runtime. The time complexity of mESC consists of population storage, fitness value storage, and elite pool storage. However, the spatial complexity corresponding to other strategies is O(ND). The time complexity of mESC is as follows:

$$\begin{array}{l} O(mESC)=O(population\;storage)+O(fitness\;value\;storage)\\ \;\;\;\;\;\;\;\;\;\;\;\;\;\;\;\;\;\;\;\;\;\;\;\;\;\;\;\;\;\;\;\;\;\;\;\;\;\;\;+O(elite\;Pool\;storage)\\ \;\;\;\;\;\;\;\;\;\;\;\;\;\;\;\;\;=O(ND+ND+(ND+KD))\\ \;\;\;\;\;\;\;\;\;\;\;\;\;\;\;\;\;=O(ND+KD) \end{array}.$$

### 2.5. Experimental Setup

We conducted two sets of comparative experiments to validate the performance of the proposed algorithm. The first set compared mESC with 16 other newly proposed metaheuristic algorithms, including some competitive algorithms proposed in 2023 and 2024, which showed excellent performance in solving some problems. The second group conducted comparative experiments between mESC and 11 high-performance, winner algorithms, including 3 winner algorithms from CEC competitions, 4 variant algorithms from DE, and 3 variant algorithms from PSO. These algorithms have generally strong performance and are often used as competitors in comparative experiments. By comparing with current new algorithms and powerful algorithms in the past, the superiority of mESC is highlighted.

This article will include some experimental tables and images in the Supplementary Materials. Among them, Tables S1 and S2 and Tables S3 and S4, respectively, show the results of two types of parameters in the 10 and 20 dimensions. Tables S5 and S6 and Tables S7 and S8 show the experimental results and Wilcoxon results of mESC and the novel metaheuristic algorithm in 10 and 20 dimensions, respectively. Tables S9 and S14 show the running times of mESC, the new metaheuristic algorithm, and the high-performance, winner algorithm, respectively. Tables S10 and S11 and Tables S12 and S13, respectively, show the experimental results and Wilcoxon results of mESC and high-performance, winner algorithm in 10 and 20 dimensions. Figures S1 and S2 show the convergence curves and boxplots of mESC and the novel metaheuristic algorithm, respectively. Figures S3 and S4 show the convergence curves and boxplots of mESC and high-performance, winner’s algorithm, respectively.

#### 2.5.1. Benchmark Test Function

Use the CEC2022 (D = 10, 20) test kit to evaluate the performance of mESC for two comparative experiments. Set the population size of mESC to 30, with a maximum iteration of 500, and run it independently 30 times as a whole.

#### 2.5.2. Parameter Settings

The new metaheuristic algorithms compared in the first set of experiments include Parrot Optimization (PO) [30], Geometric Mean Optimization (GMO) [31], Fata Morgana Algorithm (FATA) [32], Moss Growth Optimization (MGO) [33], Crown Pig Optimization (CPO) [34], Polar Lights Optimization (PLO) [35], Newton–Raphson-based Optimizer (NRBO) [36], Information Acquisition Optimization (IAO) [37], Love Evolutionary Algorithm (LEA) [38], Escape Algorithm (ESC), Improved Artificial Rabbit Optimization (MNEARO), Artificial Hummingbird Algorithm (AHA), Dwarf Mongoose Optimization Algorithm (DMOA), Zebra Optimization Algorithm (ZOA), and Seahorse Optimization (SHO). The high performance compared in the second set of experiments, the winning algorithm includes Autonomous Particle Groups for Particle Dwarm Optimization (AGPSO) [39], Integrating Particle Swarm Optimization and Gravity Search Algorithm (CPSOGSA) [40], Improved Particle Swarm Optimization (TACPSO) [41], Bernstein–Levy Differential Evolution (BDE) [42], Bezier Search Differential Evolution (BeSD) [43], Multi Population Differential Evolution (MDE) [44], Improving Differential Evolution through Bayesian Hyperparameter Optimization (MadDE) [45], Improved LSHADE Algorithm (LSHADE-cnEpSin) [46], Improvement of L-SHADE Using Semi-parametric Adaptive Method (LSHADE-SPACMA) [47], and Improving SHADE with Linear Population Size Reduction (LSHADE) [48]. All parameters are given in Table 1.

#### 2.5.3. Empirical Test

Before starting this section, we defined mESC1, mESC2, mESC3, and mESC4 as algorithms that separately introduce adaptive perturbation factors, restart mechanisms, boundary adjustment strategies based on elite pools, and dynamic centroid reverse learning strategies for mESC. Subsequently, we conducted impact analysis on mESC, ESC, mESC1, mESC2, mESC3, and mESC4, and discussed the degree of impact of the proposed individual strategies on the original ESC and mESC.

**Figure 2:**
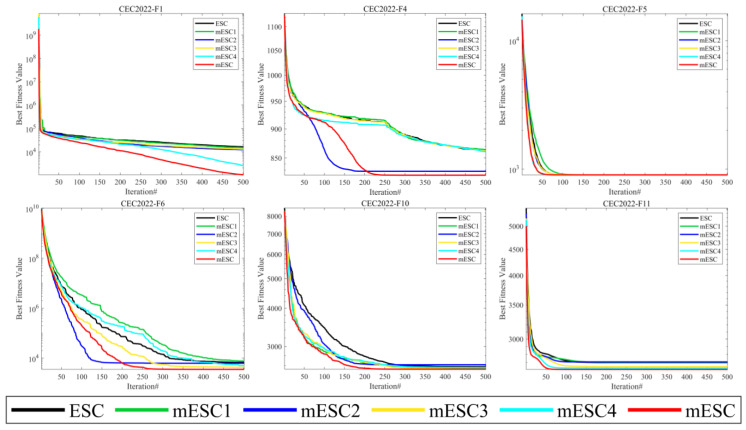
Convergence curves of mESC, ESC, mESC1, mESC2, mESC3, and mESC4 on CEC2022.

Given the convergence of six algorithms from Figure 2, when solving the F1 problem, mESC and mESC4 have the best convergence effect, while other competitors have all fallen into local optima after a mid-term operation. However, mESC has a convergence effect that is 1 or 2 orders of magnitude better than mESC4. For F4, mESC and mESC2 have the closest convergence effects. For F5, visually speaking, the convergence of the six algorithms is very close, but mESC has the fastest convergence speed. For F6, mESC has the highest convergence accuracy, while mESC2 converges faster than mESC. For F10, mESC1, mESC3, mESC4, and mESC, their convergence is very close at the beginning of the operation, but mESC has a higher convergence accuracy. The convergence of F11, mESC, and mESC4 are very similar, while the convergence accuracy of other algorithms is inferior to mESC. The four strategies play different roles in improving the algorithm, which is related to their different mechanisms, and combining them will produce better results.

Table 2 presents the 12 experimental results on the CEC2022 test suite, and according to this table, mESC has the best overall performance. MESC has the optimal mean on 10 questions and the optimal standard deviation on four questions, indicating that the combination of these four improvement strategies is effective. MESC is optimal on unimodal functions and can also achieve optimality on fundamental functions. The performance of mESC3 is optimal on the mixed function F6, while mESC reaches its optimum on F7 and F8. MESC1 performs the best on F10, outperforming mESC, while mESC performs better on other functions. Overall, we can demonstrate that mESC performs the best and validates the effectiveness of the four strategies.

#### 2.5.4. Sensitivity of the Parameters

In the proposed mESC, the main strategies introduced are the proportion of worst-performing individuals α in the restart mechanism and the parameter adaptive disturbance factor adjustment range β in the adaptive disturbance factor strategy, which affect the performance of the algorithm. In this section, we determine the relevant parameters on the CEC2022 test set. The variable dimensions in the experiment are the standard dimensions 10 and 20 of the test set, with a maximum iteration of 500. The average values obtained from different parameters are shown in Tables S1–S4 and the ranking is given in the last row of the table.

The worst individual ratio α determines the proportion of individuals replaced after each restart. A smaller value will make it easier to fall into local optima while accelerating convergence, while a larger value will significantly slow down convergence while making global search stronger. The parameter ranges from 0.05 to 0.20 and increases by 0.05. As shown in Tables S1 and S2, α=0.05 achieves better performance and performance in both 10 and 20 dimensions based on the final ranking.

The adaptive interference factor adjustment range β determines the variation range of the interference factor. When the problem to be solved requires a high convergence speed, this range can be appropriately reduced. However, for scenarios with high solution quality, the variation range should be increased. In Tables S3 and S4, values are selected between 0.9 and 0.5 in increments gradually decreasing by 0.01. The results indicate that the most promising range is when the parameter decreases between 0.7 and 0.5.

### 2.6. Experimental Analysis of mESC and New Metaheuristic Algorithm

As shown in Figure S1, mESC has good convergence performance compared to other competitors when dealing with different dimensional problems on CEC2022. Given MESC in F1 on the 10 dimensions, F3, F5, F7, the convergence speed in the early stage is the fastest, and it can quickly find the global optimum. ZOA has the fastest convergence speed in the early stage on F4, while LEA and CPO fall into local optima prematurely on multiple functions. The optimization accuracy of mESC is much better than other algorithms. On the 20-dimensional F4 problem, ZOA, SHO, and NRBO converge slightly faster than the proposed algorithm in the early stages of iteration, but mESC has the best convergence accuracy. On F3, F4, and F11, AHA, MNEARO, ESC, and mESC have very similar convergence accuracy in the middle and later stages of iteration, but mESC has higher convergence accuracy.

From Tables S5 and S6, it can be seen that mESC’s overall average ranking and ranking on various benchmark functions are superior to other competitors, demonstrating superior performance. The overall ranking in 10 dimensions is 1.83, ranking first on 5 benchmark functions. MNEARO’s overall ranking is second only to mESC. The overall ranking in 20 dimensions is 1.50, ranking first on 8 benchmark functions, while MGO ranks worst in two dimensions.

Tables S7 and S8 present the statistical results of the Wilcoxon rank sum test on different dimensions of CEC2022. On the 10 dimensions, mESC outperforms PO, FATA, MGO, ECO, CPO, PLO, NRBO, LEA, SHO, ZOA, and MNEARO on all 12 benchmark functions. On the 20 dimensions, mESC outperforms PO, FATA, MGO, NRBO, ECO, CPO, PLO, LEA, SHO, ZOA, and MNEARO on 12 benchmark functions, and also outperforms mESC on five functions.

Figure S2 shows the boxplots of mESC and other competitors in different dimensions of CEC2022. Overall, mESC has shorter boxes on different functions, especially F1, F3, F5, and F7 on the 10 dimensions and F2 on the 20 dimensions, F9, F10, and F11, This demonstrates the superiority and stability of mESC.

The running time of an algorithm is also an indicator for evaluating its performance. As shown in Table S9, CPO has the shortest running time and ranks first, while mESC ranks very low in both dimensions of running time, which is in line with our expectations. Because it is inevitable that the algorithm will run for too long while ensuring its performance, we allow this phenomenon to occur.

### 2.7. Experimental Analysis of mESC and High-Performance, Winner Algorithm

The experimental results of mESC and high-performance, winner algorithm on CEC2022 are presented in Tables S10 and S11. On the 10 dimensions, mESC outperforms other competitors on 8 benchmark functions, ranking first overall with an average ranking of 1.58. MadDE, ranked second overall, outperforms the proposed algorithm on two benchmark functions with an average ranking of 3.50. MDE performs better than mESC on two benchmark functions, but its overall ranking is only fourth. Overall, mESC’s testing with these competitors has validated its superior performance.

Tables S12 and S13 show the Wilcoxon rank sum test of mESC and high-performance, winner algorithm on the CEC2022 test suite (symbol ♢ = 3.0199 × 10^−11^ in the table). On the 10 dimensions, BDE, BeSD, MDE, and MadDE have better *p*-values than mESC on 2, 2, 2, and 3 benchmark functions, respectively. The *p*-values of mESC on 12 functions are completely superior to AGPSO, TACPSO, LSAHDE cnEpSin, and LSHADE. On the 20 dimensions, mESC outperforms the other seven competitors on 12 benchmark functions and only has approximate performance on one function compared to CPSOGSA, TACPSO, and MDE. Therefore, we can say that the proposed strategy significantly improves the algorithm.

Figure S3 shows the convergence curve of mESC and high-performance, winner algorithm on CEC2022. In terms of 10 dimensions, the convergence of mESC is superior to other competitors. LSAHDE-SPACMA, LSAHDE, and CPSOGSA began to fall into local optima in the mid-iteration of F1 and F4. On the 20th dimension, as the dimension increases, the convergence speed of mESC improves. The accuracy of mESC on F1 and F4 is significantly better than other competitors, and some algorithms fall into local optima early on F3, F7, and F11 while mESC can maintain stability for optimization.

Table S14 presents the comparison results of the running time between mESC and high-performance, winner algorithms. The running time ranking of mESC is 12th in both dimensions, which is in line with our initial expectations. LSHADE has the shortest running time and ranks first.

Figure S4 shows the boxplots of mESC and other algorithms in different dimensions of CEC2022. Overall, mESC has shorter boxes for the vast majority of functions, especially F1, F5, F7, F1 in the 10 dimensions, and F1 in the 20 dimensions, F2, F4, F8. This indicates the stability of mESC.

## 3. Real World Application Solving

Next, we apply the proposed mESC to five engineering optimizations and two truss topology optimizations, highlighting its superiority through the results of solving these problems with mESC and other competitors.

### 3.1. mESC Optimization of Truss Topology Design Problem

Structural optimization design refers to the design of a scheme with the goals of minimizing volume, minimizing cost, and maximizing stiffness under given constraints. Meanwhile, truss optimization is aimed at reducing weight as much as possible to achieve resource recycling efficiency. The specific constraints are as follows:

Find

(18) $$x=\left\{A_{1},A_{2},\ldots ,A_{n}\right\}.$$

Minimize

(19) $$f(x)=\sum_{i=1}^{n}B_{i}A_{i}\rho_{i}L_{i}+\sum_{j=1}^{m}b_{j},{\;B}_{i}=\left\{\begin{array}{l} 0,{\;\mathrm{if}\;A}_{i}<\;\mathrm{critical}\;\mathrm{area}\\ 1,{\;\mathrm{if}\;A}_{i}\geq \;\mathrm{critical}\;\mathrm{area} \end{array}\right..$$

$A_{i}$ is the cross-sectional area, $b_{j}$ is the mass value, $\rho_{i}$ is the mass density, and $L_{i}$ is the length of the rod.

Limitation

(20) $$\left\{\begin{array}{l} g_{1}(x):\left|B_{i}\sigma_{i}\right|-\sigma_{i}\leq 0,\;g_{2}(x):\left|\delta_{i}\right|-\delta_{j}^{\max}\leq 0,\;g_{3}(x):fr-f_{r}^{\min}\geq 0\\ g_{4}(x):\left|B_{i}\sigma_{i}^{comp}\right|-\sigma_{i}^{cr}\leq 0,\;\sigma_{i}^{cr}=\frac{k_{i}A_{i}E_{i}}{L_{i}^{2}},\;g_{5}(x):A_{r}^{\min}\leq A\leq A_{r}^{\max}\;\\ g_{6}:check\;on\;validity\;of\;structure,\;g_{7}:check\;on\;kinematic\;stability \end{array}\right.,$$

In the formula, i=1,2,…,n, j=1,2,…,m, $\sigma_{i}$ represents stress, $g_{1}(x)$ represents stress constraint, and $B_{i}$ represents binary bits, $g_{2}(x)$, $g_{3}(x)$ and $g_{4}(x)$ are displacement constraints, natural frequency constraints, and Euler buckling constraints, respectively; $g_{5}(x)$ is a cross-sectional area constraint, and without truss connections, this truss topology is invalid, i.e., $g_{6}$. The consideration of motion stability will ensure the smooth use of the truss, i.e., $g_{7}$.

In the truss topology optimization, if the solved cross-sectional area is less than the critical area, it is assumed that the member is removed from the Truss; otherwise, the member is retained. If the loading node, the supporting node, and the non-erasable node are not connected through any Truss pole, the generated Truss topology is invalid (g6). In order to ensure the generation of a motion stable Truss structure, motion stability (g7) is included in the design constraints, which are mainly divided into the following two criteria: 

(1) The degrees of freedom of the structure calculated using the Grubler criterion should be less than 1;

(2) The motion stability of the structure is checked by the positive definiteness of the stiffness matrix created by component connections, and the global stiffness matrix should be positive definite.

To evaluate whether the design scheme meets the constraints, a penalty letter [49] needs to be introduced, as follows: 

Punishment

(21) $$f(x)=\left\{\begin{array}{l} 10^{9}{\;\;\;\;\;\;\;\;\;\;\;\;\;\;\;\;\;\;\;\;\;\;\mathrm{if}\;g}_{7}\;\mathrm{is}\;\mathrm{violated}\\ 10^{8}{\;\;\;\;\;\;\;\;\;\;\;\;\;\;\;\;\;\;\;\;\;\;\mathrm{if}\;g}_{6}\;\mathrm{is}\;\mathrm{violated}\;\mathrm{with}\;\mathrm{degree}\;\mathrm{of}\;\mathrm{freedom}\\ 10^{7}{\;\;\;\;\;\;\;\;\;\;\;\;\;\;\;\;\;\;\;\;\;\;\mathrm{if}\;g}_{6}\;\mathrm{is}\;\mathrm{violated}\;\mathrm{with}\;\mathrm{positive}\;\mathrm{definiteness}\\ f(x)\cdot F_{penalty}\;\;\;\;\;\;\;\mathrm{otherwise} \end{array}\right.,$$

In the formula, $F_{penalty}={\left(1+\alpha \cdot C\right)}^{\beta },\;C=\sum_{i=1}^{q}C_{i},{\;C}_{i}=\left|1-p_{i}/p_{i}^{*}\right|$, $p_{i}$ represent the level of constraint violation, q represents the activity constraint, and α and β are 2. The Euler buckling coefficient is set to 4.0 kg and the mass is set to 5.0 kg.

#### 3.1.1. Optimization of 24 Bar 2D Truss

**Figure 3:**
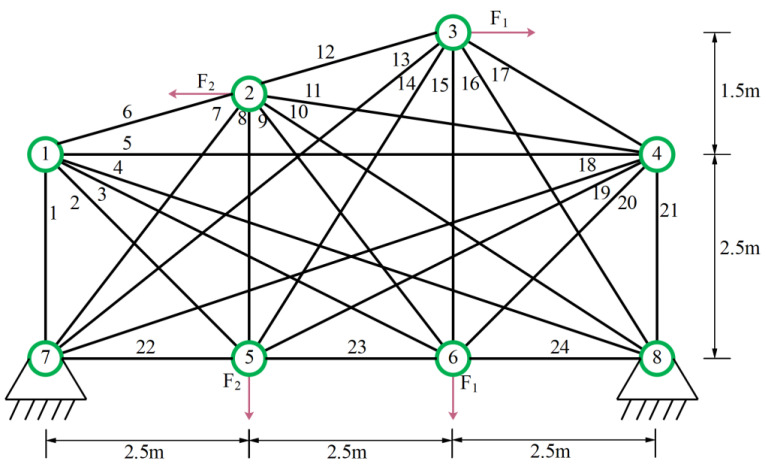
24 Pole truss structure.

In the experiment, we selected nine algorithms including NRBO, MPSO [50], CPO, MFO, BWO, WOA, HHO [51], BSD [52], and TSA [53] as competitors. The relevant structure is shown in Figure 3, and the design variables are the segmental members of the truss. The parameters required for the experiment are described in Table 1. The experimental results of all algorithms after 20 runs are shown in Table 3, where the optimal weights and the optimal values of the average are marked with black bold graphs to evaluate the superiority and inferiority of the algorithm.

**Figure 4:**
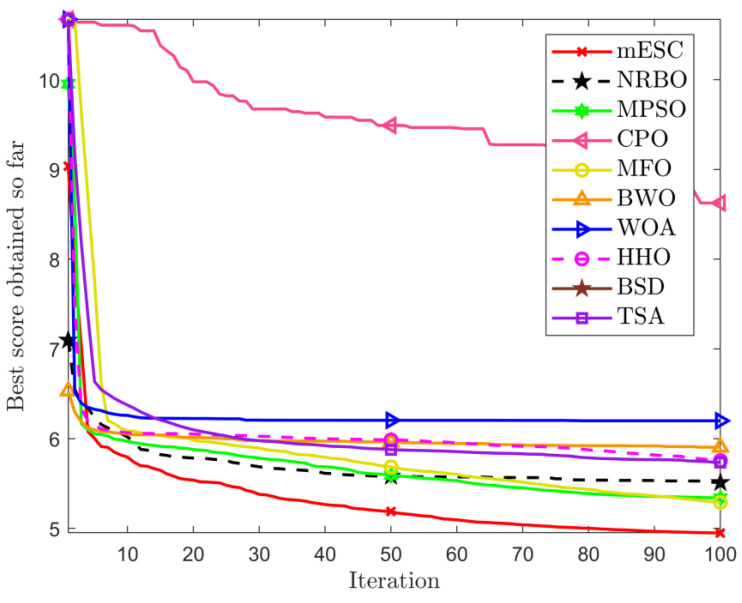
Comparison of convergence of various algorithms on 24 bars.

**Figure 5:**
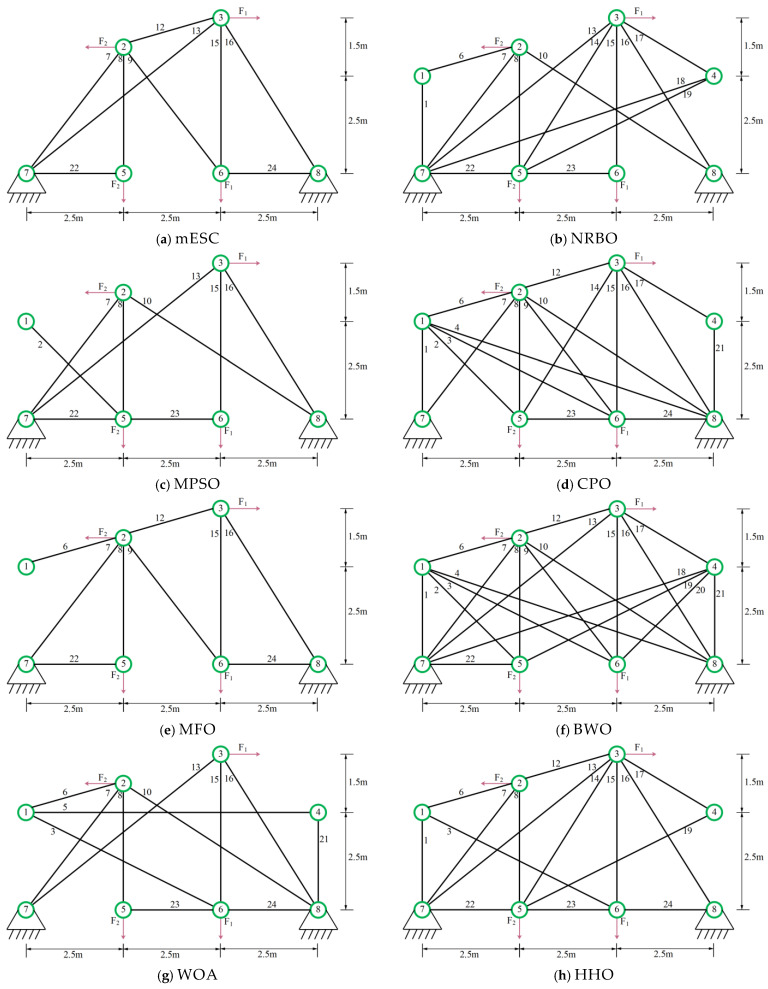

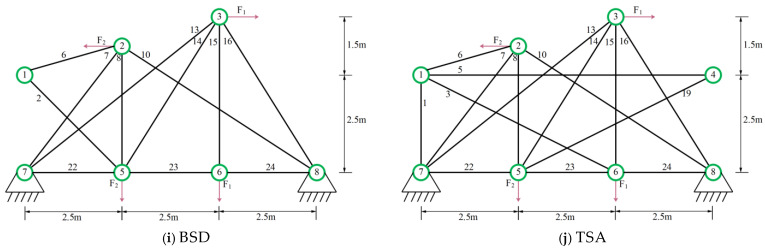
Topology optimization of 24 bar truss using various algorithms.

The data in Table 4 proves that the optimal weight and overall average value found by mESC are generally the best, with a minimum weight of 121.5840 and an overall average value of 140.5701. This indicates that mESC’s performance in solving this problem is the most stable compared to other competitors. Figure 4 shows the convergence graph of optimizing the problem among competitors. CPO begins to fall into local optima in the early and middle stages of iteration; MESC performed even better. Overall, the comprehensive performance of mESC has been validated in solving this optimization problem. Figure 5 shows the truss diagrams of each algorithm after removing the rods based on experimental results, with mESC having the highest number of rods removed.

#### 3.1.2. Optimization of 72 Bar 3D Truss

**Figure 6:**
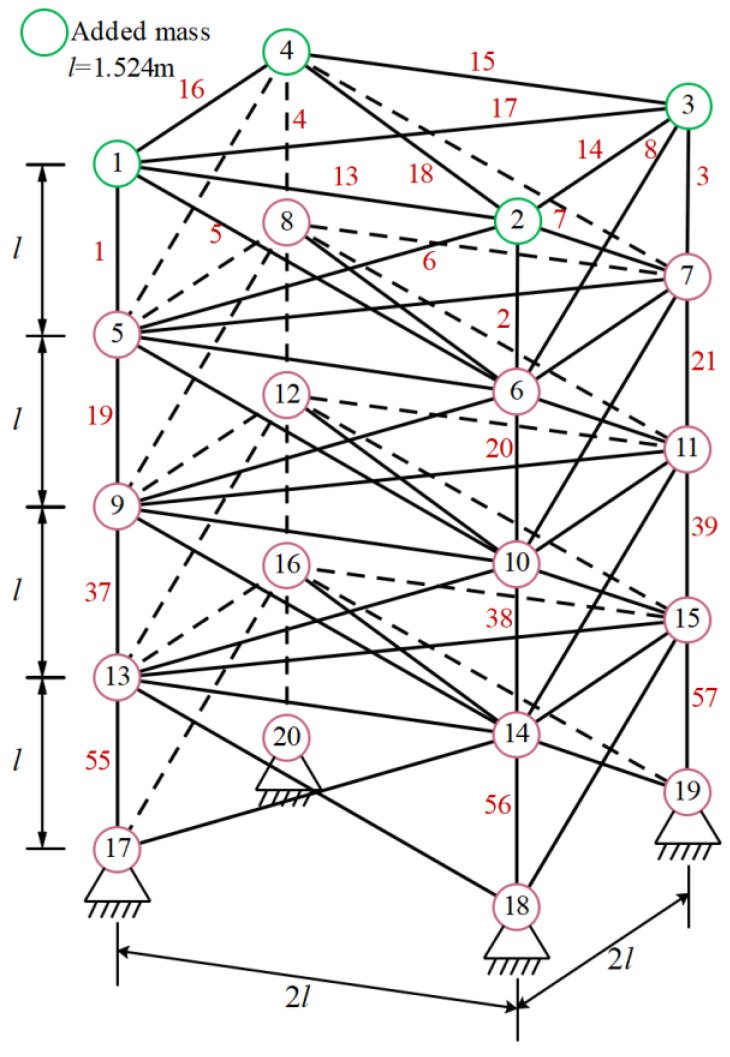
72 bar truss.

**Figure 7:**
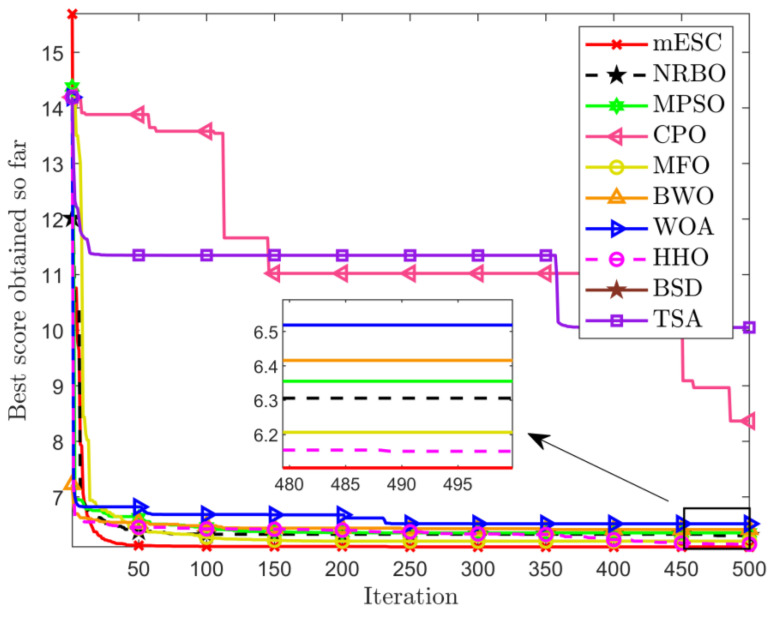
Convergence diagram of 72 bar truss.

**Figure 8:**
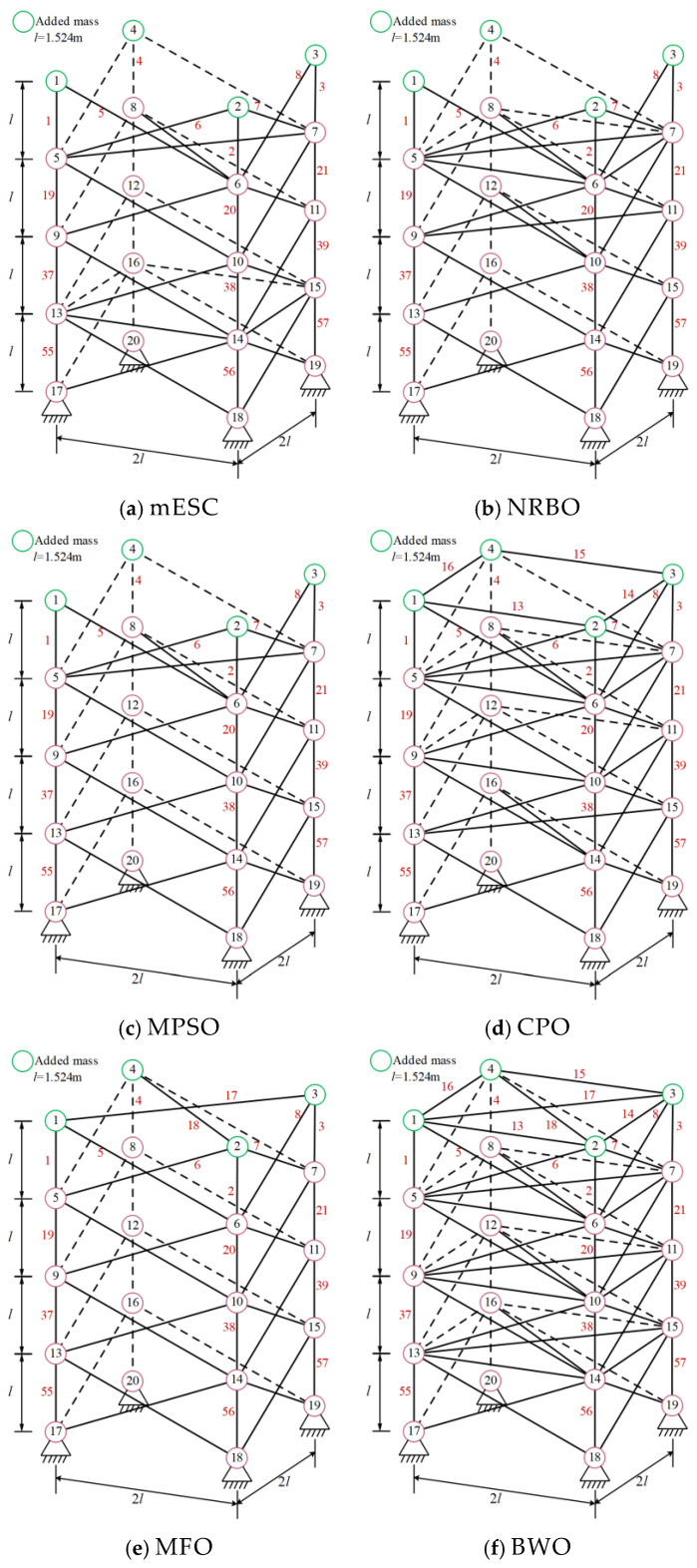

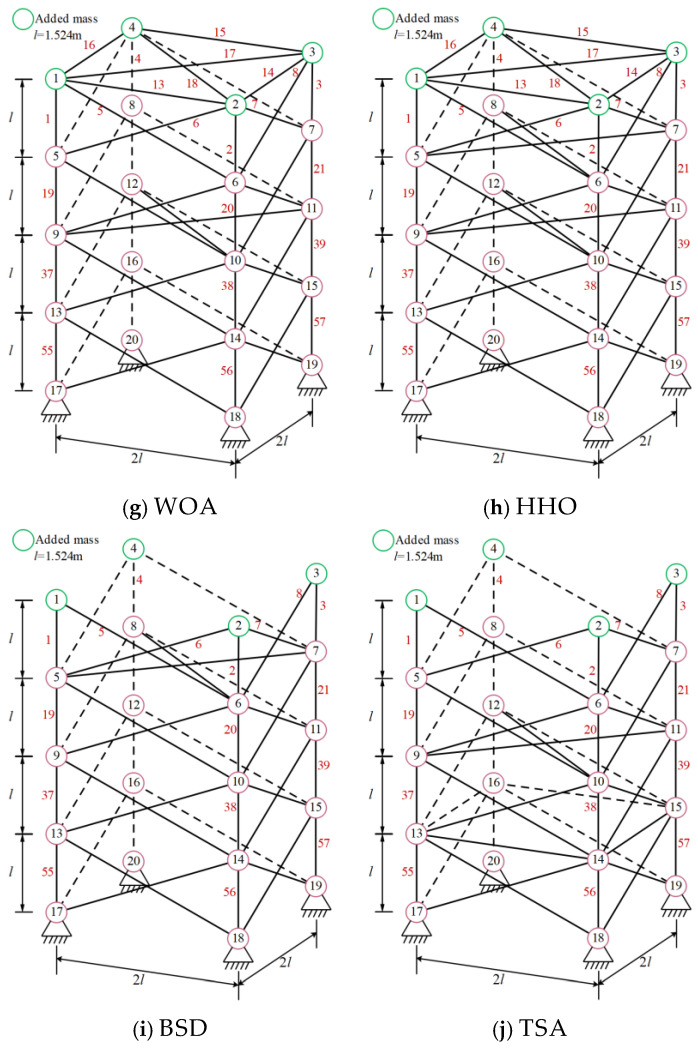
Topology optimization of 72 bar truss using various algorithms.

Figure 6 shows the 72 bar structural diagram. The truss components are divided into 16 groups, with the top four nodes representing mass concentration points. Set the data and parameters for minimum weight optimization in Table 5. The minimum weights and average values obtained by each algorithm are shown in Table 6. The optimal weight and overall average value of mESC are generally the best, with an optimal weight of 443.7325 being the smallest among all comparison algorithms, which confirms the superiority of the proposed algorithm’s performance. Figure 7 shows the optimization design convergence graphs of all algorithms, with mESC having the highest convergence accuracy. Figure 8 shows the truss structure diagram optimized by various algorithms.

mESC can achieve the optimal structure by removing six rods; mESC needs to excel in solving such problems.

### 3.2. Engineering Problem

We use mESC to solve five engineering optimizations, namely minimizing the weight of the reducer, designing the welding beam, the problem of the stepper cone pulley, the problem of robot clamping, and the rolling element bearing. This article uses static penalty methods to handle constraints in the above engineering optimization problems, with the specific formula being:

(22) $$\phi (r)=f(r)\pm \left[\sum_{j=1}^{m}h_{j}\cdot \max{\left(0,\;z_{j}(r)\right)}^{\varepsilon }+\sum_{k=1}^{n}i_{j}{\left|W_{j}(r)\right|}^{\varphi }\right].$$

In Formula (22), ϕ(r) is the objective function, $h_{j}$ and $i_{j}$ are positive penalty constants, $W_{j}$ and $z_{j}$ are constraints, and parameters ε and φ are 1 or 2.

#### 3.2.1. Minimize the Weight of the Reducer

This question is about the design of a small aircraft engine reducer, which involves seven variables: surface width $\left(c_{1}\right)$, tooth pattern $\left(c_{2}\right)$, number of teeth $\left(c_{3}\right)$ of the small gear, the size of the first axis is $\left(c_{4}\right)$, size $\left(c_{5}\right)$ of the other shaft, first shaft diameter $\left(c_{6}\right)$, and another shaft diameter $\left(c_{7}\right)$. Its characteristics are as follows: 

Minimize

(23) $$\begin{array}{l} f(\bar{c})=0.7854\;c_{2}^{2}c_{1}(14.9334\;c_{3}-43.0934+3.3333\;c_{3}^{2})\\ \;\;\;\;\;\;\;\;\;\;\;+0.7854\;(c_{5}c_{7}^{2}+c_{4}c_{6}^{2})-1.508\;c_{1}(c_{7}^{2}+c_{6}^{2})+7.477\;(c_{7}^{3}+c_{6}^{3}) \end{array}.$$

Constraints:

(24) $$\left\{\begin{array}{l} g_{1}(\bar{c})=-c_{1}c_{2}^{2}c_{3}+27\leq 0,\\ g_{2}(\bar{c})=-c_{1}c_{2}^{2}c_{3}^{2}+397.5\leq 0,\\ g_{3}(\bar{c})=-c_{2}c_{6}^{4}c_{3}c_{4}^{-2}+1.93\leq 0,\\ g_{4}(\bar{c})=-c_{2}c_{7}^{4}c_{3}c_{5}^{-3}+1.93\leq 0,\\ g_{5}(\bar{c})=10c_{6}^{-3}\sqrt{16.91\times 10^{6}+{\left(745c_{4}c_{2}^{-1}c_{3}^{-1}\right)}^{2}}-1100\leq 0,\\ g_{6}(\bar{c})=10c_{7}^{-3}\sqrt{157.5\times 10^{6}+{\left(745c_{5}c_{2}^{-1}c_{3}^{-1}\right)}^{2}}-850\leq 0,\\ g_{7}(\bar{c})=c_{2}c_{3}-40\leq 0,\\ g_{8}(\bar{c})=-c_{1}c_{2}^{-1}+5\leq 0,\\ g_{9}(\bar{c})=c_{1}c_{2}^{-1}-12\leq 0,\\ g_{10}(\bar{c})=1.5c_{6}-c_{4}+1.9\leq 0,\\ g_{11}(\bar{c})=1.1c_{7}-c_{5}+1.9\leq 0. \end{array}\right.$$

Range:

(25) $$\left\{\begin{array}{l} 0.7\leq c_{2}\leq 0.8,\;17\leq c_{3}\leq 28,\;2.6\leq c_{1}\leq 3.6,\\ 5\leq c_{7}\leq 5.5,\;7.3\leq c_{5},\;c_{4}\leq 8.3,\;2.9\leq c_{6}\leq 3.9. \end{array}\right.$$

To solve this problem, mESC conducted comparative experiments with NRBO, MPSO, CPO, BWO, WOA, HHO, TSA, AO [54], and GWO. According to Table 7, mESC provides the optimal design with an optimal value of 2994.506339787.

#### 3.2.2. Welding Beam Design

**Figure 9:**
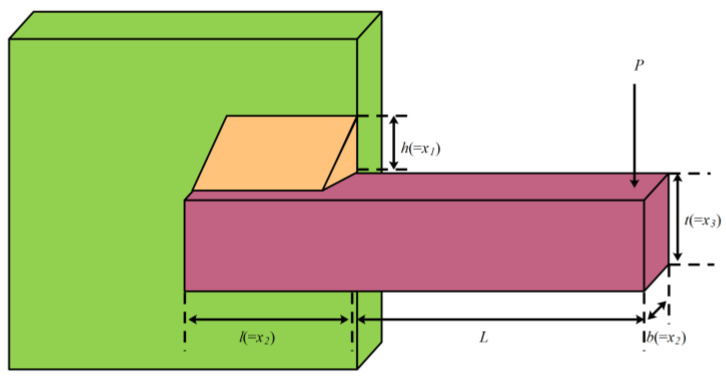
Welded beam structure.

This design aims to minimize the cost of welding beams [55], involving weld seam width $h(c_{1})$, clamping rod length $l(c_{2})$, rod height $t(c_{3})$, and rod thickness $b(c_{4})$. The schematic diagram is shown in Figure 9, and the features are as follows: 

Minimize

(26) $$f(\bar{c})=1.10471c_{1}^{2}c_{2}+0.04811c_{3}c_{4}(14.0+c_{2}).$$

Constraints:

(27) $$\left\{\begin{array}{l} g_{1}(\bar{c})=c_{1}-c_{4}\leq 0,\\ g_{2}(\bar{c})=\delta (c)-\delta_{\max}\leq 0,\\ g_{3}(\bar{c})=P\leq P_{c}(c),\\ g_{4}(\bar{c})=\tau_{\max}\geq \tau (c),\\ g_{5}(\bar{c})=\sigma (c)-\sigma_{\max}\leq 0. \end{array}\right.$$

Range:

(28) $$\left\{\begin{array}{l} 0.1\leq c_{3},c_{2}\leq 10,0.1\leq c_{4}\leq 2,0.125\leq c_{1}\leq 2,\delta_{\max}=0.25in,\\ \tau =\sqrt{{\tau^{\prime }}^{2}+{\tau^{\prime \prime }}^{2}+2\tau^{\prime }\tau^{\prime \prime }\frac{c_{2}}{2R}},\tau^{\prime \prime }=\frac{RM}{J},\tau^{\prime }=\frac{P}{\sqrt{2}c_{2}c_{1}},\\ M=P(\frac{c_{2}}{2}+L),R=\sqrt{\frac{c_{2}^{2}}{4}+{\left(\frac{c_{1}+c_{3}}{2}\right)}^{2}},\sigma (\bar{c})=\frac{6PL^{3}}{Ec_{3}^{2}c_{4}},\\ J=2((\frac{c_{2}^{2}}{4}+{\left(\frac{c_{1}+c_{3}}{2}\right)}^{2})\sqrt{2}c_{1}c_{2}),P_{x}(\bar{c})=\frac{4.013Ec_{3}c_{4}^{3}}{6L^{2}}(1-\frac{c_{3}}{2L}\sqrt{\frac{E}{4G}}),\\ L=14in,P=6000lb,E=30.10psi,\sigma_{\max}=13600psi,G=12\cdot 10^{6}psi. \end{array}\right.$$

To solve this problem, mESC conducted comparative experiments with NRBO, MPSO, CPO, BWO, WOA, HHO, TSA, AO, and GWO. The optimal value obtained by mESC on this problem in Table 8 is 1.670306973.

#### 3.2.3. Step Cone Pulley Problem

**Figure 10:**
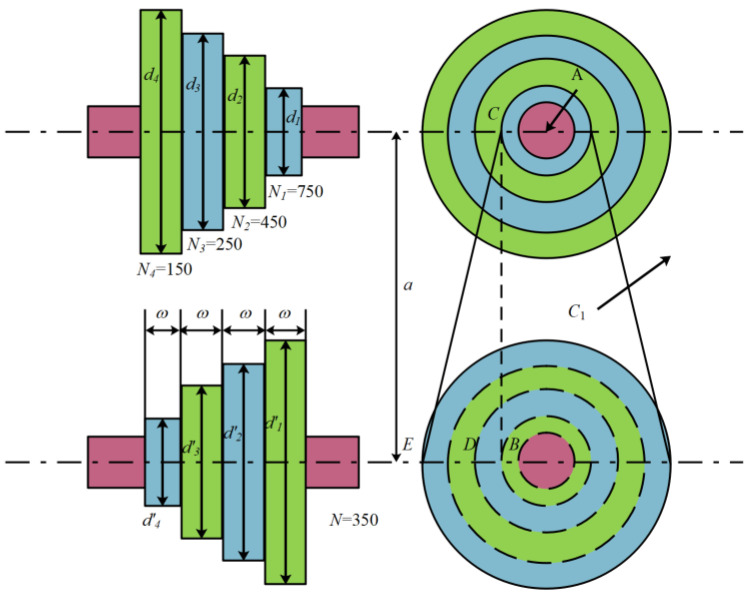
Stepping cone pulley.

The problem is to minimize the weight of the stepping cone pulley [56], involving five variables: pulley diameter and pulley width. Figure 10 shows its design diagram: 

Minimize

(29) $$f(\bar{c})=\rho \omega \left[\begin{array}{c} d_{1}^{2}\left\{11+{\left(\frac{N_{1}}{N}\right)}^{2}\right\}+d_{2}^{2}\left\{1+{\left(\frac{N_{2}}{N}\right)}^{2}\right\}+d_{3}^{2}\left\{1+{\left(\frac{N_{3}}{N}\right)}^{2}\right\}\\ +d_{4}^{2}\left\{1+{\left(\frac{N_{4}}{N}\right)}^{2}\right\} \end{array}\right].$$

Constraints:

(30) $$\left\{\begin{array}{l} h_{1}(\bar{c})=X_{1}-X_{2}=0,h_{2}(\bar{c})=X_{1}-X_{3}=0,h_{3}(\bar{c})=X_{1}-X_{4}=0,\\ g_{i=1,2,3,4}(\bar{c})=-R\leq 2,g_{i=5,6,7,8}(\bar{c})=(0.75\times 745.6998)-P_{i}\leq 0. \end{array}\right.$$

Among them,

(31) $$\left\{\begin{array}{l} X_{i}=\frac{\pi d_{i}}{2}(1+\frac{N_{i}}{N})+\frac{{\left(\frac{N_{i}}{N}\right)}^{2}}{4a}+2a,i=(1,2,3,4),\\ R_{i}=\exp\left[\mu \left\{\pi -2\sin^{-1}\left\{(\frac{N_{i}}{N}-1)\frac{d_{i}}{2a}\right\}\right\}\right],i=(1,2,3,4),\\ P_{i}=st\omega (1-R_{i})\frac{\pi d_{i}N_{i}}{60},i=(1,2,3,4),t=8\mathrm{mm},s=1.75\mathrm{MPa},\mu =0.35,\\ \rho =7200\mathrm{kg}/m^{3},a=3\mathrm{mm}. \end{array}\right.$$

To address this issue, comparative experiments were conducted in Table 9 between mESC and competitors such as MPSO, CPO, AVOA [57], BWO, WOA, HHO, TSA, AO, and GWO. The results showed that mESC achieved the best performance with an optimal value of 16.983218316.

#### 3.2.4. Robot Clamping Problem

**Figure 11:**
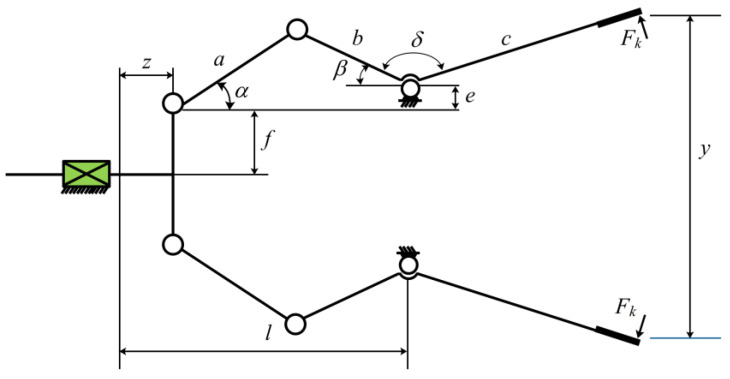
Robot clamping structure.

This task studies the force that robot grippers can generate when grasping objects, while ensuring that the objects are not damaged and the grasping is stable [58]. Figure 11 is a structural diagram, characterized by the following: 

Minimize

(32) $$f(\bar{c})=\underset{z}{-\min}F_{k}(c,z)\underset{z}{+\max}F_{k}(c,z).$$

Constraints:

(33) $$\left\{\begin{array}{l} g_{1}(\bar{c})=-Y_{\min}+y(\bar{c},Z_{\max})\leq 0,g_{2}(\bar{c})=-y(c,Z_{\max})\leq 0,\\ g_{3}(\bar{c})=Y_{\max}-y(\bar{c},0)\leq 0,g_{4}(\bar{c})=y(\bar{c},0)-Y_{G}\leq 0,\\ g_{5}(\bar{c})=l^{2}+e^{2}-{\left(a+b\right)}^{2}<2,g_{6}(\bar{c})=b^{2}-{\left(a-e\right)}^{2}-{\left(l-Z\max\right)}^{2}\leq 0,\\ g_{7}(\bar{c})=Z_{\max}-l\leq 0. \end{array}\right.$$

Among them,

(34) $$\left\{\begin{array}{l} \alpha =\cos^{-1}(\frac{a^{2}+g^{2}-b^{2}}{2ag})+\varphi ,g=\sqrt{e+{\left(z^{2}-l\right)}^{2}},\\ \beta =\cos^{-1}(\frac{b^{2}+g^{2}-a^{2}}{2bg})-\varphi ,\varphi =\tan^{-1}(\frac{e}{l-z}),\\ y(c,z)=2(f+e+x\sin(\beta +\delta )),F_{k}=Pb\sin(\alpha +\beta )/2x\cos(\alpha ),\\ Y_{\min}=50,Y_{\max}=100,Y_{G}=150,Z_{\max}=100,P=100. \end{array}\right.$$

Range:

(35) $$\left\{\begin{array}{l} 0\leq e\leq 50,100\leq x\leq 200,10\leq f,a,b\leq 150,\\ 1\leq \delta \leq 3.14,100\leq l\leq 300. \end{array}\right.$$

To address this issue, mESC conducted comparative experiments with MPSO, CPO, AVOA, BWO, WOA, HHO, TSA, AO, and GWO. Table 10 presents the design results, and mESC achieved the best performance with an optimal value of 16.983218316.

#### 3.2.5. Rolling Element Bearings

**Figure 12:**
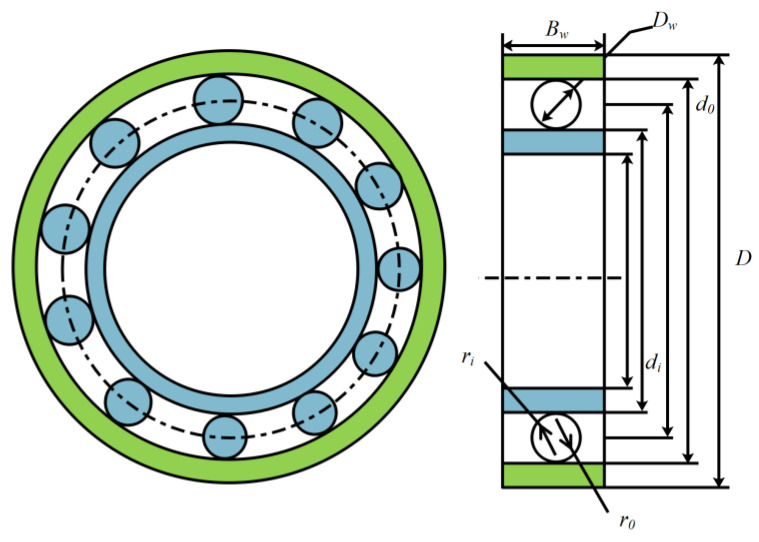
Rolling element bearing structure.

This article applies mESC to optimize the design of rolling bearings. The schematic diagram is shown in Figure 12, with a total of 10 optimization parameters and the following characteristics: 

Minimize

(36) $$f(\bar{c})=\left\{\begin{array}{l} f_{x}Z^{2/3}Db^{1.8}\;\;\;\;\;\;\;\;\;\;\;,\;if\;Db\leq 25.4\;mm,\\ 3.647f_{x}Z^{2/3}Db^{1.4}\;\;,\;othwewise. \end{array}\right.$$

Constraints:

(37) $$\left\{\begin{array}{l} g_{1}(\bar{c})=Z-\varphi_{0}/2\sin^{-1}(Db/Dm)-1\leq 0,\\ g_{2}(\bar{c})=K_{D\min}(D-d)-2Db\leq 0,\\ g_{3}(\bar{c})=2Db-K_{D\min}(D-d)\leq 0,\\ g_{4}(\bar{c})=Db-w\leq 0,g_{5}(\bar{c})=0.5(D+d)-Dm\leq 0,\\ g_{6}(\bar{c})=Dm-(0.5+e)(D+d)\leq 0,\\ g_{7}(\bar{c})=\varepsilon Db-0.5(D-Dm-Db)\leq 0,g_{8}(\bar{c})=0.515-f_{i}\leq 0,\\ g_{9}(\bar{c})=0.515-f_{0}\leq 0. \end{array}\right.$$

where

(38) $$\left\{\begin{array}{l} f_{x}=37.91{\left\{1+{\left\{1.04{\left(\frac{1-\gamma }{1+\gamma }\right)}^{1.72}{\left(\frac{f_{i}(2f_{0}-1)}{f_{0}(2f_{i}-1)}\right)}^{0.41}\right\}}^{10/3}\right\}}^{-0.3},\\ \gamma =\frac{Db\cos(\alpha )}{Dm},f_{i}=\frac{r_{i}}{Db},f_{0}=\frac{r_{0}}{Db},\\ \varphi_{0}=2\pi -2\cos^{-1}(\frac{{\left\{\left(D-d\right)/2-3(T/4)\right\}}^{2}+{\left\{D/2-(T/4)-Db\right\}}^{2}}{2\left\{\left(D-b\right)/2-3(T/4)\right\}}\\ \frac{-{\left\{d/2+(T/4)\right\}}^{2}}{\left\{D/2-(T/4)-Db\right\}}),\\ T-D-d-2Db,D=160,d=90,B_{w}=30. \end{array}\right.$$

Range:

(39) $$\left\{\begin{array}{l} 0.5(D+d)<Dm<0.6(D+d),0.15(D-d)<Db<0.45(D-d),\\ 4\leq Z\leq 50,0.515\leq f_{0}\leq 0.6,0.4\leq K_{D\min}\leq 0.5,0.6\leq K_{D\max}\leq 0.7,\\ 0.3\leq \varepsilon \leq 0.4,0.02\leq e\leq 0.1,0.6\leq \zeta \leq 0.85. \end{array}\right.$$

To solve this problem, mESC was compared with MPSO, CPO, AVOA, BWO, WOA, HHO, TSA, AO, and GWO in experimental experiments. mESC can achieve optimal performance levels, and the optimal value of 16,958.202286941 is given in Table 11.

## 4. Summarize

This study proposes an improved version of mESC based on multi-strategy enhancement, which maintains population diversity through adaptive perturbation factor strategy, restarts the mechanism to improve the global exploration of mESC, and balances local development of the algorithm through dynamic centroid reverse learning strategy. Finally, the elite pool boundary adjustment strategy is used to accelerate population convergence. mESC conducted performance tests on the test suite and six optimized designs to demonstrate its strong superiority. In the future, we will further expand mESC, research new population update mechanisms, and apply them in areas such as feature selection, image segmentation, and information processing.

## Figures and Tables

**Table 1:** Parameter settings.

Algorithms	Parameter	Value
PO	r: random number	r∈[0, 1]
GMO	limvel: the ratio of distance to speed	limvel=0.1
	ε: parameter	ε=0
FATA	θ: parameter	θ∈[0, 1]
	α: the transformation mode of individual light	α∈[0, 1]
MGO	dn: number of divisions	dn=D/4 and dn≥1
	$r_{1\text{–}6}$: random number	$r_{1\text{–}6}\in (0,1)$
	$d_{1}$: constant parameter	$d_{1}=0.2$
	$w,d_{2}$: constant parameter	w=2, $d_{2}=0.5$
CPO	$\vec{r}$: random number	r∈[0, 1]
	$\tau_{1}$, $\tau_{2}$, $\tau_{3}$$r_{1},\;r_{2}$: random number	$\tau_{1}$: random numbers based on normal distribution, $\tau_{2}\in [0,\;1]$, $\tau_{3}\in [0,\;1]$, $r_{1},\;r_{2}\in [1,Num]$
PLO	$r_{5}$: parameter	$r_{5}<0.05$
NRBO	r: random number	r∈(0, 1)
	δ: balance parameter	δ∈[−1, 1]
IAO	θ,rand,σ,ε,ς,κ,ω: random number	θ,rand,σ,ε,ς,κ,ω∈[0,1]
LEA	$\lambda_{c}$: acceptance rate	$\lambda_{c}=0.5$
ESC	$Rand_{i,d}$: random number	$Rand_{i,d}\in (0,\;1)$
	a: proportion of calm group	a=0.15
	b: proportion of aggregation groups	b=0.35
	c: proportion of panic group	c=0.5
	$m_{1},\;m_{2}$: binary variable	$m_{1},\;m_{2}=0\;or\;1$
AGPSO	C1, C2: learning factor	C1=C2=2
	$\omega_{\max},$$\omega_{\min}$: maximum/minimum weight value	$\omega_{\max}=0.9$, $\omega_{\min}=0.4$
CPSOGSA	phi1,phi2: shrinkage coefficient	phi1=2.05, phi2=2.05
	phi	phi1+phi2
	ω: weight	$\omega =2/(phi-2+\sqrt{phi^{2}-4phi})$
	$\omega_{damp}$: weight damping ratio	$\omega_{damp}=1$
	C1,C2: learning factor	C1=ω⋅phi1, C2=ω⋅phi2
TACPSO	$\omega_{\max}$, $\omega_{\min}$: maximum/minimum weight value	$\omega_{\max}=0.9$, $\omega_{\min}=0.4$
	C1, C2: learning factor	C1=C2=2
BDE	*C*: crossover probability	*C* = 0.2
	*F*: scale factor	*F* = 0.5
BeSD	K: parameter	K=5
	Up: upper boundary	Up=100
	Low: lower boundary	Low=-100
MDE	C: crossover probability	C=0.2
	F: scale factor	F=0.5
MadDE	$q_{rate}$: optimal cross probability in personal history	$q_{rate}=0.01$
	$pb_{rate}$: optimal probability of personal history	$pb_{rate}=0.18$
	$arc_{rate}$: archiving rate	$arc_{rate}=2.3$
	$memory_{size}$: size	$memory_{size}=2.3$
	$\max pop_{size}$: maximum population size	$\max pop_{size}=30$
	$\min pop_{size}$: minimum population size	$\min pop_{size}=4$
LSHADE-cnEpSin	μF, μCR, μfreq	μF=μCR=μfreq=0.5
	r: parameter	r=1.4
	p	p=0.11
	H	H=0.5
	$G_{ls}$	$G_{ls}=250$
LSHADE-SPACMA	F: scale factor	F=0.5
	H: historical memory size	H=5
	C: crossover probability	C=0.8
	p: probability of initial control mutation strategy	p=0.3
	p: probability of minimum control mutation strategy	p=0.15
LSHADE	p: probability of controlling mutation strategy	p=0.11
	H: historical memory size	H=5
	$MaxPop_{size}$: maximum population	$MaxPop_{size}=30$
	$MinPop_{size}$: minimum population	$MinPop_{size}=4$
MPSO	$\omega_{\max}$, $\omega_{\min}$: maximum/minimum weight value	$\omega_{\max}=0.9$, $\omega_{\min}=0.4$
	C1, C2: learning factor	C1=2, C2=2
MFO	t: randomly generated number	t∈[−1, 1]
BWO	$B_{0}$	Random numbers between (0, 1)
	p	Random numbers between [0, D]
	$r_{1\text{–}7}$	Random numbers between (0,1)
	$W_{f}$: probability of falling	Decrease from 0.1 to 0.05
WOA	rand: random number	rand∈(0,1)
	a: parameter	a: reduce from 2 to 0
	A: contraction unit	A∈[−a,a]
	l: parameter	l∈[−1,1]
HHO	R: random number	R∈[0,1]
	J: random number	J∈[0,2]
BSD	r: random number	r∈[0, 1]
TSA	rand: random number	rand∈(0,1)
AO	rand: random number	rand∈(0,1)
	s: random number	s=0.01
	u,v: parameter	u and v follow the Gaussian distribution of $N(0,\sigma^{2})$ and N(0,1) random numbers
	β: random number	s=1.5
GWO	a: parameter	a: reduce from 2 to 0
	$r_{1},\;r_{2}$: parameter	$r_{1},\;r_{2}\in (0,1)$
AVOA	$L_{1},\;L_{2}$: parameter	$L_{1},\;L_{2}\in (0,1)$
	z: random number	z∈[−1,1]
	h: random number	h∈[−2,2]
	$k_{1},k_{2},k_{3},k_{4},k_{5},k_{p1},kp_{2}$: random number	$k_{1},k_{2},k_{3},k_{4},k_{5},k_{p1},kp_{2}\in [0,1]$
MNEARO	L: step factor	L=0.1
AHA	N: flight step length	N=0.1
	M: memory coefficient	M=0.5
DMOA	$M_{S}$: moving step size	$M_{S}=0.1$
	$V_{f}$: warning factor	$V_{f}=0.5$
SHO	$F_{f}$: foraging parameters	$F_{f}=0.5$
	λ: random number	λ∈(0,2)
ZOA	$C_{F}$: ethnic cognition	$C_{F}=0.5$

**Table 2:** Experimental results of ESC, mESC, mESC1, mESC2, mESC3, and mESC4 on CEC2022.

F	Index	ESC	mESC1	mESC2	mESC3	mESC4	mESC
1	Mean	1.5042 × 10^4^	1.5805 × 10^4^	1.4008 × 10^4^	1.2580 × 10^4^	2.7391 × 10^3^	1.2049 × 10^3^
	Std	6.4060 × 10^3^	6.9949 × 10^3^	7.5581 × 10^3^	3.2190 × 10^3^	1.7844 × 10^3^	1.1660 × 10^3^
2	Mean	4.6222 × 10^2^	4.5643 × 10^2^	4.5313 × 10^2^	4.6310 × 10^2^	4.5293 × 10^2^	4.5191 × 10^2^
	Std	1.3323 × 10^1^	9.5756 × 10^0^	1.3892 × 10^1^	1.3851 × 10^1^	8.8945 × 10^0^	9.6902 × 10^0^
3	Mean	6.0001 × 10^2^	6.0001 × 10^2^	6.0001 × 10^2^	6.0000 × 10^2^	6.0000 × 10^2^	6.0000 × 10^2^
	Std	1.6406 × 10^−2^	2.6841 × 10^−2^	1.9361 × 10^−2^	1.5377 × 10^−2^	1.2641 × 10^−4^	8.2641 × 10^−5^
4	Mean	8.6415 × 10^2^	8.6447 × 10^2^	8.3105 × 10^2^	8.6223 × 10^2^	8.6080 × 10^2^	8.2202 × 10^2^
	Std	9.3537 × 10^0^	7.6704 × 10^0^	1.1628 × 10^1^	8.5616 × 10^0^	1.0106 × 10^1^	8.9211 × 10^0^
5	Mean	9.0083 × 10^2^	9.0082 × 10^2^	9.0279 × 10^2^	9.0069 × 10^2^	9.0013 × 10^2^	9.0005 × 10^2^
	Std	8.7322 × 10^−1^	1.2167 × 10^0^	7.1340 × 10^0^	1.2801 × 10^0^	5.1178 × 10^−1^	1.7219 × 10^−1^
6	Mean	4.7675 × 10^3^	5.6998 × 10^3^	4.7194 × 10^3^	4.1786 × 10^3^	4.4762 × 10^3^	4.8843 × 10^3^
	Std	3.7426 × 10^3^	3.3620 × 10^3^	3.7923 × 10^3^	2.8831 × 10^3^	2.4457 × 10^3^	2.8294 × 10^3^
7	Mean	2.0357 × 10^3^	2.0386 × 10^3^	2.0532 × 10^3^	2.0357 × 10^3^	2.0340 × 10^3^	2.0292 × 10^3^
	Std	8.8387 × 10^0^	7.7590 × 10^0^	3.7749 × 10^1^	9.6595 × 10^0^	5.8826 × 10^0^	1.0311 × 10^1^
8	Mean	2.2311 × 10^3^	2.2310 × 10^3^	2.2307 × 10^3^	2.2300 × 10^3^	2.2351 × 10^3^	2.2218 × 10^3^
	Std	2.0788 × 10^0^	1.7741 × 10^0^	3.2975 × 10^1^	1.6037 × 10^0^	2.1735 × 10^1^	1.0352 × 10^0^
9	Mean	2.4830 × 10^3^	2.4822 × 10^3^	2.4808 × 10^3^	2.4837 × 10^3^	2.4810 × 10^3^	2.4806 × 10^3^
	Std	2.7814 × 10^0^	1.5672 × 10^0^	1.6116 × 10^−2^	2.2597 × 10^0^	7.2857 × 10^−1^	6.7340 × 10^−1^
10	Mean	2.5808 × 10^3^	2.5217 × 10^3^	2.6196 × 10^3^	2.5283 × 10^3^	2.5924 × 10^3^	2.5219 × 10^3^
	Std	1.1585 × 10^2^	6.1119 × 10^1^	1.7712 × 10^2^	8.6926 × 10^1^	1.4213 × 10^2^	8.7564 × 10^1^
11	Mean	2.9083 × 10^3^	2.9001 × 10^3^	2.9033 × 10^3^	2.9012 × 10^3^	2.9162 × 10^3^	2.9000 × 10^3^
	Std	3.1664 × 10^1^	3.9569 × 10^−2^	6.6868 × 10^1^	6.4979 × 10^1^	4.6994 × 10^1^	6.4327 × 10^1^
12	Mean	2.9494 × 10^3^	2.9460 × 10^3^	2.9545 × 10^3^	2.9533 × 10^3^	2.9441 × 10^3^	2.9437 × 10^3^
	Std	9.0245 × 10^0^	6.4685 × 10^0^	1.0251 × 10^1^	7.6731 × 10^0^	7.2801 × 10^0^	7.6958 × 10^0^

**Table 3:** Conditions for 24 bar truss structure.

Parameter	Value
Design variable	$A_{i},i=1,\;2,\;\ldots ,\;24$
Two types of load conditions	Condition 1: $F1=5\times 10^{4}\;N,\;F2=0$ Condition 2: $F1=0,\;F2=5\times 10^{4}\;N$
Centralized quality of node 3	500 kg
Stress constraint	$\sigma_{i}^{\max}=173.43\;MPa$
Displacement constraint	$\delta_{5y\&6y}^{\max}=10\;mm$
Natural frequency constraint	$f_{1}\geq 30\;Hz$
Continuous cross-sectional area	$[A_{lower},\;A_{upper}]=[-40,\;40]\;cm^{2},$ Key areas: $1\;cm^{2}$
Material characteristics	$E=6.9\times 10^{10}\;Pa\;and\;\rho =2740\;kg/m^{3}$

**Table 4:** Optimization results of 24 bars.

Variable	mESC	NRBO	MPSO	CPO	MFO	BWO	WOA	HHO	BSD	TSA
A_1_ (cm^2^)	-	1.1809	-	21.7301	-	11.8313	-	9.9764	-	5.6128
A_2_ (cm^2^)	-	-	0.1253	18.6976	-	18.7441	-	-	8.0505	-
A_3_ (cm^2^)	-	-	-	25.8991	-	7.0705	27.0394	4.8722	-	0.9070
A_4_ (cm^2^)	-	-	-	0.17802	-	20.5284	-	-	-	-
A_5_ (cm^2^)	-	-	-	-	-	-	16.2991	-	-	1.4330
A_6_ (cm^2^)	-	22.7865	-	31.7649	0.2929	9.7776	1.5009	11.1758	5.3444	3.3680
A_7_ (cm^2^)	19.7054	25.4245	21.3769	13.1510	22.4198	15.9891	20.7110	18.3170	24.6264	19.1069
A_8_ (cm^2^)	3.4372	10.2396	3.0964	6.5304	7.2682	13.6646	13.2598	13.6893	6.2642	4.7147
A_9_ (cm^2^)	2.2248	-	-	27.0153	15.9891	8.5750	-	-	-	-
A_10_ (cm^2^)	-	2.4959	1.3984	39.3547	-	15.9998	34.4416	-	1.7461	4.7910
A_11_ (cm^2^)	-	-	-	-	-	-	-	-	-	-
A_12_ (cm^2^)	4.0914	-	-	25.9631	21.1751	14.0195	-	3.2835	-	-
A_13_ (cm^2^)	13.8386	20.4357	17.8990	-	-	11.7006	19.0629	15.6416	24.1503	17.4419
A_14_ (cm^2^)	-	1.0096	-	2.7350	-	-	-	6.6755	11.6320	3.4732
A_15_ (cm^2^)	3.7369	9.5323	3.8540	3.6931	7.6206	7.4739	12.7173	8.3768	6.1346	4.1225
A_16_ (cm^2^)	23.8717	23.5038	35.01534	28.3814	26.3117	18.9343	32.2477	23.5163	25.1303	24.2617
A_17_ (cm^2^)	-	1.4094	-	10.5085	-	13.3664	-	14.3179	-	-
A_18_ (cm^2^)	-	1.1537	-	-	-	7.4196	-	-	-	-
A_19_ (cm^2^)	-	0.1521	-	-	-	-	-	2.8833	-	0.5071
A_20_ (cm^2^)	-	-	-	-	-	4.8082	-	-	-	-
A_21_ (cm^2^)	-	-	-	14.4812	-	19.3918	4.3067	-	-	-
A_22_ (cm^2^)	0.9365	3.8747	13.1986	-	2.4049	8.2420	-	11.5827	6.7845	3.0967
A_23_ (cm^2^)	-	0.5766	1.8418	18.2226	-	-	13.9150	1.8968	2.1376	2.6050
A_24_ (cm^2^)	0.9666	-	-	18.2264	17.9944	-	2.0243	10.4043	-	2.3582
Optimal weight	121.5840	182.1442	149.3737	357.1651	150.0160	308.8677	309.0326	206.3347	185.6925	159.9285
Mean	140.5701	247.1569	207.9751	5576.3910	198.1633	366.5999	492.0880	315.3927	233.8727	309.2789

**Table 5:** Setting of 72 bar truss structure.

Parameter	Value
Design variable	$G_{i},i=1,\;2,\;\ldots ,\;16$
Two types of load conditions	Condition 1: $F_{1x}=F_{1y}=22.25\;kN,F_{1z}=-22.25\;kN$ Condition 2: $F_{1z}=F_{2z}=F_{3z}=F_{4z}=-22.25\;kN$
Centralized quality of nodes 1, 2, 3, and 4	2270 kg
Stress constraint	$\sigma_{i}^{\max}=172.375\;MPa$
Displacement constraint	$\delta_{1x\&1y\&2x\&2y\&3x\&3y\&4x\&4y}^{\max}=6.35\;mm$
Natural frequency constraint	$f_{1}\geq 4\;Hz\;and\;f_{3}\geq 6\;Hz$
Continuous cross-sectional area	$[A_{lower},\;A_{upper}]=[-30,\;30]\;cm^{2},$ Key areas: $1\;cm^{2}$
Material characteristics	$E=6.895\times 10^{10}\;Pa\;and\;\rho =2767.99\;kg/m^{3}$

**Table 6:** Optimization of 72 bar truss structure.

Group	Member	mESC	NRBO	MPSO	CPO	MFO	BWO	WOA	HHO	BSD	TSA
G_1_ (cm^2^)	A_1_~A_4_	5.4549	10.4574	30.0000	4.5010	4.8495	9.8287	16.3827	4.7008	4.7576	5.3634
G_2_ (cm^2^)	A_5_~A_12_	11.0255	10.6118	11.1276	22.8647	7.9218	7.7810	6.3131	8.2657	11.4430	13.2010
_G3_ (cm^2^)	A_13_~A_16_	-	-	-	10.9710	-	8.4782	6.5049	5.0809	-	-
G_4_ (cm^2^)	A_17_~A_18_	-	-	-	-	9.6046	7.9046	18.1787	7.5606	-	-
G_5_ (cm^2^)	A_19_~A_22_	9.2063	9.2986	6.3548	4.2146	6.8508	9.1444	10.2818	8.0596	9.4020	4.6674
G_6_ (cm^2^)	A_23_~A_30_	7.4037	6.8758	8.1408	11.5261	8.6174	8.1201	11.5043	7.5126	8.8709	13.1645
G_7_ (cm^2^)	A_31_~A_34_	-	5.4373	-	2.7353	-	7.0007	-	-	-	-
G_8_ (cm^2^)	A_35_~A_36_	3.8121	16.3016	30.0000	11.2471	-	8.0223	-	3.4783	4.4508	-
G_9_ (cm^2^)	A_37_~A_40_	11.8461	15.4211	30.0000	26.1691	30.0000	11.5553	10.2417	15.7884	12.6652	16.6723
G_10_ (cm^2^)	A_41_~A_48_	8.0648	8.5348	7.5842	8.8167	8.6185	7.9849	8.8318	9.4454	8.0508	10.2219
G_11_ (cm^2^)	A_49_~A_52_	-	-	-	27.3933	-	8.0350	-	-	-	-
G_12_ (cm^2^)	A_53_~A_54_	-	17.4406	-	-	-	7.4099	12.0765	0.2841	-	9.9491
G_13_ (cm^2^)	A_55_~A_58_	15.5720	18.7970	12.2731	11.2987	12.4111	14.6300	15.6152	15.2107	14.2131	22.4342
G_14_ (cm^2^)	A_59_~A_66_	8.0085	6.8480	8.4865	15.7799	8.8328	8.5022	8.9845	7.8103	8.4783	7.1551
G_15_ (cm^2^)	A_67_~A_70_	1.8446	-	-	-	-	8.2163	-	-	-	4.1077
G_16_ (cm^2^)	A_71_~A_72_	-	-	-	16.0972	-	7.5293	-	-	-	-
Optimal weight		443.7325	541.6204	567.3181	833.1809	479.0954	605.2332	554.6159	466.3894	450.3880	3261.9035
Mean		447.1184	547.7935	575.5373	4305.5896	495.9691	611.5502	677.8491	469.2699	452.0753	23,188.7348

**Table 7:** Optimization results of reducer design.

Algorithms	Optimize Variables							Optimal Cost
	*C*_1_	*C*_2_	*C*_3_	*C*_4_	*C*_5_	*C*_6_	*C*_7_	
NRBO	3.576293284	0.700000000	17.000000000	8.095331344	8.128172008	3.352104593	5.287471843	3041.390312214
MPSO	3.600000000	0.800000000	17.000000000	7.300000000	7.715608415	3.348967058	5.286351002	3568.813527023
CPO	3.589888581	0.716865610	17.350743923	7.941594534	7.974287014	3.374738083	5.300298355	3200.926055200
BWO	3.517889078	0.700000000	17.000000000	7.300000000	7.979192990	3.405074109	5.289143425	3022.944192861
WOA	3.500000000	0.700000000	17.594707670	8.050855405	8.060485813	3.801066613	5.333459419	3275.837132091
HHO	3.598808155	0.700000000	17.000000000	7.444229405	8.118994668	3.359034412	5.286797391	3045.625326530
TSA	3.513466952	0.700000000	17.000000000	7.300000000	8.129914676	3.369685411	5.323369593	3037.320035061
AO	3.509108162	0.700000000	17.010098125	8.236044343	8.245358394	3.363003544	5.302138926	3032.817963447
GWO	3.502062907	0.700000000	17.000000000	7.512262443	7.869983676	3.353222591	5.287004717	3001.411114536
mESC	3.500063221	0.700012644	17.000000000	7.300000000	7.715319953	3.350540703	5.286654418	2994.506339787

**Table 8:** Optimization results of welded beam design.

Algorithms	Optimize Variables				Optimal Cost
	*C*_1_	*C*_2_	*C*_3_	*C*_4_	
NRBO	0.195353623	3.197843112	9.999929574	0.195353623	1.751139663
MPSO	0.198832307	3.337365299	9.192024322	0.198832307	1.670217726
CPO	0.174315932	4.852884341	9.070020775	0.274867853	2.424133890
BWO	0.125000000	5.909953669	8.979393453	0.208838326	1.898245842
WOA	0.125424542	5.683907479	9.451223190	0.197651098	1.867802825
HHO	0.192039362	3.467159434	9.204138913	0.199458436	1.683996291
TSA	0.198516892	3.367789251	9.183453842	0.199428225	1.676904155
AO	0.125000000	5.482693619	9.573850713	0.197858589	1.870158707
GWO	0.198318729	3.346429018	9.194711780	0.198821978	1.671023180
mESC	0.198856818	3.337056403	9.191457782	0.198856819	1.670306973

**Table 9:** Optimization results of stepping cone pulley.

Algorithms	Optimize Variables					Optimal Cost
	*C*_1_	*C*_2_	*C*_3_	*C*_4_	*C*_5_	
MPSO	60.00000000	60.00000000	90.000000000	90.000000000	84.485377267	1.843733 × 10^13^
CPO	42.484785315	51.840854067	81.259740380	88.646323832	89.499864989	7.621411 × 10^11^
AVOA	40.910417425	56.296556528	75.055850446	89.985907677	89.953762473	18.240889727
BWO	40.170645683	55.186639399	73.530759814	88.356141970	88.444647583	3.093407 × 10^8^
WOA	42.089877418	59.519146031	77.292737241	90.000000000	84.480369782	6.663866 × 10^10^
HHO	40.805285317	56.151766313	74.862844009	89.754721525	84.822379906	19.535605020
TSA	41.101782491	56.518690008	75.345851931	90.000000000	90.000000000	8.861758 × 10^8^
AO	40.924737487	56.308967580	75.087989757	90.000000000	89.951506033	2.468629 × 10^6^
GWO	40.877944163	56.248948471	74.994588668	89.908471215	87.356270007	3.104622 × 10^5^
mESC	40.550377897	55.800718370	74.394842053	89.194118489	85.245928684	16.983218316

**Table 10:** Optimization of robot gripping.

Algorithms	Optimize Variables							Optimal Cost
	*C*_1_	*C*_2_	*C*_3_	*C*_4_	*C*_5_	*C*_6_	*C*_7_	
MPSO	150.000000000	95.759902552	200.000000000	50.000000000	150.000000000	150.605188185	3.140000000	4.153373986
CPO	137.294003282	105.373760292	137.523639557	28.463975526	83.002354501	150.065561217	2.631685313	7.097144505
AVOA	149.706361538	100.150569369	176.072790271	49.183239911	104.041261757	108.521270616	2.535488368	3.617699429
BWO	126.906726514	106.159011456	164.707977178	19.928074374	118.764459960	117.190953565	2.541994213	4.557386267
WOA	149.999959796	122.837277782	161.536481625	21.759499781	120.981821722	164.597992373	2.811652016	4.782923603
HHO	149.899324642	142.921278615	158.704180512	0.005407940	82.922639703	176.846269350	2.297126605	4.705109515
TSA	150.000000000	146.228369030	186.244359892	3.518756036	32.108921190	111.374296080	1.771580385	3.028724915
AO	149.793012269	149.485909987	193.779795455	0.000000000	10.242012018	114.569361590	1.630962274	2.939182773
GWO	149.840044164	138.061632796	197.173819690	11.623821088	145.092241881	102.910952527	2.409118716	2.710770687
mESC	149.999999988	138.941007919	199.999999559	10.918737851	147.641681524	101.826637161	2.367787342	2.641270695

**Table 11:** Optimization of rolling element bearings.

Algorithms	Optimize Variables										Optimal Cost
	*C*_1_	*C*_2_	*C*_3_	*C*_4_	*C*_5_	*C*_6_	*C*_7_	*C*_8_	*C*_9_	*C*_10_	
MPSO	125.0000	31.5000	50.4900	0.6000	0.6000	0.5000	0.7000	0.4000	0.1000	0.8500	2.041186421 × 10^18^
CPO	126.7515	19.2516	6.2131	0.5919	0.5599	0.4940	0.6567	0.3366	0.0204	0.6263	22,969.355311515
AVOA	127.9200	18.0000	5.0272	0.6000	0.6000	0.4999	0.6998	0.3907	0.0652	0.6000	7012.353939168
BWO	129.1096	18.0251	4.5100	0.6000	0.6000	0.5000	0.7000	0.3000	0.0200	0.6000	17,038.621657089
WOA	129.9993	18.0000	4.5100	0.6000	0.5713	0.4024	0.7000	0.3000	0.0200	0.6000	17,302.364139520
HHO	131.1992	18.0000	4.5211	0.6000	0.6000	0.4879	0.6980	0.3000	0.0910	0.6000	16,958.215169212
TSA	130.3274	18.0096	4.5100	0.6000	0.6000	0.4386	0.6000	0.3000	0.0804	0.6000	16,990.260539260
AO	129.1367	18.0097	4.6452	0.6000	0.6000	0.4000	0.6189	0.3000	0.1000	0.6000	17,010.229234223
GWO	129.5493	18.0030	4.9183	0.6000	0.6000	0.4101	0.6358	0.3289	0.0459	0.6000	16,991.198492525
mESC	131.2000	18.0000	5.1877	0.6000	0.6000	0.4764	0.6246	0.3000	0.0793	0.6000	16,958.202286941

## Data Availability

All data generated or analyzed during the study are included in this published article.

## Supplementary Materials

The following supporting information can be downloaded at: https://www.mdpi.com/article/10.3390/biomimetics10040232/s1.
