# Supplementary material for: mESC: An Enhanced Escape Algorithm Fusing Multiple Strategies for Engineering Optimization
Source: Biomimetics (Basel). 2025 Apr 8;10(4):232. doi: 10.3390/biomimetics10040232 (PMC12025011; doi:10.3390/biomimetics10040232)
Supplement: Supplementary file 1 [file biomimetics-10-00232-s001.zip › biomimetics-3536543-supplementary.pdf]

Supplementary materials

Tables S1 and S2 show the parameter results of the worst individual proportion α in the 10 and 20 dimensions, respectively.
Tables S3 and S4 show the parameter results of the adaptive interference factor adjustment range β in the 10 and 20 dimensions, respectively.
Tables S5 and S6 show the results of mESC and the novel metaheuristic algorithm in 10 and 20 dimensions, respectively.
Tables S7 and S8 show the Wilcoxon test results of mESC and the novel metaheuristic algorithm in 10 and 20 dimensions, respectively.
Table S9 shows the running time of mESC and the new metaheuristic algorithm.
Tables S10 and S11 show the results of mESC and high-performance, winner algorithm in 10 and 20 dimensions, respectively.
Tables S12 and S13 show the Wilcoxon test results of mESC and high-performance, winner algorithm in 10 and 20 dimensions, respectively.
Table S14 shows the running time of mESC and high-performance, winner algorithms.

Figure S1 shows the convergence curves of mESC and the novel metaheuristic algorithm.
Figure S2 shows the boxplot of mESC and the novel metaheuristic algorithm.
Figure S3 shows the convergence curves of mESC and high-performance winner algorithm.
Figure S4 shows the boxplot of mESC and high-performance, winner algorithm.

Table S1. Experimental results of the worst individual proportion α on CEC2022 (10 dimensions).

F	$\alpha = 0.05$	$\alpha = 0.10$	$\alpha = 0.15$	$\alpha = 0.20$
F1	3.0000×10^2	3.0000×10^2	3.0000×10^2	3.0000×10^2
F2	4.0402×10^2	4.0419×10^2	4.0426×10^2	4.0480×10^2
F3	6.0000×10^2	6.0000×10^2	6.0000×10^2	6.0000×10^2
F4	8.0879×10^2	8.0935×10^2	8.1141×10^2	8.1015×10^2
F5	9.0000×10^2	9.0000×10^2	9.0000×10^2	9.0000×10^2
F6	2.7134×10^3	2.7983×10^3	2.7957×10^3	2.8588×10^3
F7	2.0034×10^3	2.0084×10^3	2.0080×10^3	2.0078×10^3
F8	2.2105×10^3	2.2145×10^3	2.2155×10^3	2.2183×10^3
F9	2.5226×10^3	2.5225×10^3	2.5191×10^3	2.5206×10^3
F10	2.5329×10^3	2.5231×10^3	2.5258×10^3	2.5364×10^3
F11	2.6133×10^3	2.6183×10^3	2.6467×10^3	2.6550×10^3
F12	2.8634×10^3	2.8633×10^3	2.8627×10^3	2.8637×10^3
Rank	1	2	3	4

Table S2. Experimental results of the worst individual proportion α on CEC2022 (20 dimensions).

F	$\alpha = 0.05$	$\alpha = 0.10$	$\alpha = 0.15$	$\alpha = 0.20$
F1	7.9158×10^2	7.1897×10^2	5.4704×10^2	1.2743×10^3
F2	4.5114×10^2	4.5111×10^2	4.5492×10^2	4.5136×10^2
F3	6.0000×10^2	6.0000×10^2	6.0000×10^2	6.0000×10^2
F4	8.2341×10^2	8.2757×10^2	8.2205×10^2	8.2189×10^2
F5	9.0002×10^2	9.0006×10^2	9.0004×10^2	9.0004×10^2
F6	3.4410×10^3	4.1868×10^3	4.5614×10^3	4.4593×10^3
F7	2.0265×10^3	2.0311×10^3	2.0343×10^3	2.0278×10^3

F8	2.2256×10^3	2.2213×10^3	2.2214×10^3	2.2235×10^3
F9	2.4806×10^3	2.4803×10^3	2.4802×10^3	2.4809×10^3
F10	2.5174×10^3	2.5364×10^3	2.5412×10^3	2.5112×10^3
F11	2.9033×10^3	2.9133×10^3	2.9067×10^3	2.9172×10^3
F12	2.9440×10^3	2.9439×10^3	2.9470×10^3	2.9433×10^3
Rank	1	2	3	4

Table S3 Experimental results of adaptive disturbance factor adjustment range β on CEC2022 (10 dimensions).

**F**	β: **Reduce from 0.6 to 0.5**	β: **Reduce from 0.7 to 0.5**	β: **Reduce from 0.8 to 0.5**	β: **Reduce from 0.9 to 0.5**
F1	3.0000×10^2	3.0000×10^2	3.0000×10^2	3.0000×10^2
F2	4.0377×10^2	4.0382×10^2	4.0386×10^2	4.0459×10^2
F3	6.0000×10^2	6.0000×10^2	6.0000×10^2	6.0000×10^2
F4	8.0866×10^2	8.1012×10^2	8.0793×10^2	8.0929×10^2
F5	9.0000×10^2	9.0000×10^2	9.0000×10^2	9.0000×10^2
F6	2.5496×10^3	2.4698×10^3	2.6857×10^3	2.6798×10^3
F7	2.0070×10^3	2.0059×10^3	2.0049×10^3	2.0067×10^3
F8	2.2164×10^3	2.2107×10^3	2.2141×10^3	2.2127×10^3
F9	2.5170×10^3	2.5190×10^3	2.5235×10^3	2.5258×10^3
F10	2.5443×10^3	2.5334×10^3	2.5217×10^3	2.5471×10^3
F11	2.6400×10^3	2.6533×10^3	2.6733×10^3	2.6717×10^3
F12	2.8632×10^3	2.8640×10^3	2.8636×10^3	2.8635×10^3
Rank	2	1	3	4

Table S4 Experimental results of adaptive disturbance factor adjustment range β on CEC2022 (20 dimensions).

**F**	β: **Reduce from 0.6 to 0.5**	β: **Reduce from 0.7 to 0.5**	β: **Reduce from 0.8 to 0.5**	β: **Reduce from 0.9 to 0.5**
F1	1.2404×10^3	8.9801×10^2	1.4680×10^3	1.3517×10^3
F2	4.5412×10^2	4.5018×10^2	4.5061×10^2	4.5248×10^2
F3	6.0000×10^2	6.0000×10^2	6.0003×10^2	6.0000×10^2
F4	8.2199×10^2	8.2412×10^2	8.2269×10^2	8.2361×10^2
F5	9.0010×10^2	9.0004×10^2	9.0005×10^2	9.0004×10^2
F6	4.6845×10^3	3.5103×10^3	4.2193×10^3	4.9078×10^3
F7	2.0334×10^3	2.0305×10^3	2.0318×10^3	2.0308×10^3
F8	2.2217×10^3	2.2219×10^3	2.2257×10^3	2.2336×10^3
F9	2.4811×10^3	2.4804×10^3	2.4807×10^3	2.4804×10^3
F10	2.5278×10^3	2.5113×10^3	2.5305×10^3	2.5240×10^3
F11	2.9067×10^3	2.9167×10^3	2.9100×10^3	2.9033×10^3
F12	2.9482×10^3	2.9436×10^3	2.9473×10^3	2.9498×10^3
Rank	4	1	3	2

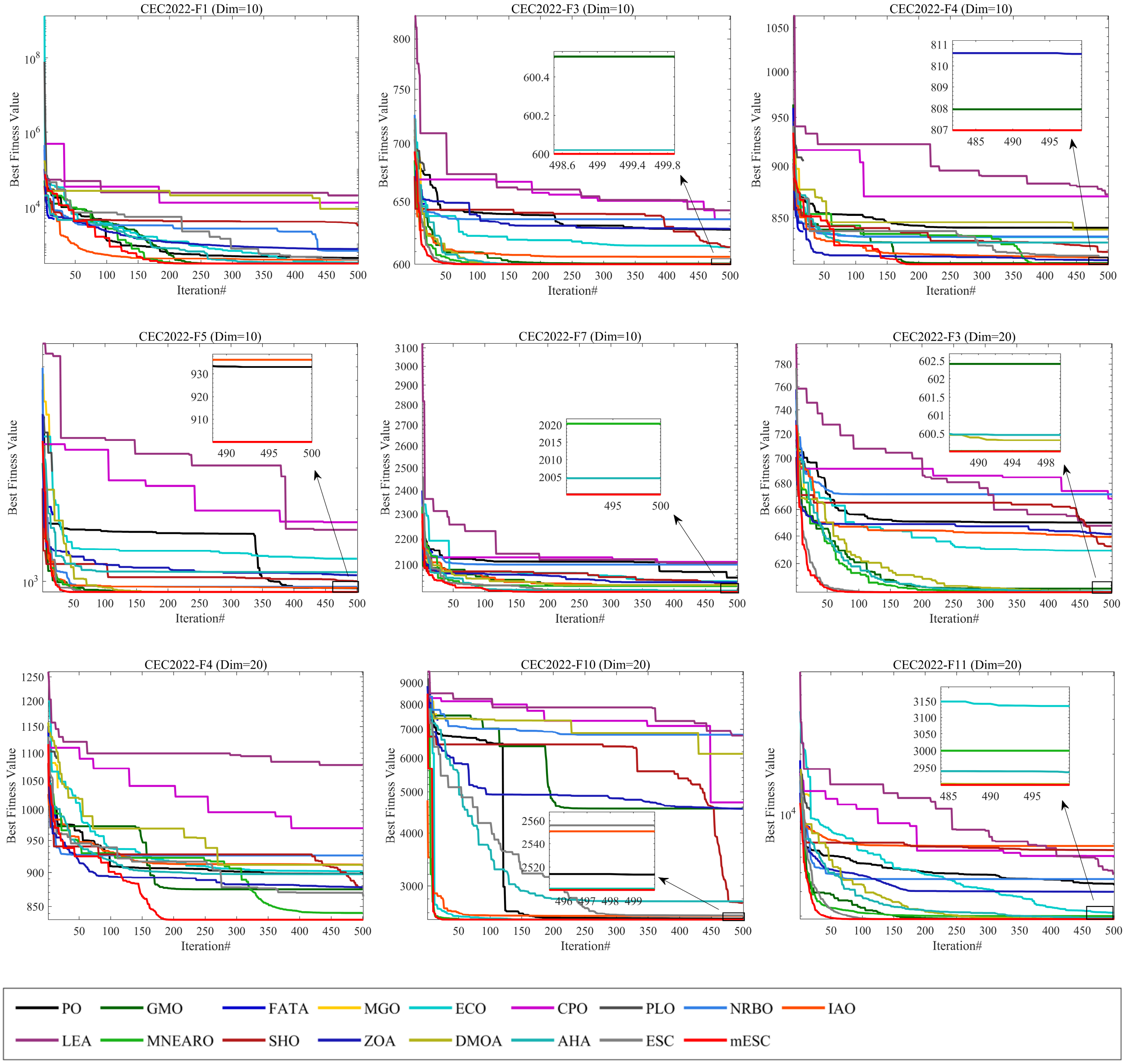

Figure S1. Convergence curves of mESC and novel metaheuristic algorithms on CEC2022.

Table S5. The Results of mESC and New Meta Heuristic Algorithm on CEC2022 (10 Dimensions).

F	Index	PO	GMO	FATA	MGO	ECO	CPO	PLO	NRBO	IAO	LEA	MNEARO	SHO	ZOA	DMOA	AHA	ESC	mESC
1	Best	3.1503×10^{2}	3.0000×10^{2}	4.0353×10^{3}	8.8588×10^{3}	3.0591×10^{2}	3.3069×10^{3}	8.8177×10^{3}	5.0289×10^{2}	3.0000×10^{2}	7.0720×10^{3}	3.0000×10^{2}	6.6098×10^{2}	3.8952×10^{2}	1.6856×10^{3}	3.0220×10^{2}	3.1568×10^{2}	3.0000×10^{2}
	Mean	4.1682×10^{2}	3.0003×10^{2}	1.2704×10^{4}	2.3874×10^{4}	1.2758×10^{3}	1.5928×10^{4}	1.9684×10^{4}	1.2336×10^{3}	6.5838×10^{2}	2.5187×10^{4}	3.0000×10^{2}	3.6372×10^{3}	1.8971×10^{3}	8.1830×10^{3}	4.0934×10^{2}	4.4346×10^{2}	**3.0000×10^{2}**
	Std	1.0985×10^{2}	5.5381×10^{-2}	1.2564×10^{4}	1.0122×10^{4}	1.1174×10^{3}	6.3824×10^{3}	6.9269×10^{3}	7.5230×10^{2}	8.6520×10^{2}	1.0348×10^{4}	4.3638×10^{-7}	2.4793×10^{3}	2.4265×10^{3}	5.2468×10^{3}	1.9620×10^{2}	1.0526×10^{2}	7.4639×10-14
	Rank	5	3	13	16	8	14	15	10	6	17	2	11	9	12	4	7	1
2	Best	4.0184×10^{2}	4.0000×10^{2}	4.7169×10^{2}	6.6677×10^{2}	4.0013×10^{2}	4.7029×10^{2}	6.9584×10^{2}	4.1099×10^{2}	4.0004×10^{2}	4.2457×10^{2}	4.0000×10^{2}	4.0040×10^{2}	4.0067×10^{2}	4.0286×10^{2}	4.0000×10^{2}	4.0000×10^{2}	4.0000×10^{2}
	Mean	4.3707×10^{2}	**4.0256×10^{2}**	6.6650×10^{2}	1.2026×10^{3}	4.0630×10^{2}	6.8796×10^{2}	1.2908×10^{3}	4.5684×10^{2}	4.1217×10^{2}	4.8368×10^{2}	4.0765×10^{2}	4.7959×10^{2}	4.5207×10^{2}	4.0715×10^{2}	4.1532×10^{2}	4.1054×10^{2}	4.0327×10^{2}
	Std	3.2534×10^{1}	1.1260×10^{1}	1.6417×10^{2}	3.4312×10^{2}	3.4094×10^{0}	1.0483×10^{2}	3.7090×10^{2}	4.2310×10^{1}	2.1521×10^{1}	3.8796×10^{1}	1.2280×10^{1}	7.0082×10^{1}	3.3987×10^{1}	1.3650×10^{0}	2.7776×10^{1}	1.5291×10^{1}	3.9538×10^{0}
	Rank	9	1	14	16	8	15	17	12	5	13	4	11	10	6	3	7	2
3	Best	6.0926×10^{2}	6.0000×10^{2}	6.2180×10^{2}	6.4492×10^{2}	6.0054×10^{2}	6.2592×10^{2}	6.3265×10^{2}	6.1049×10^{2}	6.0309×10^{2}	6.1644×10^{2}	6.0000×10^{2}	6.0616×10^{2}	6.0720×10^{2}	6.0000×10^{2}	6.0000×10^{2}	6.0000×10^{2}	6.0000×10^{2}
	Mean	6.2222×10^{2}	6.0086×10^{2}	6.5155×10^{2}	6.6545×10^{2}	6.1930×10^{2}	6.4078×10^{2}	6.5783×10^{2}	6.2588×10^{2}	6.1538×10^{2}	6.3984×10^{2}	6.0000×10^{2}	6.1812×10^{2}	6.1955×10^{2}	6.0000×10^{2}	6.0073×10^{2}	6.0000×10^{2}	**6.0000×10^{2}**
	Std	9.2165×10^{0}	1.0189×10^{0}	1.5234×10^{1}	9.4925×10^{0}	1.1144×10^{1}	7.7820×10^{0}	1.3000×10^{1}	9.4758×10^{0}	1.0367×10^{1}	1.5285×10^{1}	3.1227×10^{-4}	9.0965×10^{0}	8.3180×10^{0}	8.5344×10^{-6}	2.0685×10^{0}	3.5085×10^{-5}	2.7193×10^{-5}
	Rank	11	6	15	17	10	14	16	12	7	13	4	8	9	2	5	3	1
4	Best	8.1664×10^{2}	8.0398×10^{2}	8.3223×10^{2}	8.6199×10^{2}	8.0797×10^{2}	8.5139×10^{2}	8.6900×10^{2}	8.1913×10^{2}	8.0493×10^{2}	8.4954×10^{2}	8.0398×10^{2}	8.1496×10^{2}	8.0797×10^{2}	8.2072×10^{2}	8.0895×10^{2}	8.0381×10^{2}	8.0199×10^{2}
	Mean	8.2593×10^{2}	8.1579×10^{2}	8.5222×10^{2}	8.8996×10^{2}	8.2218×10^{2}	8.7161×10^{2}	8.8943×10^{2}	8.3406×10^{2}	8.1464×10^{2}	8.6939×10^{2}	**8.0899×10^{2}**	8.2498×10^{2}	8.1716×10^{2}	8.3530×10^{2}	8.2381×10^{2}	8.1251×10^{2}	8.0925×10^{2}
	Std	6.0540×10^{0}	1.0007×10^{1}	1.0782×10^{1}	1.4424×10^{1}	7.7755×10^{0}	9.1970×10^{0}	1.0804×10^{1}	7.9021×10^{0}	5.8674×10^{0}	1.2140×10^{1}	3.4582×10^{0}	6.5607×10^{0}	5.7709×10^{0}	5.8556×10^{0}	7.7463×10^{0}	3.8384×10^{0}	4.2225×10^{0}
	Rank	9	5	13	16	7	15	17	11	4	14	1	10	6	12	8	3	2
5	Best	9.3562×10^{2}	9.0000×10^{2}	1.2198×10^{3}	1.6405×10^{3}	9.4145×10^{2}	1.0179×10^{3}	1.6152×10^{3}	9.2340×10^{2}	9.0009×10^{2}	1.1865×10^{3}	9.0000×10^{2}	9.3933×10^{2}	9.0103×10^{2}	9.0000×10^{2}	9.0000×10^{2}	9.0000×10^{2}	9.0000×10^{2}
	Mean	1.1454×10^{3}	9.0001×10^{2}	1.5744×10^{3}	2.7256×10^{3}	1.0833×10^{3}	1.4766×10^{3}	2.7814×10^{3}	1.0682×10^{3}	9.7333×10^{2}	2.0422×10^{3}	9.0003×10^{2}	1.1105×10^{3}	1.0543×10^{3}	9.0000×10^{2}	9.1584×10^{2}	9.0001×10^{2}	**9.0000×10^{2}**
	Std	1.8038×10^{2}	2.7316×10^{-2}	1.9874×10^{2}	5.6453×10^{2}	1.1932×10^{2}	2.5593×10^{2}	5.9870×10^{2}	9.1314×10^{1}	9.7240×10^{1}	6.5800×10^{2}	1.1555×10^{-1}	1.5345×10^{2}	7.4513×10^{1}	1.9117×10^{-5}	2.2442×10^{1}	3.3819×10^{-2}	3.8223×10^{-8}
	Rank	12	5	14	17	8	13	16	9	7	15	2	11	10	3	6	4	1
6	Best	2.1563×10^{3}	1.8874×10^{3}	1.2660×10^{4}	$6.4153\times10^{+06}$	1.9060×10^{3}	$1.0901\times10^{+06}$	$3.1984\times10^{+06}$	1.9048×10^{3}	1.8005×10^{3}	5.2266×10^{4}	1.8001×10^{3}	1.9068×10^{3}	1.8736×10^{3}	6.4304×10^{3}	1.8070×10^{3}	1.8299×10^{3}	1.8139×10^{3}
	Mean	4.9852×10^{3}	2.4633×10^{3}	$4.0865\times10^{+05}$	$1.6504\times10^{+08}$	4.3913×10^{3}	$2.5978\times10^{+07}$	$1.9124\times10^{+08}$	4.1287×10^{3}	1.8117×10^{3}	$3.5124\times10^{+06}$	**1.8011×10^{3}**	4.9251×10^{3}	3.2108×10^{3}	8.9406×10^{4}	2.4966×10^{3}	3.3765×10^{3}	2.5430×10^{3}
	Std	2.3354×10^{3}	5.8865×10^{2}	$4.1287\times10^{+05}$	$1.3868\times10^{+08}$	1.8827×10^{3}	$2.5111\times10^{+07}$	$2.1555\times10^{+08}$	2.1311×10^{3}	1.4936×10^{1}	$3.2197\times10^{+06}$	1.0266×10^{0}	1.8255×10^{3}	1.1471×10^{3}	8.4744×10^{4}	1.1845×10^{3}	1.5615×10^{3}	1.0503×10^{3}
	Rank	10	5	13	16	9	15	17	8	2	14	1	11	6	12	3	7	4
7	Best	2.0241×10^{3}	2.0090×10^{3}	2.0534×10^{3}	2.0406×10^{3}	2.0216×10^{3}	2.0591×10^{3}	2.0774×10^{3}	2.0349×10^{3}	2.0063×10^{3}	2.0426×10^{3}	2.0000×10^{3}	2.0199×10^{3}	2.0127×10^{3}	2.0226×10^{3}	2.0000×10^{3}	2.0000×10^{3}	2.0000×10^{3}
	Mean	2.0546×10^{3}	2.0402×10^{3}	2.1105×10^{3}	2.1230×10^{3}	2.0431×10^{3}	2.1072×10^{3}	2.1406×10^{3}	2.0617×10^{3}	2.0257×10^{3}	2.0940×10^{3}	2.0048×10^{3}	2.0401×10^{3}	2.0499×10^{3}	2.0265×10^{3}	2.0147×10^{3}	2.0065×10^{3}	**2.0026×10^{3}**
	Std	2.1120×10^{1}	1.6100×10^{1}	2.9090×10^{1}	2.9903×10^{1}	2.3177×10^{1}	2.4347×10^{1}	3.5669×10^{1}	2.5190×10^{1}	7.8863×10^{0}	2.8690×10^{1}	8.0190×10^{0}	1.2635×10^{1}	1.6915×10^{1}	2.0690×10^{0}	8.7722×10^{0}	9.1379×10^{0}	6.0193×10^{0}

	Rank	11	7	15	16	8	14	17	12	5	13	2	9	10	6	4	3	1
8	Best	2.2237×10^{3}	2.2229×10^{3}	2.2272×10^{3}	2.2380×10^{3}	2.2053×10^{3}	2.2306×10^{3}	2.2333×10^{3}	2.2276×10^{3}	2.2051×10^{3}	2.2307×10^{3}	2.2002×10^{3}	2.2105×10^{3}	2.2088×10^{3}	2.2256×10^{3}	2.2009×10^{3}	2.2005×10^{3}	2.2001×10^{3}
	Mean	2.2295×10^{3}	2.2309×10^{3}	2.2783×10^{3}	2.2686×10^{3}	2.2263×10^{3}	2.2495×10^{3}	2.2876×10^{3}	2.2542×10^{3}	2.2175×10^{3}	2.2572×10^{3}	**2.2091×10^{3}**	2.2248×10^{3}	2.2466×10^{3}	2.2313×10^{3}	2.2199×10^{3}	2.2171×10^{3}	2.2132×10^{3}
	Std	4.6817×10^{0}	2.1107×10^{1}	5.4924×10^{1}	3.0749×10^{1}	5.3518×10^{0}	1.2101×10^{1}	5.0397×10^{1}	4.7363×10^{1}	6.5673×10^{0}	2.5173×10^{1}	9.3658×10^{0}	3.4687×10^{0}	5.3262×10^{1}	2.5399×10^{0}	3.7349×10^{0}	7.7215×10^{0}	9.3043×10^{0}
	Rank	9	8	13	16	7	14	17	12	5	15	1	6	10	11	3	4	2
9	Best	2.5294×10^{3}	2.5293×10^{3}	2.6219×10^{3}	2.6192×10^{3}	2.5293×10^{3}	2.6063×10^{3}	2.6721×10^{3}	2.5304×10^{3}	2.5293×10^{3}	2.5464×10^{3}	2.5293×10^{3}	2.5695×10^{3}	2.5616×10^{3}	2.4855×10^{3}	2.5293×10^{3}	2.5293×10^{3}	2.5001×10^{3}
	Mean	2.5827×10^{3}	2.5301×10^{3}	2.6938×10^{3}	2.7421×10^{3}	2.5436×10^{3}	2.6797×10^{3}	2.7773×10^{3}	2.5821×10^{3}	2.5295×10^{3}	2.6473×10^{3}	2.5293×10^{3}	2.6044×10^{3}	2.6156×10^{3}	**2.4856×10^{3}**	2.5296×10^{3}	2.5293×10^{3}	2.5224×10^{3}
	Std	3.6835×10^{1}	1.2625×10^{0}	4.0858×10^{1}	5.9418×10^{1}	3.7993×10^{1}	3.8100×10^{1}	9.1769×10^{1}	4.3044×10^{1}	9.2789×10^{-1}	4.6039×10^{1}	1.1942×10-13	3.2403×10^{1}	3.1854×10^{1}	1.0270×10^{-1}	1.2571×10^{0}	2.1716×10^{-8}	1.0048×10^{1}
	Rank	10	8	15	16	7	14	17	9	5	13	3	11	12	1	6	4	2
10	Best	2.5004×10^{3}	2.5002×10^{3}	2.5013×10^{3}	2.5197×10^{3}	2.5003×10^{3}	2.5074×10^{3}	2.5200×10^{3}	2.5003×10^{3}	2.5003×10^{3}	2.5009×10^{3}	2.5002×10^{3}	2.5010×10^{3}	2.5004×10^{3}	2.5004×10^{3}	2.5002×10^{3}	2.5003×10^{3}	2.5001×10^{3}
	Mean	2.5457×10^{3}	2.5736×10^{3}	2.6198×10^{3}	2.7290×10^{3}	2.6043×10^{3}	2.5575×10^{3}	2.7311×10^{3}	2.5392×10^{3}	2.5307×10^{3}	2.5481×10^{3}	**2.5074×10^{3}**	2.5874×10^{3}	2.5649×10^{3}	2.5065×10^{3}	2.5236×10^{3}	2.5225×10^{3}	2.5366×10^{3}
	Std	6.2546×10^{1}	5.7459×10^{1}	1.0335×10^{2}	1.8561×10^{2}	1.1130×10^{2}	8.4774×10^{1}	1.9763×10^{2}	6.4421×10^{1}	5.0931×10^{1}	8.3632×10^{1}	2.6978×10^{1}	5.5923×10^{1}	7.0221×10^{1}	2.1154×10^{1}	4.6895×10^{1}	4.5126×10^{1}	5.2367×10^{1}
	Rank	9	8	15	17	12	13	16	7	5	10	1	14	11	6	4	2	3
11	Best	2.6121×10^{3}	2.6000×10^{3}	2.7666×10^{3}	3.0738×10^{3}	2.6007×10^{3}	2.8042×10^{3}	2.8740×10^{3}	2.7495×10^{3}	2.6183×10^{3}	2.7587×10^{3}	2.6000×10^{3}	2.6168×10^{3}	2.6109×10^{3}	2.6000×10^{3}	2.6000×10^{3}	2.6000×10^{3}	2.6000×10^{3}
	Mean	2.7327×10^{3}	2.7033×10^{3}	3.4601×10^{3}	3.6427×10^{3}	2.6892×10^{3}	3.0058×10^{3}	3.5714×10^{3}	2.9041×10^{3}	2.7147×10^{3}	2.9344×10^{3}	2.6251×10^{3}	2.8302×10^{3}	2.8993×10^{3}	2.6681×10^{3}	2.6058×10^{3}	2.6752×10^{3}	**2.6250×10^{3}**
	Std	1.0579×10^{2}	1.4967×10^{2}	4.2105×10^{2}	4.4480×10^{2}	9.2111×10^{1}	1.7952×10^{2}	4.8109×10^{2}	1.9673×10^{2}	9.4510×10^{1}	2.3316×10^{2}	5.7014×10^{1}	1.7604×10^{2}	2.1469×10^{2}	7.0887×10^{1}	2.7421×10^{1}	8.6039×10^{1}	7.9623×10^{1}
	Rank	9	6	15	17	7	14	16	12	8	13	2	10	11	5	3	4	1
12	Best	2.8626×10^{3}	2.8630×10^{3}	2.8962×10^{3}	2.9247×10^{3}	2.8623×10^{3}	2.9019×10^{3}	2.8760×10^{3}	2.8636×10^{3}	2.8596×10^{3}	2.8659×10^{3}	2.8586×10^{3}	2.8701×10^{3}	2.9001×10^{3}	2.8908×10^{3}	2.8644×10^{3}	2.8594×10^{3}	2.8563×10^{3}
	Mean	2.8685×10^{3}	2.8657×10^{3}	2.9443×10^{3}	2.9970×10^{3}	2.8732×10^{3}	2.9644×10^{3}	2.9616×10^{3}	2.8678×10^{3}	2.8642×10^{3}	2.8822×10^{3}	**2.8626×10^{3}**	2.8999×10^{3}	2.9394×10^{3}	2.8995×10^{3}	2.8699×10^{3}	2.8646×10^{3}	2.8635×10^{3}
	Std	6.7833×10^{0}	1.8977×10^{0}	4.1631×10^{1}	5.7052×10^{1}	2.1390×10^{1}	3.7174×10^{1}	3.8674×10^{1}	4.1222×10^{0}	3.0518×10^{0}	2.4610×10^{1}	1.6001×10^{0}	1.8228×10^{1}	2.9799×10^{1}	1.9688×10^{0}	8.9927×10^{0}	1.4386×10^{0}	2.1093×10^{0}
	Rank	6	5	14	17	7	16	15	8	3	10	1	11	13	12	9	4	2
Mean rank		9.17	5.58	14.08	16.42	8.17	14.25	16.33	10.17	5.17	13.33	2.00	10.25	9.75	7.33	4.83	4.33	1.83
Result		9	6	14	17	8	15	16	11	5	13	2	12	10	7	4	3	**1**

Table S6. The Results of mESC and New Meta Heuristic Algorithm on CEC2022 (20 Dimensions).

F	Index	PO	GMO	FATA	MGO	ECO	CPO	PLO	NRBO	IAO	LEA	MNEARO	SHO	ZOA	DMOA	AHA	ESC	mESC
1	Best	4.3518×10^{4}	2.6748×10^{4}	6.7072×10^{3}	3.6924×10^{4}	4.6343×10^{4}	7.7079×10^{3}	4.2471×10^{3}	4.0403×10^{4}	3.6484×10^{2}	1.3566×10^{4}	8.0029×10^{3}	6.8296×10^{4}	4.2744×10^{3}	8.7182×10^{3}	3.0289×10^{2}	4.3518×10^{4}	2.6748×10^{4}
	Mean	9.4672×10^{4}	8.3430×10^{4}	1.8946×10^{4}	5.9138×10^{4}	6.8846×10^{4}	1.5350×10^{4}	1.2670×10^{4}	8.5085×10^{4}	9.3920×10^{2}	1.8011×10^{4}	1.5802×10^{4}	1.2041×10^{5}	1.1065×10^{4}	1.6987×10^{4}	1.1203×10^{3}	9.4672×10^{4}	**8.3430×10^{4}**
	Std	3.3479×10^{4}	3.2270×10^{4}	8.6978×10^{3}	1.6950×10^{4}	1.2193×10^{4}	4.1620×10^{3}	4.5130×10^{3}	2.8591×10^{4}	4.2493×10^{2}	3.5644×10^{3}	4.1642×10^{3}	3.0764×10^{4}	5.4634×10^{3}	4.4361×10^{3}	1.1229×10^{3}	3.3479×10^{4}	3.2270×10^{4}

	Rank	6	5	16	14	10	12	13	7	4	15	2	11	8	17	3	9	1
2	Best	7.5511×10^{2}	1.7773×10^{3}	4.5789×10^{2}	9.8748×10^{2}	1.5838×10^{3}	5.5632×10^{2}	7.9686×10^{2}	6.8125×10^{2}	4.2909×10^{2}	5.4000×10^{2}	4.9250×10^{2}	4.1732×10^{2}	4.3274×10^{2}	4.3188×10^{2}	4.3410×10^{2}	7.5511×10^{2}	1.7773×10^{3}
	Mean	1.5166×10^{3}	4.7969×10^{3}	5.1520×10^{2}	1.5504×10^{3}	4.4472×10^{3}	7.2820×10^{2}	1.1628×10^{3}	1.0224×10^{3}	4.5242×10^{2}	7.6568×10^{2}	6.7654×10^{2}	4.2176×10^{2}	4.8708×10^{2}	**4.6109×10^{2}**	4.4746×10^{2}	1.5166×10^{3}	4.7969×10^{3}
	Std	4.4449×10^{2}	1.8930×10^{3}	5.0452×10^{1}	3.1449×10^{2}	1.4350×10^{3}	1.1597×10^{2}	3.2172×10^{2}	3.1452×10^{2}	9.9588×10^{0}	1.4329×10^{2}	1.0380×10^{2}	1.2411×10^{1}	4.5061×10^{1}	1.7122×10^{1}	3.6990×10^{0}	4.4449×10^{2}	1.8930×10^{3}
	Rank	8	4	14	16	7	15	17	10	13	12	3	11	9	1	6	5	2
3	Best	6.6303×10^{2}	6.7111×10^{2}	6.1737×10^{2}	6.5257×10^{2}	6.6807×10^{2}	6.2460×10^{2}	6.2483×10^{2}	6.4369×10^{2}	6.0000×10^{2}	6.2671×10^{2}	6.3348×10^{2}	6.0023×10^{2}	6.0050×10^{2}	6.0000×10^{2}	6.0000×10^{2}	6.6303×10^{2}	6.7111×10^{2}
	Mean	6.8144×10^{2}	6.9693×10^{2}	6.4382×10^{2}	6.7205×10^{2}	6.9652×10^{2}	6.5400×10^{2}	6.5181×10^{2}	6.8243×10^{2}	6.0008×10^{2}	6.4373×10^{2}	6.4751×10^{2}	6.0056×10^{2}	6.0563×10^{2}	6.0000×10^{2}	6.0001×10^{2}	6.8144×10^{2}	**6.9693×10^{2}**
	Std	1.0102×10^{1}	1.2311×10^{1}	1.2921×10^{1}	9.2079×10^{0}	1.3522×10^{1}	1.2620×10^{1}	9.8998×10^{0}	1.6978×10^{1}	2.0314×10^{-1}	9.0202×10^{0}	7.8656×10^{0}	2.3992×10^{-1}	7.0455×10^{0}	9.6302×10^{-3}	4.6591×10^{-2}	1.0102×10^{1}	1.2311×10^{1}
	Rank	11	6	15	16	8	13	17	12	10	14	3	7	9	4	5	2	1
4	Best	9.2993×10^{2}	9.9375×10^{2}	8.3336×10^{2}	9.5388×10^{2}	9.7050×10^{2}	8.8379×10^{2}	8.7782×10^{2}	9.5228×10^{2}	8.1736×10^{2}	8.7243×10^{2}	8.4189×10^{2}	9.1474×10^{2}	8.4978×10^{2}	8.3979×10^{2}	8.0796×10^{2}	9.2993×10^{2}	9.9375×10^{2}
	Mean	9.6540×10^{2}	1.0519×10^{3}	8.7638×10^{2}	9.9960×10^{2}	1.0510×10^{3}	9.3635×10^{2}	9.0152×10^{2}	1.0139×10^{3}	8.3901×10^{2}	8.9763×10^{2}	8.7441×10^{2}	9.3299×10^{2}	8.7570×10^{2}	8.6393×10^{2}	8.2096×10^{2}	9.6540×10^{2}	**1.0519×10^{3}**
	Std	1.5690×10^{1}	2.7466×10^{1}	1.9846×10^{1}	2.0001×10^{1}	2.9728×10^{1}	1.8204×10^{1}	1.2413×10^{1}	2.7318×10^{1}	2.3148×10^{1}	1.6197×10^{1}	1.7221×10^{1}	9.0999×10^{0}	1.2337×10^{1}	9.6250×10^{0}	8.8604×10^{0}	1.5690×10^{1}	2.7466×10^{1}
	Rank	10	3	13	16	7	14	17	12	9	15	2	8	5	11	6	4	1
5	Best	2.9093×10^{3}	4.3404×10^{3}	1.7613×10^{3}	2.5956×10^{3}	6.1195×10^{3}	1.2843×10^{3}	1.3292×10^{3}	4.0748×10^{3}	9.0110×10^{2}	1.7684×10^{3}	1.4059×10^{3}	9.0946×10^{2}	1.2154×10^{3}	9.0000×10^{2}	9.0000×10^{2}	2.9093×10^{3}	4.3404×10^{3}
	Mean	4.0728×10^{3}	9.4073×10^{3}	2.3481×10^{3}	4.3046×10^{3}	9.5246×10^{3}	2.5783×10^{3}	2.1895×10^{3}	7.9374×10^{3}	9.3055×10^{2}	2.3449×10^{3}	1.9526×10^{3}	1.0456×10^{3}	2.0582×10^{3}	9.0108×10^{2}	9.0004×10^{2}	4.0728×10^{3}	**9.4073×10^{3}**
	Std	5.6429×10^{2}	1.6920×10^{3}	3.4820×10^{2}	7.2270×10^{2}	1.8082×10^{3}	5.6225×10^{2}	3.3585×10^{2}	2.2249×10^{3}	6.5281×10^{1}	2.6832×10^{2}	2.1424×10^{2}	1.3736×10^{2}	4.2015×10^{2}	1.7313×10^{0}	1.2758×10^{-1}	5.6429×10^{2}	1.6920×10^{3}
	Rank	11	3	13	17	10	14	16	12	8	15	4	9	6	5	7	2	1
6	Best	1.8550×10^{7}	8.9951×10^{8}	2.3311×10^{3}	1.8629×10^{8}	4.4885×10^{8}	6.9997×10^{5}	1.9091×10^{3}	2.5741×10^{7}	1.8299×10^{3}	4.5180×10^{4}	5.8660×10^{3}	3.6685×10^{6}	1.8842×10^{3}	1.9223×10^{3}	1.8771×10^{3}	1.8550×10^{7}	8.9951×10^{8}
	Mean	4.5286×10^{8}	3.7002×10^{9}	3.3434×10^{4}	8.5210×10^{8}	2.6441×10^{9}	2.3580×10^{7}	2.9713×10^{3}	1.8833×10^{8}	**5.5203×10^{3}**	1.5222×10^{7}	2.0992×10^{7}	2.2722×10^{7}	3.4659×10^{3}	4.3374×10^{3}	3.9379×10^{3}	4.5286×10^{8}	3.7002×10^{9}
	Std	5.1203×10^{8}	1.5822×10^{9}	1.1533×10^{5}	4.1799×10^{8}	1.3687×10^{9}	2.0516×10^{7}	2.0041×10^{3}	1.1294×10^{8}	6.5022×10^{3}	3.7615×10^{7}	3.4521×10^{7}	1.7377×10^{7}	1.9141×10^{3}	3.1311×10^{3}	2.2453×10^{3}	5.1203×10^{8}	1.5822×10^{9}
	Rank	8	6	14	17	7	15	16	11	1	13	2	9	10	12	3	5	4
7	Best	2.1638×10^{3}	2.2103×10^{3}	2.0647×10^{3}	2.1721×10^{3}	2.1958×10^{3}	2.1130×10^{3}	2.0408×10^{3}	2.1076×10^{3}	2.0040×10^{3}	2.0799×10^{3}	2.0685×10^{3}	2.1002×10^{3}	2.0221×10^{3}	2.0155×10^{3}	2.0054×10^{3}	2.1638×10^{3}	2.2103×10^{3}
	Mean	2.3188×10^{3}	2.3306×10^{3}	2.1446×10^{3}	2.2759×10^{3}	2.3087×10^{3}	2.1827×10^{3}	2.0925×10^{3}	2.2710×10^{3}	2.0262×10^{3}	2.1427×10^{3}	**2.1286×10^{3}**	2.1386×10^{3}	2.0710×10^{3}	2.0370×10^{3}	2.0373×10^{3}	2.3188×10^{3}	2.3306×10^{3}
	Std	1.0983×10^{2}	7.9415×10^{1}	4.6221×10^{1}	6.5132×10^{1}	5.5329×10^{1}	5.2521×10^{1}	2.4657×10^{1}	7.1893×10^{1}	1.0527×10^{1}	3.8144×10^{1}	2.8188×10^{1}	2.2770×10^{1}	5.3142×10^{1}	9.1624×10^{0}	2.9555×10^{1}	1.0983×10^{2}	7.9415×10^{1}
	Rank	11	6	15	17	9	13	16	12	5	14	1	8	7	10	4	3	2
8	Best	2.2373×10^{3}	2.3054×10^{3}	2.2271×10^{3}	2.2772×10^{3}	2.3219×10^{3}	2.2320×10^{3}	2.2251×10^{3}	2.2683×10^{3}	2.2220×10^{3}	2.2283×10^{3}	2.2275×10^{3}	2.2469×10^{3}	2.2207×10^{3}	2.2264×10^{3}	2.2204×10^{3}	2.2373×10^{3}	2.3054×10^{3}
	Mean	2.3458×10^{3}	2.8441×10^{3}	2.2844×10^{3}	2.4296×10^{3}	2.7302×10^{3}	2.3180×10^{3}	2.2308×10^{3}	2.4841×10^{3}	2.2283×10^{3}	2.2924×10^{3}	2.3136×10^{3}	2.3034×10^{3}	2.2242×10^{3}	2.2305×10^{3}	2.2218×10^{3}	2.3458×10^{3}	**2.8441×10^{3}**
	Std	1.1373×10^{2}	3.6189×10^{2}	6.2317×10^{1}	8.9494×10^{1}	2.8922×10^{2}	6.3085×10^{1}	3.7994×10^{0}	1.3737×10^{2}	2.1795×10^{0}	7.5251×10^{1}	1.0158×10^{2}	2.6410×10^{1}	5.0413×10^{0}	2.1836×10^{0}	1.2490×10^{0}	1.1373×10^{2}	3.6189×10^{2}
	Rank	10	6	13	17	8	14	16	12	4	15	3	7	9	11	2	5	1
9	Best	2.6984×10^{3}	2.8665×10^{3}	2.4813×10^{3}	2.7131×10^{3}	2.8827×10^{3}	2.5330×10^{3}	2.5344×10^{3}	2.5948×10^{3}	2.4808×10^{3}	2.4972×10^{3}	2.5437×10^{3}	2.4708×10^{3}	2.4812×10^{3}	2.4808×10^{3}	2.4804×10^{3}	2.6984×10^{3}	2.8665×10^{3}

	Mean	2.8291×10^{3}	3.3651×10^{3}	2.4923×10^{3}	2.8531×10^{3}	3.2468×10^{3}	2.6020×10^{3}	2.6086×10^{3}	2.7012×10^{3}	2.4808×10^{3}	2.6067×10^{3}	2.6257×10^{3}	2.4804×10^{3}	2.4908×10^{3}	2.4829×10^{3}	2.4808×10^{3}	2.8291×10^{3}	**3.3651×10^{3}**
	Std	1.2034×10^{2}	2.6654×10^{2}	8.8722×10^{0}	7.4535×10^{1}	2.5051×10^{2}	4.4570×10^{1}	5.7452×10^{1}	6.0742×10^{1}	8.8542×10^{-5}	5.3067×10^{1}	7.1747×10^{1}	1.3253×10^{1}	5.4841×10^{0}	2.7654×10^{0}	1.8015×10^{-1}	1.2034×10^{2}	2.6654×10^{2}
	Rank	8	5	14	17	6	15	16	9	10	13	2	11	12	3	7	4	1
10	Best	2.5967×10^{3}	2.6974×10^{3}	2.5009×10^{3}	2.5890×10^{3}	2.6011×10^{3}	2.5129×10^{3}	2.5102×10^{3}	2.5154×10^{3}	2.5004×10^{3}	2.5111×10^{3}	2.5012×10^{3}	2.6342×10^{3}	2.4130×10^{3}	2.4677×10^{3}	2.5003×10^{3}	2.5967×10^{3}	2.6974×10^{3}
	Mean	6.5732×10^{3}	6.2348×10^{3}	4.1716×10^{3}	4.4492×10^{3}	6.0570×10^{3}	4.7070×10^{3}	3.5122×10^{3}	5.2525×10^{3}	2.6427×10^{3}	3.7222×10^{3}	3.2432×10^{3}	6.4898×10^{3}	2.6022×10^{3}	2.5544×10^{3}	2.5379×10^{3}	6.5732×10^{3}	**6.2348×10^{3}**
	Std	8.8465×10^{2}	1.5075×10^{3}	9.6107×10^{2}	1.9713×10^{3}	1.6567×10^{3}	1.6951×10^{3}	1.2866×10^{3}	1.9602×10^{3}	2.6145×10^{2}	8.4870×10^{2}	9.0942×10^{2}	1.1130×10^{3}	1.7857×10^{2}	7.5385×10^{1}	7.8044×10^{1}	8.8465×10^{2}	1.5075×10^{3}
	Rank	5	7	17	15	10	12	14	11	8	13	3	9	6	16	4	2	1
11	Best	4.8034×10^{3}	8.0191×10^{3}	2.6663×10^{3}	5.3622×10^{3}	8.3278×10^{3}	3.7351×10^{3}	3.9776×10^{3}	4.0144×10^{3}	2.9000×10^{3}	3.9950×10^{3}	3.2081×10^{3}	2.9004×10^{3}	2.9138×10^{3}	2.9001×10^{3}	2.9000×10^{3}	4.8034×10^{3}	8.0191×10^{3}
	Mean	7.8057×10^{3}	1.0727×10^{4}	3.2476×10^{3}	6.6785×10^{3}	1.0470×10^{4}	4.9210×10^{3}	6.3961×10^{3}	5.0065×10^{3}	2.9392×10^{3}	5.6198×10^{3}	4.8088×10^{3}	2.9088×10^{3}	2.9886×10^{3}	2.9062×10^{3}	2.9067×10^{3}	7.8057×10^{3}	**1.0727×10^{4}**
	Std	8.9439×10^{2}	1.5193×10^{3}	3.3880×10^{2}	7.9419×10^{2}	1.0572×10^{3}	6.8215×10^{2}	1.1498×10^{3}	6.8294×10^{2}	7.7119×10^{1}	8.8196×10^{2}	8.8426×10^{2}	2.4831×10^{1}	9.1485×10^{1}	3.3376×10^{1}	2.5371×10^{1}	8.9439×10^{2}	1.5193×10^{3}
	Rank	8	4	15	16	7	14	17	10	13	11	3	12	9	5	6	2	1
12	Best	2.9588×10^{3}	2.9426×10^{3}	3.2315×10^{3}	3.5218×10^{3}	2.9572×10^{3}	3.1831×10^{3}	3.2448×10^{3}	2.9686×10^{3}	2.9446×10^{3}	2.9883×10^{3}	2.9328×10^{3}	3.0601×10^{3}	3.1149×10^{3}	2.9000×10^{3}	2.9678×10^{3}	2.9306×10^{3}	2.9329×10^{3}
	Mean	3.6161×10^{3}	3.8217×10^{3}	3.0454×10^{3}	3.5579×10^{3}	3.5413×10^{3}	3.0688×10^{3}	2.9936×10^{3}	3.0927×10^{3}	2.9536×10^{3}	3.1448×10^{3}	3.4634×10^{3}	2.9000×10^{3}	3.0005×10^{3}	**2.9479×10^{3}**	2.9460×10^{3}	3.6161×10^{3}	3.8217×10^{3}
	Std	2.7168×10^{2}	2.0157×10^{2}	8.1830×10^{1}	1.7158×10^{2}	1.8110×10^{2}	8.2464×10^{1}	3.9348×10^{1}	7.3201×10^{1}	2.3513×10^{1}	5.1408×10^{1}	1.8768×10^{2}	7.2047×10^{-5}	3.4544×10^{1}	8.5219×10^{0}	1.3504×10^{1}	2.7168×10^{2}	2.0157×10^{2}
	Rank	8	5	16	17	9	15	14	10	6	11	4	12	13	1	7	3	2
Mean rank		8.67	5.00	14.58	16.25	8.17	13.83	15.75	10.67	7.58	13.42	2.67	9.50	8.58	8.00	5.01	3.83	1.50
Result		10	4	15	17	8	14	16	12	6	13	2	11	9	7	5	3	**1**

Table S7. Wilcoxon experiment results of mESC and novel metaheuristic algorithm on CEC2022 (10 dimensions).

F	Algorithms														
	**PO**	**GMO**	**FATA**	**MGO**	**ECO**	**CPO**	**PLO**	**NRBO**	**IAO**	**LEA**	**MNEARO**	**SHO**	**ZOA**	**DMOA**	**AHA**	**ESC**
1	2.3657×10^{-12}	2.3657×10^{-12}	2.3657×10^{-12}	2.3657×10^{-12}	2.3657×10^{-12}	2.3657×10^{-12}	2.3657×10^{-12}	2.3657×10^{-12}	2.3657×10^{-12}	2.3657×10^{-12}	2.3657×10^{-12}	2.3657×10^{-12}	2.3657×10^{-12}	2.3657×10^{-12}	2.3657×10^{-12}	2.3657×10^{-12}
2	4.6159×10^{-10}	7.9590×10^{-3}	3.0199×10^{-11}	3.0199×10^{-11}	9.7917×10^{-5}	3.0199×10^{-11}	3.0199×10^{-11}	3.0199×10^{-11}	1.2212×10^{-2}	3.0199×10^{-11}	7.5617×10^{-3}	1.7294×10^{-7}	4.9980×10^{-9}	1.0576×10^{-3}	8.5338×10^{-1}	8.1465×10^{-5}
3	1.2641×10^{-11}	1.2641×10^{-11}	1.2641×10^{-11}	1.2641×10^{-11}	1.2641×10^{-11}	1.2641×10^{-11}	1.2641×10^{-11}	1.2641×10^{-11}	1.2641×10^{-11}	1.2641×10^{-11}	1.3234×10^{-9}	1.2641×10^{-11}	1.2641×10^{-11}	4.6564×10^{-10}	1.2641×10^{-11}	2.8707×10^{-10}
4	6.0034×10^{-11}	9.0054×10^{-4}	2.9878×10^{-11}	2.9878×10^{-11}	7.7098×10^{-9}	2.9878×10^{-11}	2.9878×10^{-11}	3.3031×10^{-11}	2.1191×10^{-4}	2.9878×10^{-11}	9.9410×10^{-1}	8.9025×10^{-11}	2.5555×10^{-7}	2.9878×10^{-11}	2.4177×10^{-9}	1.7621×10^{-3}
5	2.8003×10^{-11}	3.7845×10^{-11}	2.8003×10^{-11}	2.8003×10^{-11}	2.8003×10^{-11}	2.8003×10^{-11}	2.8003×10^{-11}	2.8003×10^{-11}	2.8003×10^{-11}	2.8003×10^{-11}	**2.3278×10^{-1}**	2.8003×10^{-11}	2.8003×10^{-11}	5.1046×10^{-11}	2.8003×10^{-11}	4.1823×10^{-11}
6	1.4918×10^{-6}	6.2040×10^{-1}	3.0199×10^{-11}	3.0199×10^{-11}	6.7362×10^{-6}	3.0199×10^{-11}	3.0199×10^{-11}	9.0307×10^{-4}	1.0937×10^{-10}	3.0199×10^{-11}	3.0199×10^{-11}	6.0104×10^{-8}	2.3800×10^{-3}	3.6897×10^{-11}	**1.6238×10^{-1}**	3.6709×10^{-3}
7	3.0199×10^{-11}	9.9186×10^{-11}	3.0199×10^{-11}	3.0199×10^{-11}	3.0199×10^{-11}	3.0199×10^{-11}	3.0199×10^{-11}	3.0199×10^{-11}	1.2057×10^{-10}	3.0199×10^{-11}	4.2067×10^{-2}	4.0772×10^{-11}	4.0772×10^{-11}	3.0199×10^{-11}	5.1857×10^{-7}	1.0315×10^{-2}
8	3.0199×10^{-11}	3.0199×10^{-11}	3.0199×10^{-11}	3.0199×10^{-11}	1.9568×10^{-10}	3.0199×10^{-11}	3.0199×10^{-11}	3.0199×10^{-11}	1.3832×10^{-2}	3.0199×10^{-11}	**7.0617×10^{-1}**	1.0702×10^{-9}	1.9568×10^{-10}	3.0199×10^{-11}	8.1465×10^{-5}	1.9527×10^{-3}
9	3.0199×10^{-11}	3.0199×10^{-11}	3.0199×10^{-11}	3.0199×10^{-11}	3.0199×10^{-11}	3.0199×10^{-11}	3.0199×10^{-11}	3.0199×10^{-11}	3.0199×10^{-11}	3.0199×10^{-11}	2.3638×10^{-12}	3.0199×10^{-11}	3.0199×10^{-11}	3.0199×10^{-11}	3.0199×10^{-11}	3.0199×10^{-11}

10	2.2539×10^{-4}	1.6813×10^{-4}	9.5139×10^{-6}	2.3168×10^{-6}	6.5261×10^{-7}	1.9527×10^{-3}	1.1077×10^{-6}	7.6973×10^{-4}	3.6709×10^{-3}	1.1738×10^{-3}	**1.8577×10^{-1}**	3.0103×10^{-7}	2.2780×10^{-5}	1.9112×10^{-2}	6.9724×10^{-3}	3.7782×10^{-2}
11	2.0200×10^{-8}	5.3793×10^{-9}	3.0538×10^{-11}	1.6525×10^{-11}	1.8523×10^{-8}	5.5991×10^{-11}	2.0297×10^{-11}	9.3796×10^{-10}	2.4008×10^{-8}	1.0303×10^{-9}	7.8480×10^{-8}	7.0366×10^{-9}	1.0303×10^{-9}	3.1056×10^{-8}	6.6437×10^{-8}	1.8523×10^{-8}
12	3.5201×10^{-7}	2.3168×10^{-6}	3.0199×10^{-11}	3.0199×10^{-11}	3.5923×10^{-5}	3.0199×10^{-11}	3.0199×10^{-11}	6.5277×10^{-8}	**6.8432×10^{-1}**	3.0199×10^{-11}	3.1724×10^{-3}	3.0199×10^{-11}	3.0199×10^{-11}	3.0199×10^{-11}	2.4386×10^{-9}	1.3832×10^{-2}
+/=/-	12/0/0	11/0/1	12/0/0	12/0/0	12/0/0	12/0/0	12/0/0	12/0/0	10/1/1	12/0/0	4/3/5	12/0/0	12/0/0	11/0/1	10/1/1	11/0/1

Table S8. Wilcoxon experiment results of mESC and novel metaheuristic algorithm on CEC2022 (20 dimensions).

F	Algorithms															
	PO	GMO	FATA	MGO	ECO	CPO	PLO	NRBO	IAO	LEA	MNEARO	SHO	ZOA	DMOA	AHA	ESC
1	3.3384×10^{-11}	3.0199×10^{-11}	3.0199×10^{-11}	3.0199×10^{-11}	3.0199×10^{-11}	3.0199×10^{-11}	3.0199×10^{-11}	3.0199×10^{-11}	3.3384×10^{-11}	3.0199×10^{-11}	**5.3951×10^{-1}**	3.0199×10^{-11}	3.0199×10^{-11}	3.0199×10^{-11}	3.6897×10^{-11}	3.0199×10^{-11}
2	3.0199×10^{-11}	1.9568×10^{-10}	3.0199×10^{-11}	3.0199×10^{-11}	3.0199×10^{-11}	3.0199×10^{-11}	3.0199×10^{-11}	3.0199×10^{-11}	3.0199×10^{-11}	3.0199×10^{-11}	1.1747×10^{-4}	3.0199×10^{-11}	3.0199×10^{-11}	8.4848×10^{-9}	5.5727×10^{-10}	1.2023×10^{-8}
3	3.0199×10^{-11}	3.0199×10^{-11}	3.0199×10^{-11}	3.0199×10^{-11}	3.0199×10^{-11}	3.0199×10^{-11}	3.0199×10^{-11}	3.0199×10^{-11}	3.0199×10^{-11}	3.0199×10^{-11}	4.1997×10^{-10}	3.0199×10^{-11}	3.0199×10^{-11}	3.6897×10^{-11}	3.0199×10^{-11}	9.7555×10^{-10}
4	3.0199×10^{-11}	5.5727×10^{-10}	3.0199×10^{-11}	3.0199×10^{-11}	4.0772×10^{-11}	3.0199×10^{-11}	3.0199×10^{-11}	3.0199×10^{-11}	3.0199×10^{-11}	3.0199×10^{-11}	1.0907×10^{-5}	3.0199×10^{-11}	3.3384×10^{-11}	3.0199×10^{-11}	3.0199×10^{-11}	3.3384×10^{-11}
5	3.0199×10^{-11}	6.7220×10^{-10}	3.0199×10^{-11}	3.0199×10^{-11}	3.0199×10^{-11}	3.0199×10^{-11}	3.0199×10^{-11}	3.0199×10^{-11}	3.0199×10^{-11}	3.0199×10^{-11}	3.0199×10^{-11}	3.0199×10^{-11}	3.0199×10^{-11}	3.0199×10^{-11}	3.0199×10^{-11}	2.2273×10^{-9}
6	4.0772×10^{-11}	**6.7350×10^{-1}**	3.0199×10^{-11}	3.0199×10^{-11}	1.3367×10^{-5}	3.0199×10^{-11}	3.0199×10^{-11}	3.0199×10^{-11}	1.0315×10^{-2}	3.0199×10^{-11}	**5.5546×10^{-2}**	3.0199×10^{-11}	4.5043×10^{-11}	3.0199×10^{-11}	**4.1191×10^{-1}**	**8.0727×10^{-1}**
7	2.8716×10^{-10}	3.8249×10^{-9}	3.0199×10^{-11}	3.0199×10^{-11}	4.1997×10^{-10}	3.0199×10^{-11}	3.0199×10^{-11}	9.9186×10^{-11}	8.4848×10^{-9}	4.0772×10^{-11}	1.2732×10^{-2}	5.5727×10^{-10}	1.4110×10^{-9}	5.5727×10^{-10}	1.1674×10^{-5}	2.5101×10^{-2}
8	3.0199×10^{-11}	4.5043×10^{-11}	3.0199×10^{-11}	3.0199×10^{-11}	3.0199×10^{-11}	3.0199×10^{-11}	3.0199×10^{-11}	3.0199×10^{-11}	3.3384×10^{-11}	3.0199×10^{-11}	1.0937×10^{-10}	3.0199×10^{-11}	3.0199×10^{-11}	3.0199×10^{-11}	6.5486×10^{-4}	3.0199×10^{-11}
9	3.0199×10^{-11}	3.3384×10^{-11}	3.0199×10^{-11}	3.0199×10^{-11}	3.3384×10^{-11}	3.0199×10^{-11}	3.0199×10^{-11}	3.0199×10^{-11}	3.0199×10^{-11}	3.0199×10^{-11}	**1.8577×10^{-1}**	3.0199×10^{-11}	3.0199×10^{-11}	2.6077×10^{-2}	3.3384×10^{-11}	1.3111×10^{-8}
10	1.2477×10^{-4}	1.5964×10^{-7}	5.4941×10^{-11}	3.6897×10^{-11}	6.7220×10^{-10}	2.2273×10^{-9}	5.4941×10^{-11}	3.6459×10^{-8}	3.8053×10^{-7}	4.1825×10^{-9}	1.8916×10^{-4}	4.6159×10^{-10}	3.2555×10^{-7}	4.0772×10^{-11}	1.3250×10^{-4}	7.6171×10^{-3}
11	3.0199×10^{-11}	1.4110×10^{-9}	3.0199×10^{-11}	3.0199×10^{-11}	1.6980×10^{-8}	3.0199×10^{-11}	3.0199×10^{-11}	3.0199×10^{-11}	3.0199×10^{-11}	3.0199×10^{-11}	1.6947×10^{-9}	3.0199×10^{-11}	3.0199×10^{-11}	7.1186×10^{-9}	1.4110×10^{-9}	7.1186×10^{-9}
12	3.1589×10^{-10}	9.2603×10^{-9}	3.0199×10^{-11}	3.0199×10^{-11}	1.7769×10^{-10}	3.0199×10^{-11}	3.0199×10^{-11}	4.9752×10^{-11}	1.4294×10^{-8}	3.3384×10^{-11}	**8.5000×10^{-2}**	3.0199×10^{-11}	3.0199×10^{-11}	3.0199×10^{-11}	1.7769×10^{-10}	**8.5000×10^{-2}**
+/=/-	12/0/0	11/1/0	12/0/0	12/0/0	12/0/0	12/0/0	12/0/0	12/0/0	11/0/1	12/0/0	6/4/2	12/0/0	12/0/0	10/0/2	10/1/1	10/2/0

Table S9. The running time (in seconds) of mESC and the new metaheuristic algorithm on CEC2022.

F	D	PO	GMO	FATA	MGO	ECO	CPO	PLO	NRBO	IAO	LEA	MNEARO	SHO	ZOA	DMOA	AHA	ESC	mESC
1	10	5.6487×10^{-1}	6.1650×10^{-1}	2.0450×10^{-3}	1.8965×10^{-3}	5.4150×10^{-2}	1.7205×10^{-3}	3.3679×10^{-3}	9.0282×10^{-2}	1.5510×10^{-1}	4.3451×10^{-3}	1.9067×10^{-1}	1.2146×10^{-1}	4.9561×10^{-2}	2.4301×10^{-1}	5.7078×10^{-2}	4.9617×10^{-1}	6.0796×10^{-1}
	20	8.0150×10^{-1}	6.9680×10^{-1}	2.7427×10^{-3}	2.3169×10^{-3}	6.8584×10^{-2}	2.2318×10^{-3}	4.9089×10^{-3}	1.1534×10^{-1}	2.1137×10^{-1}	5.8450×10^{-3}	8.0531×10^{-1}	2.0113×10^{-1}	6.7531×10^{-2}	2.7583×10^{-1}	6.4882×10^{-2}	5.6809×10^{-1}	7.9304×10^{-1}
2	10	6.2815×10^{-1}	7.1577×10^{-1}	2.5010×10^{-3}	2.4213×10^{-3}	6.3084×10^{-2}	1.8738×10^{-3}	3.5297×10^{-3}	1.0752×10^{-1}	1.9554×10^{-1}	6.8956×10^{-3}	3.9676×10^{-1}	1.4593×10^{-1}	5.6781×10^{-2}	3.1309×10^{-1}	7.0702×10^{-2}	6.0112×10^{-1}	7.2561×10^{-1}
	20	7.3068×10^{-1}	6.8160×10^{-1}	3.2003×10^{-3}	2.6617×10^{-3}	6.7232×10^{-2}	2.2160×10^{-3}	4.8331×10^{-3}	1.1216×10^{-1}	1.9162×10^{-1}	6.5482×10^{-3}	7.6857×10^{-1}	2.0980×10^{-1}	6.8057×10^{-2}	2.9177×10^{-1}	7.7328×10^{-2}	5.6723×10^{-1}	8.0684×10^{-1}
3	10	1.2217×10^{0}	6.9080×10^{-1}	2.8634×10^{-3}	2.6481×10^{-3}	7.9594×10^{-2}	2.2525×10^{-3}	3.9126×10^{-3}	1.1480×10^{-1}	2.5000×10^{-1}	5.2563×10^{-3}	3.0330×10^{-1}	1.5938×10^{-1}	9.8607×10^{-2}	3.0773×10^{-1}	8.3352×10^{-2}	5.4755×10^{-1}	6.8180×10^{-1}
	20	2.0347×10^{0}	7.6709×10^{-1}	4.3029×10^{-3}	3.8230×10^{-3}	1.1103×10^{-1}	3.8131×10^{-3}	6.3218×10^{-3}	1.7260×10^{-1}	3.9924×10^{-1}	8.3304×10^{-3}	9.1844×10^{-1}	2.6798×10^{-1}	1.5839×10^{-1}	3.7749×10^{-1}	1.2522×10^{-1}	1.0379×10^{0}	1.4700×10^{0}
4	10	7.9373×10^{-1}	7.8050×10^{-1}	3.2330×10^{-3}	3.3791×10^{-3}	1.0115×10^{-1}	4.1845×10^{-3}	5.2963×10^{-3}	1.5446×10^{-1}	2.5898×10^{-1}	5.7979×10^{-3}	2.9158×10^{-1}	1.4755×10^{-1}	6.4594×10^{-2}	2.7637×10^{-1}	6.8677×10^{-2}	5.1339×10^{-1}	6.3498×10^{-1}

	20	2.9829×10^{0}	9.5712×10^{-1}	4.1999×10^{-3}	3.2482×10^{-3}	9.8722×10^{-2}	3.7262×10^{-3}	6.7214×10^{-3}	1.4759×10^{-1}	2.7239×10^{-1}	6.1244×10^{-3}	6.4518×10^{-1}	2.3062×10^{-1}	9.3703×10^{-2}	3.4190×10^{-1}	9.4848×10^{-2}	7.1563×10^{-1}	1.0804×10^{0}
5	10	8.0837×10^{-1}	7.3320×10^{-1}	2.9547×10^{-3}	2.9014×10^{-3}	9.5692×10^{-2}	2.8076×10^{-3}	5.2777×10^{-3}	1.6838×10^{-1}	2.8229×10^{-1}	7.6722×10^{-3}	3.9081×10^{-1}	1.9749×10^{-1}	8.4708×10^{-2}	2.9700×10^{-1}	7.4243×10^{-2}	5.3624×10^{-1}	6.7021×10^{-1}
	20	2.0033×10^{0}	1.2033×10^{0}	5.6367×10^{-3}	3.9559×10^{-3}	1.3263×10^{-1}	4.9357×10^{-3}	6.8224×10^{-3}	2.1977×10^{-1}	3.5328×10^{-1}	7.4263×10^{-3}	6.4130×10^{-1}	2.5236×10^{-1}	1.0710×10^{-1}	4.3193×10^{-1}	9.9548×10^{-2}	7.0378×10^{-1}	1.1024×10^{0}
6	10	6.4076×10^{-1}	6.3308×10^{-1}	2.2567×10^{-3}	1.9513×10^{-3}	5.5364×10^{-2}	1.6439×10^{-3}	3.3753×10^{-3}	9.1770×10^{-2}	1.6702×10^{-1}	4.6238×10^{-3}	2.7445×10^{-1}	1.5400×10^{-1}	5.7791×10^{-2}	2.5736×10^{-1}	5.9892×10^{-2}	4.9380×10^{-1}	6.3499×10^{-1}
	20	2.1553×10^{0}	8.5378×10^{-1}	3.2795×10^{-3}	3.0010×10^{-3}	7.9422×10^{-2}	2.2183×10^{-3}	5.2398×10^{-3}	1.3149×10^{-1}	2.0766×10^{-1}	5.9461×10^{-3}	5.6421×10^{-1}	2.0490×10^{-1}	7.6451×10^{-2}	3.1653×10^{-1}	8.2085×10^{-2}	6.5300×10^{-1}	1.0258×10^{0}
7	10	1.4988×10^{0}	6.4726×10^{-1}	3.0758×10^{-3}	2.7917×10^{-3}	8.5432×10^{-2}	3.0341×10^{-3}	4.3619×10^{-3}	1.1973×10^{-1}	2.9872×10^{-1}	6.2677×10^{-3}	3.3455×10^{-1}	1.8293×10^{-1}	1.4584×10^{-1}	4.0365×10^{-1}	9.0761×10^{-2}	5.2761×10^{-1}	6.6059×10^{-1}
	20	3.7031×10^{0}	9.5625×10^{-1}	5.6981×10^{-3}	5.2300×10^{-3}	1.3906×10^{-1}	4.6909×10^{-3}	7.5990×10^{-3}	1.9595×10^{-1}	4.8098×10^{-1}	8.0264×10^{-3}	7.6577×10^{-1}	3.1879×10^{-1}	2.7448×10^{-1}	5.2796×10^{-1}	1.6534×10^{-1}	7.3086×10^{-1}	1.0742×10^{0}
8	10	1.7733×10^{0}	6.5532×10^{-1}	3.6728×10^{-3}	3.5488×10^{-3}	9.9844×10^{-2}	2.9712×10^{-3}	4.4504×10^{-3}	1.3261×10^{-1}	3.2350×10^{-1}	5.9844×10^{-3}	3.3382×10^{-1}	1.8295×10^{-1}	1.3079×10^{-1}	3.2428×10^{-1}	9.7002×10^{-2}	5.4352×10^{-1}	6.8046×10^{-1}
	20	4.9063×10^{0}	9.4750×10^{-1}	6.6144×10^{-3}	5.9671×10^{-3}	1.7373×10^{-1}	5.9897×10^{-3}	1.0183×10^{-2}	2.2118×10^{-1}	5.4162×10^{-1}	8.4474×10^{-3}	7.5588×10^{-1}	3.2185×10^{-1}	2.3648×10^{-1}	4.3957×10^{-1}	1.6422×10^{-1}	6.9684×10^{-1}	1.2719×10^{0}
9	10	1.3224×10^{0}	6.5070×10^{-1}	2.9951×10^{-3}	2.8024×10^{-3}	7.8095×10^{-2}	2.4526×10^{-3}	3.9699×10^{-3}	1.1527×10^{-1}	2.6254×10^{-1}	5.8249×10^{-3}	2.6604×10^{-1}	1.6103×10^{-1}	9.8508×10^{-2}	2.9745×10^{-1}	8.7276×10^{-2}	5.2119×10^{-1}	6.6338×10^{-1}
	20	3.7948×10^{0}	8.8549×10^{-1}	5.2463×10^{-3}	4.6330×10^{-3}	1.4162×10^{-1}	4.6072×10^{-3}	7.1189×10^{-3}	1.8464×10^{-1}	4.5439×10^{-1}	8.4110×10^{-3}	7.4876×10^{-1}	2.9910×10^{-1}	1.9538×10^{-1}	4.1767×10^{-1}	1.3794×10^{-1}	6.4460×10^{-1}	9.3624×10^{-1}
10	10	1.1964×10^{0}	6.3196×10^{-1}	2.7222×10^{-3}	2.5576×10^{-3}	7.9145×10^{-2}	2.6101×10^{-3}	4.4017×10^{-3}	1.0916×10^{-1}	2.4537×10^{-1}	5.0862×10^{-3}	2.5068×10^{-1}	1.4944×10^{-1}	9.2979×10^{-2}	2.8506×10^{-1}	7.7745×10^{-2}	5.1620×10^{-1}	6.5537×10^{-1}
	20	3.0993×10^{0}	8.7267×10^{-1}	5.0515×10^{-3}	4.2927×10^{-3}	1.2718×10^{-1}	4.4946×10^{-3}	7.4145×10^{-3}	1.6711×10^{-1}	3.6527×10^{-1}	6.6280×10^{-3}	6.1245×10^{-1}	2.5105×10^{-1}	1.5606×10^{-1}	3.6561×10^{-1}	1.0831×10^{-1}	6.1915×10^{-1}	8.9404×10^{-1}
11	10	1.7232×10^{0}	6.5704×10^{-1}	3.2082×10^{-3}	3.1102×10^{-3}	9.0764×10^{-2}	3.1243×10^{-3}	4.6127×10^{-3}	1.3170×10^{-1}	3.0875×10^{-1}	6.0826×10^{-3}	3.1881×10^{-1}	1.7507×10^{-1}	1.2783×10^{-1}	3.2415×10^{-1}	9.9352×10^{-2}	5.2342×10^{-1}	6.8750×10^{-1}
	20	4.4462×10^{0}	9.6805×10^{-1}	6.8390×10^{-3}	6.1930×10^{-3}	1.8027×10^{-1}	5.8359×10^{-3}	9.8030×10^{-3}	2.3285×10^{-1}	6.0071×10^{-1}	8.8165×10^{-3}	8.4944×10^{-1}	3.6323×10^{-1}	2.5602×10^{-1}	5.1646×10^{-1}	1.7401×10^{-1}	7.5206×10^{-1}	1.1493×10^{0}
12	10	1.8317×10^{0}	6.6749×10^{-1}	3.6002×10^{-3}	3.3819×10^{-3}	9.6898×10^{-2}	3.5256×10^{-3}	5.1915×10^{-3}	1.3703×10^{-1}	3.2724×10^{-1}	6.0535×10^{-3}	3.5396×10^{-1}	1.9156×10^{-1}	1.3226×10^{-1}	3.2898×10^{-1}	1.0094×10^{-1}	5.5215×10^{-1}	7.6052×10^{-1}
	20	5.4250×10^{0}	8.9754×10^{-1}	6.3424×10^{-3}	5.9285×10^{-3}	1.7208×10^{-1}	5.8334×10^{-3}	8.7071×10^{-3}	2.1707×10^{-1}	6.0995×10^{-1}	9.4616×10^{-3}	8.0786×10^{-1}	3.6140×10^{-1}	3.0001×10^{-1}	5.4212×10^{-1}	1.9023×10^{-1}	7.7764×10^{-1}	1.0753×10^{0}
10 D mean time		1.1670×10^{0}	6.7330×10^{-1}	2.9273×10^{-3}	2.7825×10^{-3}	8.1601×10^{-2}	2.6834×10^{-3}	4.3123×10^{-3}	1.2273×10^{-1}	2.5625×10^{-1}	5.8242×10^{-3}	3.0879×10^{-1}	1.6407×10^{-1}	9.5021×10^{-2}	3.0484×10^{-1}	8.0585×10^{-2}	5.3103×10^{-1}	6.7195×10^{-1}
10D mean rank		17	16	3	2	7	**1**	4	9	11	5	13	10	8	12	6	14	15
20 D mean time		3.0069×10^{0}	8.9060×10^{-1}	4.9295×10^{-3}	4.2709×10^{-3}	1.2430×10^{-1}	4.2161×10^{-3}	7.1394×10^{-3}	1.7648×10^{-1}	3.9071×10^{-1}	7.5009×10^{-3}	7.4026×10^{-1}	2.7352×10^{-1}	1.6580×10^{-1}	4.0374×10^{-1}	1.2366×10^{-1}	7.0556×10^{-1}	1.0566×10^{0}
20D mean ran		17	15	3	2	7	**1**	4	9	11	5	14	10	8	12	6	13	16

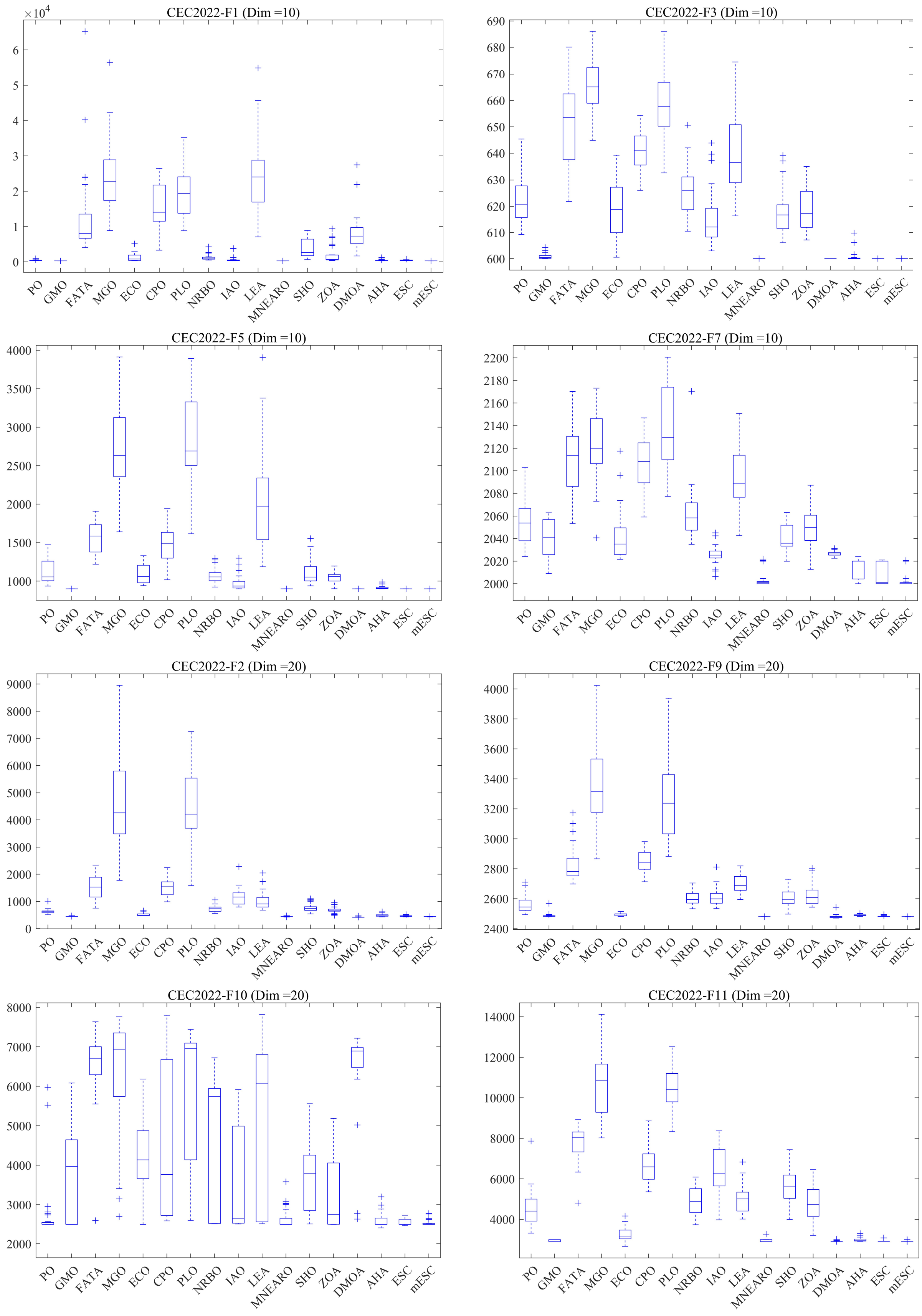

Figure S2. Box plot of mESC and novel metaheuristic algorithm on CEC2022.

Table S10. Results of mESC and High Performance, Winner Algorithm on CEC2022 (10 Dimensions).

F	Index	AGPSO	CPSOGSA	TACPSO	BDE	BeSD	MDE	MadDE	LSHADE-cnEpSin	LSHADE-SPACMA	LSHADE	ESC	mESC
1	Best	3.0000×10^{2}	3.0000×10^{2}	3.0000×10^{2}	5.6150×10^{2}	6.6825×10^{2}	1.3088×10^{3}	3.0029×10^{2}	1.8016×10^{3}	4.7138×10^{3}	4.6716×10^{3}	3.0821×10^{2}	3.0000×10^{2}
	Mean	3.0005×10^{2}	1.9763×10^{3}	3.0010×10^{2}	1.2098×10^{3}	1.1423×10^{3}	4.0525×10^{3}	5.8909×10^{2}	7.9509×10^{3}	1.7203×10^{4}	1.2733×10^{4}	4.8782×10^{2}	$\mathbf{3.0000\times10^{2}}$
	Std	1.2399×10^{-1}	1.7790×10^{3}	8.1137×10^{-7}	5.1927×10^{2}	3.7221×10^{2}	1.4005×10^{3}	3.6554×10^{2}	4.4077×10^{3}	7.8380×10^{3}	5.0324×10^{3}	2.1820×10^{2}	2.1069×10^{-11}
	Rank	3	8	2	6	7	9	5	10	12	11	4	1
2	Best	4.0031×10^{2}	4.0005×10^{2}	4.0000×10^{2}	4.0003×10^{2}	4.0487×10^{2}	4.0265×10^{2}	4.0000×10^{2}	4.1154×10^{2}	5.6141×10^{2}	4.1090×10^{2}	4.0044×10^{2}	4.0001×10^{2}
	Mean	4.2533×10^{2}	4.3534×10^{2}	4.0959×10^{2}	4.0262×10^{2}	4.1617×10^{2}	4.0917×10^{2}	$\mathbf{4.0086\times10^{2}}$	4.4590×10^{2}	7.1838×10^{2}	4.5356×10^{2}	4.0955×10^{2}	4.0516×10^{2}
	Std	5.0419×10^{1}	3.4391×10^{1}	1.6362×10^{1}	3.3987×10^{0}	8.0667×10^{0}	6.4798×10^{0}	2.3220×10^{0}	2.5662×10^{1}	1.1173×10^{2}	2.6931×10^{1}	1.1432×10^{1}	3.6140×10^{0}
	Rank	7	8	4	2	9	6	1	10	12	11	5	3
3	Best	6.0000×10^{2}	6.2971×10^{2}	6.0000×10^{2}	6.0004×10^{2}	6.0352×10^{2}	6.0000×10^{2}	6.0001×10^{2}	6.0730×10^{2}	6.2878×10^{2}	6.0780×10^{2}	6.0000×10^{2}	6.0000×10^{2}
	Mean	6.0011×10^{2}	6.4929×10^{2}	6.0021×10^{2}	6.0016×10^{2}	6.0687×10^{2}	6.0007×10^{2}	6.0003×10^{2}	6.1507×10^{2}	6.4792×10^{2}	6.1625×10^{2}	6.0003×10^{2}	$\mathbf{6.0000\times10^{2}}$
	Std	3.1732×10^{-1}	1.5352×10^{1}	4.1252×10^{-1}	6.7644×10^{-2}	1.6500×10^{0}	2.1046×10^{-4}	2.8775×10^{-2}	7.4424×10^{0}	9.5745×10^{0}	5.3376×10^{0}	6.7407×10^{-5}	9.0067×10^{-6}
	Rank	4	11	6	7	8	3	5	9	12	10	2	1
4	Best	8.0298×10^{2}	8.2189×10^{2}	8.0497×10^{2}	8.0832×10^{2}	8.1806×10^{2}	8.0463×10^{2}	8.0600×10^{2}	8.2615×10^{2}	8.5741×10^{2}	8.3031×10^{2}	8.0546×10^{2}	8.0298×10^{2}
	Mean	8.1563×10^{2}	8.4117×10^{2}	8.1523×10^{2}	8.2200×10^{2}	8.2490×10^{2}	8.1373×10^{2}	8.1240×10^{2}	8.5156×10^{2}	8.7734×10^{2}	8.5613×10^{2}	8.1274×10^{2}	$\mathbf{8.0919\times10^{2}}$
	Std	6.4920×10^{0}	1.1840×10^{1}	7.7834×10^{0}	5.2917×10^{0}	3.7752×10^{0}	6.0237×10^{0}	3.1274×10^{0}	1.0808×10^{1}	9.3695×10^{0}	1.0675×10^{1}	3.9662×10^{0}	4.1780×10^{0}
	Rank	6	9	5	7	8	4	2	10	12	11	3	1
5	Best	9.0000×10^{2}	9.3296×10^{2}	9.0000×10^{2}	9.0018×10^{2}	9.0409×10^{2}	9.0000×10^{2}	9.0002×10^{2}	9.3560×10^{2}	1.2053×10^{3}	9.2255×10^{2}	9.0000×10^{2}	9.0000×10^{2}
	Mean	9.0197×10^{2}	1.6097×10^{3}	9.0353×10^{2}	9.0149×10^{2}	9.1693×10^{2}	9.0003×10^{2}	9.0306×10^{2}	1.0717×10^{3}	1.7943×10^{3}	1.1740×10^{3}	9.0001×10^{2}	$\mathbf{9.0000\times10^{2}}$
	Std	3.3198×10^{0}	5.3609×10^{2}	8.1830×10^{0}	1.2367×10^{0}	8.4291×10^{0}	1.4139×10^{-1}	5.0453×10^{0}	1.4253×10^{2}	3.0223×10^{2}	1.2874×10^{2}	1.6346×10^{-2}	3.7290×10^{-9}
	Rank	4	11	5	6	8	3	7	9	12	10	2	1
6	Best	1.8681×10^{3}	1.8665×10^{3}	1.8084×10^{3}	1.8410×10^{3}	2.0978×10^{3}	1.8536×10^{3}	1.8082×10^{3}	2.5745×10^{4}	6.9186×10^{6}	7.4687×10^{4}	1.8601×10^{3}	1.8060×10^{3}
	Mean	4.2938×10^{3}	3.4561×10^{3}	3.7698×10^{3}	2.4590×10^{3}	4.0625×10^{3}	3.1070×10^{3}	$\mathbf{1.9577\times10^{3}}$	5.5911×10^{5}	5.9711×10^{7}	1.0730×10^{6}	3.2139×10^{3}	2.6975×10^{3}
	Std	2.3415×10^{3}	1.9237×10^{3}	1.7386×10^{3}	7.4279×10^{2}	1.6432×10^{3}	1.2321×10^{3}	2.8996×10^{2}	9.4864×10^{5}	4.0254×10^{7}	1.0404×10^{6}	1.6012×10^{3}	9.8118×10^{2}
	Rank	8	6	7	2	9	4	1	10	12	11	5	3
7	Best	2.0200×10^{3}	2.0200×10^{3}	2.0016×10^{3}	2.0068×10^{3}	2.0290×10^{3}	2.0000×10^{3}	2.0007×10^{3}	2.0489×10^{3}	2.0794×10^{3}	2.0406×10^{3}	2.0000×10^{3}	2.0000×10^{3}
	Mean	2.0216×10^{3}	2.0826×10^{3}	2.0208×10^{3}	2.0144×10^{3}	2.0384×10^{3}	2.0094×10^{3}	2.0053×10^{3}	2.0703×10^{3}	2.1264×10^{3}	2.0678×10^{3}	2.0068×10^{3}	$\mathbf{2.0052\times10^{3}}$
	Std	1.0542×10^{0}	4.4043×10^{1}	7.3210×10^{0}	5.0983×10^{0}	4.9358×10^{0}	9.5805×10^{0}	4.2297×10^{0}	1.4135×10^{1}	2.6296×10^{1}	2.6904×10^{1}	9.2761×10^{0}	8.5524×10^{0}
	Rank	7	11	6	5	8	4	3	10	12	9	2	1
8	Best	2.2203×10^{3}	2.2238×10^{3}	2.2010×10^{3}	2.2084×10^{3}	2.2199×10^{3}	2.2011×10^{3}	2.2054×10^{3}	2.2274×10^{3}	2.2434×10^{3}	2.2317×10^{3}	2.2019×10^{3}	2.2001×10^{3}
	Mean	2.2227×10^{3}	2.2911×10^{3}	2.2333×10^{3}	2.2210×10^{3}	2.2255×10^{3}	2.2171×10^{3}	2.2183×10^{3}	2.2399×10^{3}	2.2786×10^{3}	2.2426×10^{3}	2.2176×10^{3}	2.2152×10^{3}
	Std	2.2362×10^{0}	6.3449×10^{1}	3.7331×10^{1}	3.8308×10^{0}	2.0295×10^{0}	7.2863×10^{0}	5.2081×10^{0}	1.9947×10^{1}	2.7973×10^{1}	2.2150×10^{1}	7.5368×10^{0}	8.6168×10^{0}

	Rank	7	11	5	6	8	2	3	9	12	10	4	1
9	Best	2.5293×10^{3}	2.5293×10^{3}	2.5293×10^{3}	2.5293×10^{3}	2.5384×10^{3}	2.5293×10^{3}	2.5293×10^{3}	2.5381×10^{3}	2.6494×10^{3}	2.5389×10^{3}	2.5293×10^{3}	2.5002×10^{3}
	Mean	2.5294×10^{3}	2.5317×10^{3}	2.5344×10^{3}	2.5296×10^{3}	2.5498×10^{3}	2.5293×10^{3}	2.5293×10^{3}	2.5783×10^{3}	2.7213×10^{3}	2.5710×10^{3}	2.5293×10^{3}	**2.5222×10^{3}**
	Std	2.1075×10^{-1}	7.2756×10^{0}	2.6799×10^{1}	2.0364×10^{-1}	7.0446×10^{0}	1.1019×10^{-6}	7.9522×10^{-6}	4.0880×10^{1}	3.4315×10^{1}	2.7560×10^{1}	5.1934×10^{-9}	1.0125×10^{1}
	Rank	2	7	3	8	9	5	6	10	12	11	4	1
10	Best	2.5004×10^{3}	2.5004×10^{3}	2.5004×10^{3}	2.5003×10^{3}	2.5004×10^{3}	2.5002×10^{3}	2.5003×10^{3}	2.5009×10^{3}	2.5131×10^{3}	2.5009×10^{3}	2.5003×10^{3}	2.5002×10^{3}
	Mean	2.5459×10^{3}	2.6880×10^{3}	2.5316×10^{3}	2.5005×10^{3}	2.5008×10^{3}	**2.5078×10^{3}**	2.5078×10^{3}	2.5998×10^{3}	2.5936×10^{3}	2.5707×10^{3}	2.5366×10^{3}	2.5366×10^{3}
	Std	8.3152×10^{1}	3.8923×10^{2}	5.2014×10^{1}	1.0336×10^{-1}	2.0292×10^{-1}	2.8042×10^{1}	2.7996×10^{1}	7.6310×10^{1}	8.9924×10^{1}	8.3639×10^{1}	5.2192×10^{1}	5.2307×10^{1}
	Rank	9	8	7	3	6	1	5	12	11	10	4	2
11	Best	2.6000×10^{3}	2.6000×10^{3}	2.6000×10^{3}	2.6007×10^{3}	2.6827×10^{3}	2.6001×10^{3}	2.6000×10^{3}	2.7207×10^{3}	2.8533×10^{3}	2.7492×10^{3}	2.6000×10^{3}	2.6000×10^{3}
	Mean	2.8075×10^{3}	2.7868×10^{3}	2.8090×10^{3}	2.6081×10^{3}	2.7342×10^{3}	2.6529×10^{3}	2.6383×10^{3}	2.8119×10^{3}	3.0971×10^{3}	2.8165×10^{3}	2.6835×10^{3}	**2.6007×10^{3}**
	Std	1.3901×10^{2}	1.6033×10^{2}	1.4846×10^{2}	2.7016×10^{1}	1.5596×10^{1}	6.8855×10^{1}	3.5587×10^{0}	1.3293×10^{2}	2.2093×10^{2}	1.3814×10^{2}	1.1776×10^{2}	1.0480×10^{2}
	Rank	9	7	8	3	6	5	2	10	12	11	4	1
12	Best	2.8615×10^{3}	2.8651×10^{3}	2.8615×10^{3}	2.8621×10^{3}	2.8708×10^{3}	2.8586×10^{3}	2.8594×10^{3}	2.8672×10^{3}	2.8845×10^{3}	2.8659×10^{3}	2.8614×10^{3}	2.8564×10^{3}
	Mean	2.8659×10^{3}	2.9182×10^{3}	2.8672×10^{3}	2.8649×10^{3}	2.8772×10^{3}	**2.8630×10^{3}**	2.8631×10^{3}	2.8726×10^{3}	2.9201×10^{3}	2.8711×10^{3}	2.8641×10^{3}	2.8633×10^{3}
	Std	3.3735×10^{0}	7.0609×10^{1}	2.8317×10^{0}	8.4782×10^{-1}	2.3707×10^{0}	1.5396×10^{0}	1.3010×10^{0}	5.3616×10^{0}	2.8709×10^{1}	6.2672×10^{0}	9.7664×10^{-1}	2.7402×10^{0}
	Rank	5	11	7	6	10	1	2	9	12	8	4	3
Mean Rank		5.92	9.00	5.42	5.08	8.00	3.92	3.50	9.83	11.92	10.25	3.58	1.58
Result		7	9	6	5	8	4	2	10	12	11	3	**1**

Table S11. Results of mESC and High Performance, Winner Algorithm on CEC2022 (20 Dimensions).

F	Index	AGPSO	CPSOGSA	TACPSO	BDE	BeSD	MDE	MadDE	LSHADE-cnEpSin	LSHADE-SPACMA	LSHADE	ESC	mESC
1	Best	1.5218×10^{3}	1.1969×10^{4}	3.8808×10^{2}	7.4847×10^{3}	7.4865×10^{3}	2.3588×10^{4}	9.1095×10^{3}	2.6057×10^{4}	4.8244×10^{4}	3.3130×10^{4}	7.1891×10^{3}	3.3815×10^{2}
	Mean	1.3990×10^{4}	3.8889×10^{4}	2.7890×10^{3}	1.4063×10^{4}	1.2213×10^{4}	4.3807×10^{4}	1.9983×10^{4}	6.4507×10^{4}	9.3977×10^{4}	5.6943×10^{4}	1.6114×10^{4}	**7.9525×10^{2}**
	Std	7.6679×10^{3}	1.5690×10^{4}	2.9768×10^{3}	3.4846×10^{3}	2.8698×10^{3}	9.4347×10^{3}	4.8278×10^{3}	2.1546×10^{4}	4.5607×10^{4}	1.5785×10^{4}	5.8917×10^{3}	6.1454×10^{2}
	Rank	4	8	2	5	3	9	7	11	12	10	6	1
2	Best	4.0669×10^{2}	4.4938×10^{2}	4.0405×10^{2}	4.2725×10^{2}	5.3103×10^{2}	4.4519×10^{2}	4.4742×10^{2}	5.9489×10^{2}	2.0956×10^{3}	5.3085×10^{2}	4.4913×10^{2}	4.4483×10^{2}
	Mean	4.6634×10^{2}	4.8587×10^{2}	4.5459×10^{2}	4.6590×10^{2}	5.8950×10^{2}	4.5615×10^{2}	4.5803×10^{2}	8.7087×10^{2}	3.8967×10^{3}	6.3821×10^{2}	4.6094×10^{2}	**4.5019×10^{2}**
	Std	3.7307×10^{1}	3.5487×10^{1}	2.3552×10^{1}	1.1529×10^{1}	2.7121×10^{1}	1.1773×10^{1}	1.1886×10^{1}	1.4443×10^{2}	1.0324×10^{3}	6.6785×10^{1}	1.3641×10^{1}	6.2407×10^{0}
	Rank	4	8	2	7	9	5	3	11	12	10	6	1
3	Best	6.0054×10^{2}	6.3734×10^{2}	6.0098×10^{2}	6.0120×10^{2}	6.2166×10^{2}	6.0002×10^{2}	6.0072×10^{2}	6.3687×10^{2}	6.8956×10^{2}	6.2210×10^{2}	6.0000×10^{2}	6.0000×10^{2}
	Mean	6.0413×10^{2}	6.6241×10^{2}	6.0399×10^{2}	6.0206×10^{2}	6.2934×10^{2}	6.0004×10^{2}	6.0168×10^{2}	6.5411×10^{2}	7.0425×10^{2}	6.3838×10^{2}	6.0001×10^{2}	**6.0000×10^{2}**
	Std	2.1520×10^{0}	1.3186×10^{1}	1.9415×10^{0}	5.2068×10^{-1}	3.4538×10^{0}	2.9933×10^{-2}	7.5765×10^{-1}	9.5524×10^{0}	8.2752×10^{0}	9.4218×10^{0}	3.8060×10^{-2}	1.0452×10^{-4}

	Rank	7	11	6	5	8	3	4	10	12	9	2	1
4	Best	8.2320×10^{2}	8.7462×10^{2}	8.2192×10^{2}	8.6456×10^{2}	8.6598×10^{2}	8.7313×10^{2}	8.5351×10^{2}	9.5486×10^{2}	9.9867×10^{2}	9.3808×10^{2}	8.3470×10^{2}	8.0995×10^{2}
	Mean	8.4788×10^{2}	9.1581×10^{2}	8.4929×10^{2}	8.9645×10^{2}	9.1104×10^{2}	8.9689×10^{2}	8.7106×10^{2}	9.8722×10^{2}	1.0409×10^{3}	9.6969×10^{2}	8.6266×10^{2}	**8.2488×10^{2}**
	Std	1.4474×10^{1}	2.5124×10^{1}	1.7016×10^{1}	1.4709×10^{1}	1.4092×10^{1}	1.1512×10^{1}	8.8543×10^{0}	1.4788×10^{1}	1.9293×10^{1}	1.3599×10^{1}	1.0545×10^{1}	7.7554×10^{0}
	Rank	3	9	2	6	8	7	5	11	12	10	4	1
5	Best	9.4978×10^{2}	1.7068×10^{3}	9.0535×10^{2}	9.3939×10^{2}	1.0999×10^{3}	9.0069×10^{2}	1.0541×10^{3}	2.3625×10^{3}	4.9707×10^{3}	1.8944×10^{3}	9.0000×10^{2}	9.0000×10^{2}
	Mean	1.1808×10^{3}	3.0446×10^{3}	1.0900×10^{3}	1.0334×10^{3}	1.4844×10^{3}	9.0211×10^{2}	1.6007×10^{3}	4.2622×10^{3}	7.6158×10^{3}	3.0049×10^{3}	9.0089×10^{2}	**9.0003×10^{2}**
	Std	2.1974×10^{2}	6.6630×10^{2}	1.7575×10^{2}	6.3802×10^{1}	1.6628×10^{2}	1.2707×10^{0}	2.4103×10^{2}	1.1616×10^{3}	1.2322×10^{3}	7.8196×10^{2}	1.4958×10^{0}	8.6397×10^{-2}
	Rank	6	10	4	5	7	3	8	11	12	9	2	1
6	Best	2.0264×10^{3}	1.9197×10^{3}	1.9229×10^{3}	6.0540×10^{4}	5.6719×10^{5}	7.9112×10^{3}	5.8595×10^{3}	2.8053×10^{7}	2.9925×10^{8}	1.0741×10^{7}	1.9879×10^{3}	1.8569×10^{3}
	Mean	1.0924×10^{5}	6.4158×10^{3}	5.5671×10^{3}	3.3612×10^{5}	2.2102×10^{6}	2.2916×10^{5}	4.6045×10^{4}	2.3548×10^{8}	2.9964×10^{9}	7.3010×10^{7}	**4.0424×10^{3}**	4.0996×10^{3}
	Std	3.7548×10^{5}	5.0758×10^{3}	4.7097×10^{3}	2.4422×10^{5}	1.1881×10^{6}	4.0850×10^{5}	3.1869×10^{4}	1.8202×10^{8}	1.4412×10^{9}	4.8225×10^{7}	2.9895×10^{3}	2.0085×10^{3}
	Rank	5	4	3	8	9	7	6	11	12	10	1	2
7	Best	2.0318×10^{3}	2.0725×10^{3}	2.0363×10^{3}	2.0452×10^{3}	2.0816×10^{3}	2.0277×10^{3}	2.0489×10^{3}	2.1650×10^{3}	2.2356×10^{3}	2.1139×10^{3}	2.0189×10^{3}	2.0063×10^{3}
	Mean	2.0695×10^{3}	2.2214×10^{3}	2.0694×10^{3}	2.0661×10^{3}	2.1125×10^{3}	2.0477×10^{3}	2.0689×10^{3}	2.2535×10^{3}	2.3517×10^{3}	2.2249×10^{3}	2.0344×10^{3}	**2.0300×10^{3}**
	Std	3.4978×10^{1}	8.8499×10^{1}	2.4055×10^{1}	1.4829×10^{1}	1.4880×10^{1}	1.1688×10^{1}	1.4499×10^{1}	6.0457×10^{1}	6.3513×10^{1}	7.3348×10^{1}	7.2921×10^{0}	1.0650×10^{1}
	Rank	4	9	6	5	8	3	7	11	12	10	2	1
8	Best	2.2240×10^{3}	2.2285×10^{3}	2.2217×10^{3}	2.2261×10^{3}	2.2322×10^{3}	2.2239×10^{3}	2.2252×10^{3}	2.2595×10^{3}	2.5063×10^{3}	2.2520×10^{3}	2.2275×10^{3}	2.2208×10^{3}
	Mean	2.2559×10^{3}	2.4035×10^{3}	2.2373×10^{3}	2.2288×10^{3}	2.2395×10^{3}	2.2306×10^{3}	2.2271×10^{3}	2.3765×10^{3}	3.1661×10^{3}	2.3340×10^{3}	2.2351×10^{3}	**2.2220×10^{3}**
	Std	4.9370×10^{1}	1.4779×10^{2}	3.0061×10^{1}	1.9139×10^{0}	4.0985×10^{0}	2.5327×10^{0}	1.4676×10^{0}	9.3990×10^{1}	7.6840×10^{2}	8.0198×10^{1}	2.1223×10^{1}	1.0421×10^{0}
	Rank	7	9	4	3	8	5	2	11	12	10	6	1
9	Best	2.4808×10^{3}	2.4808×10^{3}	2.4808×10^{3}	2.4823×10^{3}	2.5141×10^{3}	2.4808×10^{3}	2.4808×10^{3}	2.5486×10^{3}	2.8613×10^{3}	2.5071×10^{3}	2.4809×10^{3}	2.4753×10^{3}
	Mean	2.4951×10^{3}	2.4901×10^{3}	2.4900×10^{3}	2.4840×10^{3}	2.5283×10^{3}	2.4811×10^{3}	2.4809×10^{3}	2.6385×10^{3}	3.3684×10^{3}	2.5845×10^{3}	2.4836×10^{3}	**2.4806×10^{3}**
	Std	2.3616×10^{1}	1.3430×10^{1}	1.7477×10^{1}	1.3284×10^{0}	6.5543×10^{0}	5.2573×10^{-1}	7.8414×10^{-2}	4.2757×10^{1}	2.3848×10^{2}	4.6238×10^{1}	2.9427×10^{0}	1.0203×10^{0}
	Rank	5	8	4	7	9	3	2	11	12	10	6	1
10	Best	2.5008×10^{3}	2.5016×10^{3}	2.5007×10^{3}	2.5007×10^{3}	2.5074×10^{3}	2.4127×10^{3}	2.4802×10^{3}	2.5150×10^{3}	2.6295×10^{3}	2.5235×10^{3}	2.4182×10^{3}	2.4082×10^{3}
	Mean	3.0154×10^{3}	4.5357×10^{3}	2.9247×10^{3}	2.5069×10^{3}	2.5235×10^{3}	2.5020×10^{3}	2.5109×10^{3}	4.5136×10^{3}	6.5379×10^{3}	4.8922×10^{3}	2.5262×10^{3}	**2.4906×10^{3}**
	Std	6.9691×10^{2}	8.9543×10^{2}	6.0926×10^{2}	3.2894×10^{1}	1.0852×10^{1}	4.9708×10^{1}	3.9172×10^{1}	2.0481×10^{3}	1.5808×10^{3}	1.9986×10^{3}	7.4918×10^{1}	8.1388×10^{1}
	Rank	8	11	6	4	7	2	5	9	12	10	3	1
11	Best	2.6000×10^{3}	2.6000×10^{3}	2.6000×10^{3}	2.9474×10^{3}	3.6133×10^{3}	2.9022×10^{3}	2.9010×10^{3}	3.8508×10^{3}	7.9344×10^{3}	3.5468×10^{3}	2.6001×10^{3}	2.9000×10^{3}
	Mean	3.5228×10^{3}	2.9731×10^{3}	2.9987×10^{3}	3.0416×10^{3}	3.8548×10^{3}	2.9224×10^{3}	2.9127×10^{3}	5.1138×10^{3}	9.9721×10^{3}	4.3962×10^{3}	2.9039×10^{3}	**2.9067×10^{3}**
	Std	5.9176×10^{2}	2.4753×10^{2}	1.9412×10^{2}	1.0034×10^{2}	1.2105×10^{2}	6.0713×10^{1}	3.0681×10^{1}	6.1525×10^{2}	9.3041×10^{2}	4.9131×10^{2}	6.7523×10^{1}	2.5371×10^{1}
	Rank	8	2	4	7	9	6	5	11	12	10	3	1
12	Best	2.9449×10^{3}	3.0317×10^{3}	2.9473×10^{3}	2.9645×10^{3}	3.0945×10^{3}	2.9333×10^{3}	2.9480×10^{3}	2.9963×10^{3}	3.4894×10^{3}	2.9882×10^{3}	2.9328×10^{3}	2.9354×10^{3}
	Mean	2.9925×10^{3}	3.3193×10^{3}	2.9830×10^{3}	2.9770×10^{3}	3.1401×10^{3}	**2.9428×10^{3}**	2.9625×10^{3}	3.0946×10^{3}	3.7596×10^{3}	3.0370×10^{3}	2.9477×10^{3}	2.9468×10^{3}
	Std	5.6361×10^{1}	1.7147×10^{2}	2.9103×10^{1}	8.1017×10^{0}	2.1919×10^{1}	3.3898×10^{0}	8.2680×10^{0}	6.1131×10^{1}	1.7846×10^{2}	3.0946×10^{1}	8.4814×10^{0}	1.0664×10^{1}

Rank	5	11	6	7	10	1	4	9	12	8	3	2
Mean Rank	5.50	8.33	4.08	5.75	7.92	4.50	4.83	10.58	12.00	9.67	3.67	1.17
Result	6	9	3	7	8	4	5	11	12	10	2	**1**

Table S12. Wilcoxon test results of mESC and high-performance, winner algorithm on CEC2022 (10 dimensions).

F	Algorithms AGPSO	CPSOGSA	TACPSO	BDE	BeSD	MDE	MadDE	LSHADE-cnEpSin	LSHADE-SPACMA	LSHADE	ESC
1	3.6897×10^{-11}	3.0199×10^{-11}	2.1327×10^{-5}	3.0199×10^{-11}	3.0199×10^{-11}	3.0199×10^{-11}	3.0199×10^{-11}	3.0199×10^{-11}	3.0180×10^{-11}	3.0199×10^{-11}	3.0199×10^{-11}
2	7.6162×10^{-3}	5.0723×10^{-10}	2.8913×10^{-3}	1.4294×10^{-8}	3.0199×10^{-11}	4.4440×10^{-7}	6.5277×10^{-8}	3.0199×10^{-11}	3.0199×10^{-11}	3.0199×10^{-11}	4.5726×10^{-9}
3	3.0199×10^{-11}	3.0199×10^{-11}	3.0199×10^{-11}	3.0199×10^{-11}	3.0199×10^{-11}	3.0199×10^{-11}	3.0199×10^{-11}	3.0199×10^{-11}	3.0161×10^{-11}	3.0199×10^{-11}	3.3384×10^{-11}
4	1.3105×10^{-8}	3.0199×10^{-11}	1.1023×10^{-8}	3.0199×10^{-11}	3.0199×10^{-11}	3.0199×10^{-11}	3.0199×10^{-11}	3.0199×10^{-11}	3.0199×10^{-11}	3.0199×10^{-11}	4.0772×10^{-11}
5	3.0199×10^{-11}	3.0199×10^{-11}	3.0199×10^{-11}	3.0199×10^{-11}	3.0199×10^{-11}	3.0199×10^{-11}	3.0199×10^{-11}	3.0199×10^{-11}	3.0199×10^{-11}	3.0199×10^{-11}	2.6695×10^{-9}
6	3.9881×10^{-4}	**9.0490×10^{-2}**	3.8710×10^{-1}	3.0199×10^{-11}	3.0199×10^{-11}	3.6897×10^{-11}	5.4941×10^{-11}	3.0199×10^{-11}	3.0199×10^{-11}	3.0199×10^{-11}	**6.3088×10^{-1}**
7	5.4617×10^{-9}	3.0199×10^{-11}	5.0723×10^{-10}	2.8716×10^{-10}	3.0199×10^{-11}	7.5991×10^{-7}	1.0937×10^{-10}	3.0199×10^{-11}	3.0180×10^{-11}	3.0199×10^{-11}	4.0330×10^{-3}
8	4.9752×10^{-11}	3.0199×10^{-11}	5.4617×10^{-9}	3.0199×10^{-11}	3.0199×10^{-11}	3.3384×10^{-11}	3.6897×10^{-11}	3.0199×10^{-11}	3.0161×10^{-11}	3.0199×10^{-11}	3.0199×10^{-11}
9	2.3885×10^{-4}	1.7769×10^{-10}	8.5627×10^{-4}	3.0199×10^{-11}	3.0199×10^{-11}	5.2640×10^{-4}	4.8560×10^{-3}	3.0199×10^{-11}	3.0161×10^{-11}	3.0199×10^{-11}	3.8249×10^{-9}
10	4.3106×10^{-8}	8.1527×10^{-11}	7.0881×10^{-8}	7.2208×10^{-6}	6.2828×10^{-6}	4.2259×10^{-3}	1.7479×10^{-5}	3.8249×10^{-9}	4.0696×10^{-11}	3.4971×10^{-9}	6.9724×10^{-3}
11	$2.0317\times$	$1.7666\times$	$2.6015\times$	9.7555	3.0199	5.9673	4.9980	$3.0199\times$	3.0199×10^{-11}	$3.0199\times$	4.6856

	10^{-9}	10^{-3}	10^{-8}	$\times 10^{-10}$	$\times 10^{-11}$	$\times 10^{-9}$	$\times 10^{-9}$	10^{-11}		10^{-11}	$\times 10^{-8}$
12	8.3451×10^{-8}	3.0199×10^{-11}	5.4617×10^{-9}	5.0723×10^{-10}	3.0199×10^{-11}	$\mathbf{2.3399 \times 10^{-1}}$	3.0811×10^{-8}	3.0199×10^{-11}	3.0161×10^{-11}	3.3384×10^{-11}	$\mathbf{2.9727 \times 10^{-1}}$
+/=/-	12/0/0	11/1/0	12/0/0	10/0/2	10/0/2	9/1/2	9/0/3	12/0/0	12/0/0	12/0/0	10/2/0

Table S13. Wilcoxon test results of mESC and high-performance, winner algorithm on CEC2022 (20 dimensions).

F	Algorithms AGPSO	CPSOGSA	TACPSO	BDE	BeSD	MDE	MadDE	LSHADE-cnEpSin	LSHADE-SPACMA	LSHADE	ESC
1	3.6897×10^{-11}	3.0199×10^{-11}	2.1327×10^{-5}	3.0199×10^{-11}	3.0199×10^{-11}	3.0199×10^{-11}	3.0199×10^{-11}	3.0199×10^{-11}	3.0180×10^{-11}	3.0199×10^{-11}	3.0199×10^{-11}
2	7.6162×10^{-3}	5.0723×10^{-10}	2.8913×10^{-3}	1.4294×10^{-8}	3.0199×10^{-11}	4.4440×10^{-7}	6.5277×10^{-8}	3.0199×10^{-11}	3.0199×10^{-11}	3.0199×10^{-11}	4.5726×10^{-9}
3	3.0199×10^{-11}	3.0199×10^{-11}	3.0199×10^{-11}	3.0199×10^{-11}	3.0199×10^{-11}	3.0199×10^{-11}	3.0199×10^{-11}	3.0199×10^{-11}	3.0161×10^{-11}	3.0199×10^{-11}	3.3384×10^{-11}
4	1.3105×10^{-8}	3.0199×10^{-11}	1.1023×10^{-8}	3.0199×10^{-11}	3.0199×10^{-11}	3.0199×10^{-11}	3.0199×10^{-11}	3.0199×10^{-11}	3.0199×10^{-11}	3.0199×10^{-11}	4.0772×10^{-11}
5	3.0199×10^{-11}	3.0199×10^{-11}	3.0199×10^{-11}	3.0199×10^{-11}	3.0199×10^{-11}	3.0199×10^{-11}	3.0199×10^{-11}	3.0199×10^{-11}	3.0199×10^{-11}	3.0199×10^{-11}	2.6695×10^{-9}
6	3.9881×10^{-4}	$\mathbf{9.0490 \times 10^{-2}}$	$\mathbf{3.8710 \times 10^{-1}}$	3.0199×10^{-11}	3.0199×10^{-11}	3.6897×10^{-11}	5.4941×10^{-11}	3.0199×10^{-11}	3.0199×10^{-11}	3.0199×10^{-11}	$\mathbf{6.3088 \times 10^{-1}}$
7	5.4617×10^{-9}	3.0199×10^{-11}	5.0723×10^{-10}	2.8716×10^{-10}	3.0199×10^{-11}	7.5991×10^{-7}	1.0937×10^{-10}	3.0199×10^{-11}	3.0180×10^{-11}	3.0199×10^{-11}	4.0330×10^{-3}
8	4.9752×10^{-11}	3.0199×10^{-11}	5.4617×10^{-9}	3.0199×10^{-11}	3.0199×10^{-11}	3.3384×10^{-11}	3.6897×10^{-11}	3.0199×10^{-11}	3.0161×10^{-11}	3.0199×10^{-11}	3.0199×10^{-11}

9	2.3885 × 10^{-4}	1.7769 × 10^{-10}	8.5627 × 10^{-4}	3.0199 × 10^{-11}	3.0199 × 10^{-11}	5.2640 × 10^{-4}	4.8560 × 10^{-3}	3.0199 × 10^{-11}	3.0161 × 10^{-11}	3.0199 × 10^{-11}	3.8249 × 10^{-9}
10	4.3106 × 10^{-8}	8.1527 × 10^{-11}	7.0881 × 10^{-8}	7.2208 × 10^{-6}	6.2828 × 10^{-6}	4.2259 × 10^{-3}	1.7479 × 10^{-5}	3.8249 × 10^{-9}	4.0696 × 10^{-11}	3.4971 × 10^{-9}	6.9724 × 10^{-3}
11	2.0317 × 10^{-9}	1.7666 × 10^{-3}	2.6015 × 10^{-8}	9.7555 × 10^{-10}	3.0199 × 10^{-11}	5.9673 × 10^{-9}	4.9980 × 10^{-9}	3.0199 × 10^{-11}	3.0199 × 10^{-11}	3.0199 × 10^{-11}	4.6856 × 10^{-8}
12	8.3451 × 10^{-8}	3.0199 × 10^{-11}	5.4617 × 10^{-9}	5.0723 × 10^{-10}	3.0199 × 10^{-11}	**2.3399 × 10^{-1}**	3.0811 × 10^{-8}	3.0199 × 10^{-11}	3.0161 × 10^{-11}	3.3384 × 10^{-11}	**2.9727 × 10^{-1}**
+/=/-	12/0/0	11/1/0	11/1/0	12/0/0	12/0/0	10/1/1	12/0/0	12/0/0	12/0/0	12/0/0	9/2/1

Table S14. mESC and High Performance, Winner Algorithm Run Time on CEC2022 (in seconds).

F	D	AGPSO	CPSOGSA	TACPSO	BDE	BeSD	MDE	MadDE	LSHADE-cnEpSin	LSHADE-SPACMA	LSHADE	ESC	mESC
1	10	5.7353 × 10^{-2}	1.6157 × 10^{-1}	3.7529 × 10^{-2}	6.7017 × 10^{-2}	1.5131 × 10^{-1}	4.5524 × 10^{-2}	2.1500 × 10^{-1}	7.6436 × 10^{-2}	8.7500 × 10^{-3}	7.6203 × 10^{-3}	6.6444 × 10^{-1}	1.2402 × 10^{0}
	20	1.2947 × 10^{-1}	4.0924 × 10^{-1}	9.0400 × 10^{-2}	1.5171 × 10^{-1}	3.0119 × 10^{-1}	1.0561 × 10^{-1}	2.3979 × 10^{-1}	8.5430 × 10^{-2}	1.5397 × 10^{-2}	9.8109 × 10^{-3}	9.8304 × 10^{-1}	9.5744 × 10^{-1}
2	10	4.4814 × 10^{-2}	1.2974 × 10^{-1}	3.0561 × 10^{-2}	6.1490 × 10^{-2}	1.4037 × 10^{-1}	4.6038 × 10^{-2}	1.5290 × 10^{-1}	4.2888 × 10^{-2}	6.8802 × 10^{-3}	6.3529 × 10^{-3}	5.9799 × 10^{-1}	7.2822 × 10^{-1}
	20	1.6531 × 10^{-1}	5.5527 × 10^{-1}	1.3328 × 10^{-1}	1.9491 × 10^{-1}	4.0178 × 10^{-1}	1.3985 × 10^{-1}	2.4852 × 10^{-1}	1.2042 × 10^{-1}	1.5076 × 10^{-2}	1.5962 × 10^{-2}	1.4187 × 10^{0}	1.1284 × 10^{0}
3	10	1.2903 × 10^{-1}	2.6093 × 10^{-1}	1.1052 × 10^{-1}	1.5999 × 10^{-1}	2.7761 × 10^{-1}	1.1362 × 10^{-1}	2.8841 × 10^{-1}	5.3260 × 10^{-2}	1.4534 × 10^{-2}	1.4941 × 10^{-2}	9.8127 × 10^{-1}	1.2113 × 10^{0}
	20	2.0359 × 10^{-1}	6.8854 × 10^{-1}	2.6697 × 10^{-1}	3.2814 × 10^{-1}	5.1906 × 10^{-1}	2.7346 × 10^{-1}	4.1281 × 10^{-1}	1.0349 × 10^{-1}	2.0318 × 10^{-2}	2.1762 × 10^{-2}	1.6273 × 10^{0}	2.0110 × 10^{0}
4	10	1.1177 × 10^{-1}	2.7257 × 10^{-1}	7.7811 × 10^{-2}	1.2206 × 10^{-1}	2.1258 × 10^{-1}	6.2052 × 10^{-2}	1.9197 × 10^{-1}	4.1152 × 10^{-2}	7.9316 × 10^{-3}	8.9709 × 10^{-3}	7.2936 × 10^{-1}	7.8920 × 10^{-1}
	20	2.2320 × 10^{-1}	6.1826 × 10^{-1}	1.9159 × 10^{-1}	2.6258 × 10^{-1}	5.4269 × 10^{-1}	2.5647 × 10^{-1}	7.8912 × 10^{-1}	1.9483 × 10^{-1}	4.1366 × 10^{-2}	3.4612 × 10^{-2}	2.8682 × 10^{0}	3.7093 × 10^{0}

5	10	5.3648×10^{-2}	1.4542×10^{-1}	3.8962×10^{-2}	7.4326×10^{-2}	1.5142×10^{-1}	5.2063×10^{-2}	2.4474×10^{-1}	8.0622×10^{-2}	1.3768×10^{-2}	1.1566×10^{-2}	7.1666×10^{-1}	7.4113×10^{-1}
	20	1.6525×10^{-1}	4.7654×10^{-1}	1.3915×10^{-1}	1.9834×10^{-1}	3.8818×10^{-1}	1.6009×10^{-1}	4.3545×10^{-1}	1.4531×10^{-1}	2.6064×10^{-2}	1.8355×10^{-2}	1.7283×10^{0}	1.4509×10^{0}
6	10	4.4056×10^{-2}	1.3806×10^{-1}	3.2750×10^{-2}	7.0892×10^{-2}	1.4872×10^{-1}	5.3626×10^{-2}	2.7163×10^{-1}	3.1263×10^{-2}	1.4474×10^{-2}	1.3685×10^{-2}	6.6748×10^{-1}	6.9069×10^{-1}
	20	1.8724×10^{-1}	5.9809×10^{-1}	1.5835×10^{-1}	2.4287×10^{-1}	5.0162×10^{-1}	1.7455×10^{-1}	5.0774×10^{-1}	1.6095×10^{-1}	3.6876×10^{-2}	2.2143×10^{-2}	2.3630×10^{0}	2.4782×10^{0}
7	10	7.3433×10^{-2}	1.6307×10^{-1}	6.5921×10^{-2}	9.9303×10^{-2}	1.6841×10^{-1}	7.9397×10^{-2}	2.4077×10^{-1}	8.4677×10^{-2}	1.6957×10^{-2}	9.0613×10^{-3}	6.9811×10^{-1}	7.0519×10^{-1}
	20	3.2614×10^{-1}	6.1695×10^{-1}	2.8400×10^{-1}	3.7577×10^{-1}	5.3031×10^{-1}	2.7886×10^{-1}	3.5505×10^{-1}	1.1893×10^{-1}	1.9912×10^{-2}	2.4909×10^{-2}	1.8082×10^{0}	1.9842×10^{0}
8	10	8.9202×10^{-2}	1.7738×10^{-1}	7.4339×10^{-2}	1.0917×10^{-1}	1.8742×10^{-1}	9.4297×10^{-2}	2.4965×10^{-1}	5.0080×10^{-2}	1.1885×10^{-2}	1.9738×10^{-2}	7.3456×10^{-1}	7.3379×10^{-1}
	20	5.1405×10^{-1}	8.9663×10^{-1}	5.0118×10^{-1}	5.4868×10^{-1}	8.4199×10^{-1}	5.8507×10^{-1}	1.2007×10^{0}	2.5572×10^{-1}	4.8640×10^{-2}	5.3545×10^{-2}	3.0841×10^{0}	4.4608×10^{0}
9	10	6.8645×10^{-2}	1.4986×10^{-1}	5.6682×10^{-2}	9.1781×10^{-2}	1.7700×10^{-1}	7.6954×10^{-2}	1.9704×10^{-1}	4.0701×10^{-2}	1.0682×10^{-2}	1.1539×10^{-2}	6.7000×10^{-1}	7.1762×10^{-1}
	20	4.2335×10^{-1}	8.1851×10^{-1}	3.9586×10^{-1}	4.6441×10^{-1}	7.3528×10^{-1}	4.6651×10^{-1}	7.2185×10^{-1}	1.7936×10^{-1}	4.0610×10^{-2}	4.2834×10^{-2}	3.0822×10^{0}	4.0222×10^{0}
10	10	7.2881×10^{-2}	1.5586×10^{-1}	5.2480×10^{-2}	8.9332×10^{-2}	1.7179×10^{-1}	6.9320×10^{-2}	2.4323×10^{-1}	3.6997×10^{-2}	1.3821×10^{-2}	1.1672×10^{-2}	6.2544×10^{-1}	6.9988×10^{-1}
	20	3.1135×10^{-1}	6.9010×10^{-1}	2.9058×10^{-1}	3.5189×10^{-1}	6.1874×10^{-1}	2.9727×10^{-1}	6.0760×10^{-1}	2.0661×10^{-1}	4.5630×10^{-2}	4.0018×10^{-2}	2.5657×10^{0}	2.6710×10^{0}
11	10	1.5134×10^{-1}	2.8824×10^{-1}	1.3514×10^{-1}	1.7398×10^{-1}	2.8224×10^{-1}	1.1852×10^{-1}	2.2513×10^{-1}	3.6543×10^{-2}	2.1207×10^{-2}	1.1110×10^{-2}	1.2609×10^{0}	1.3731×10^{0}
	20	1.9804×10^{-1}	4.4457×10^{-1}	1.8519×10^{-1}	2.2910×10^{-1}	3.3310×10^{-1}	1.9293×10^{-1}	2.3402×10^{-1}	7.9082×10^{-2}	1.8811×10^{-2}	1.6590×10^{-2}	9.7187×10^{-1}	1.2922×10^{0}
12	10	8.6517×10^{-2}	1.6311×10^{-1}	7.5754×10^{-2}	1.1114×10^{-1}	1.8995×10^{-1}	9.1003×10^{-2}	1.8339×10^{-1}	2.1826×10^{-2}	8.4467×10^{-3}	1.0654×10^{-2}	7.8459×10^{-1}	9.3326×10^{-1}

20	2.5937 $\times 10^{-1}$	5.2313 $\times 10^{-1}$	2.3267 $\times 10^{-1}$	1.8895 $\times 10^{-1}$	2.6068 $\times 10^{-1}$	1.6576 $\times 10^{-1}$	2.4155 $\times 10^{-1}$	4.9822 $\times 10^{-2}$	1.5827 $\times 10^{-2}$	1.2497 $\times 10^{-2}$	7.6327 $\times 10^{-1}$	1.0323 $\times 10^{0}$
10D Mean Time	8.1890 $\times 10^{-2}$	1.8382 $\times 10^{-1}$	6.5705 $\times 10^{-2}$	1.0254 $\times 10^{-1}$	1.8823 $\times 10^{-1}$	7.5201 $\times 10^{-2}$	2.2532 $\times 10^{-1}$	4.9704 $\times 10^{-2}$	1.2445 $\times 10^{-2}$	1.1409 $\times 10^{-2}$	7.6090 $\times 10^{-1}$	8.8029 $\times 10^{-1}$
10D Rank	6	8	4	7	9	5	10	3	2	**1**	11	12
20D Mean Time	2.5886 $\times 10^{-1}$	6.1132 $\times 10^{-1}$	2.3910 $\times 10^{-1}$	2.9478 $\times 10^{-1}$	4.9789 $\times 10^{-1}$	2.5803 $\times 10^{-1}$	4.9952 $\times 10^{-1}$	1.4166 $\times 10^{-1}$	2.8711 $\times 10^{-2}$	2.6087 $\times 10^{-2}$	1.9387 $\times 10^{0}$	2.2665 $\times 10^{0}$
20D Rank	6	10	4	7	8	5	9	3	2	**1**	11	12

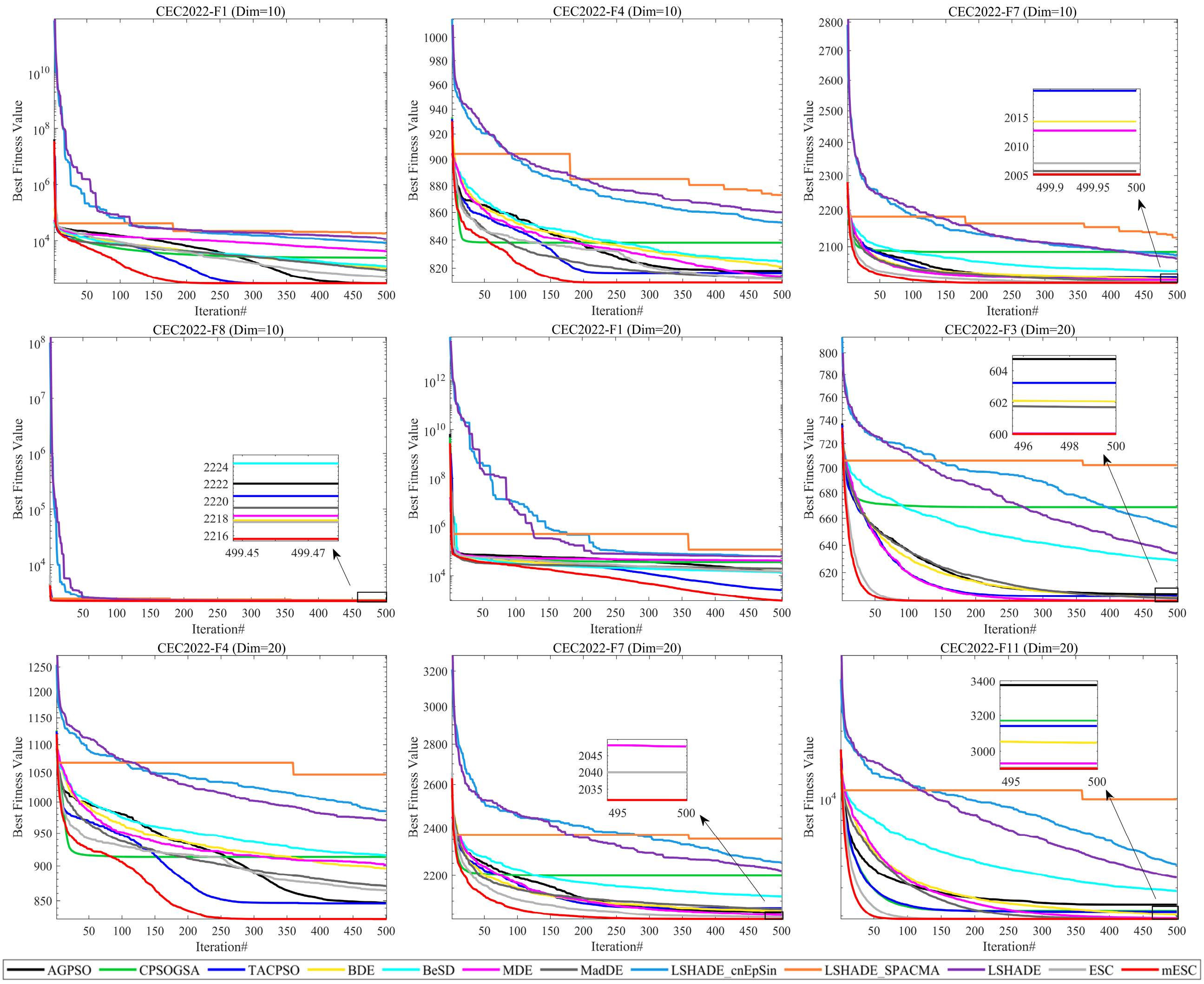

Figure S3. Convergence curves of mESC and high-performance, winner algorithm on CEC2022.

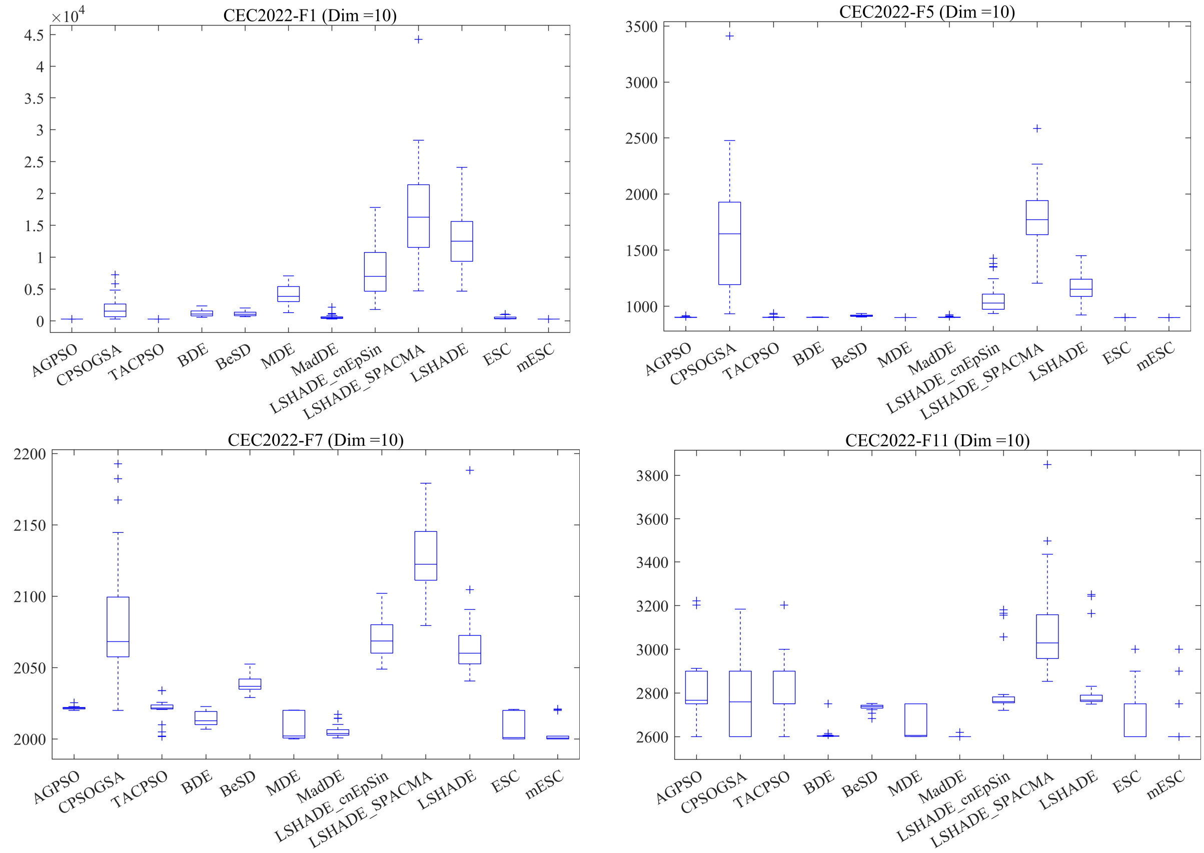

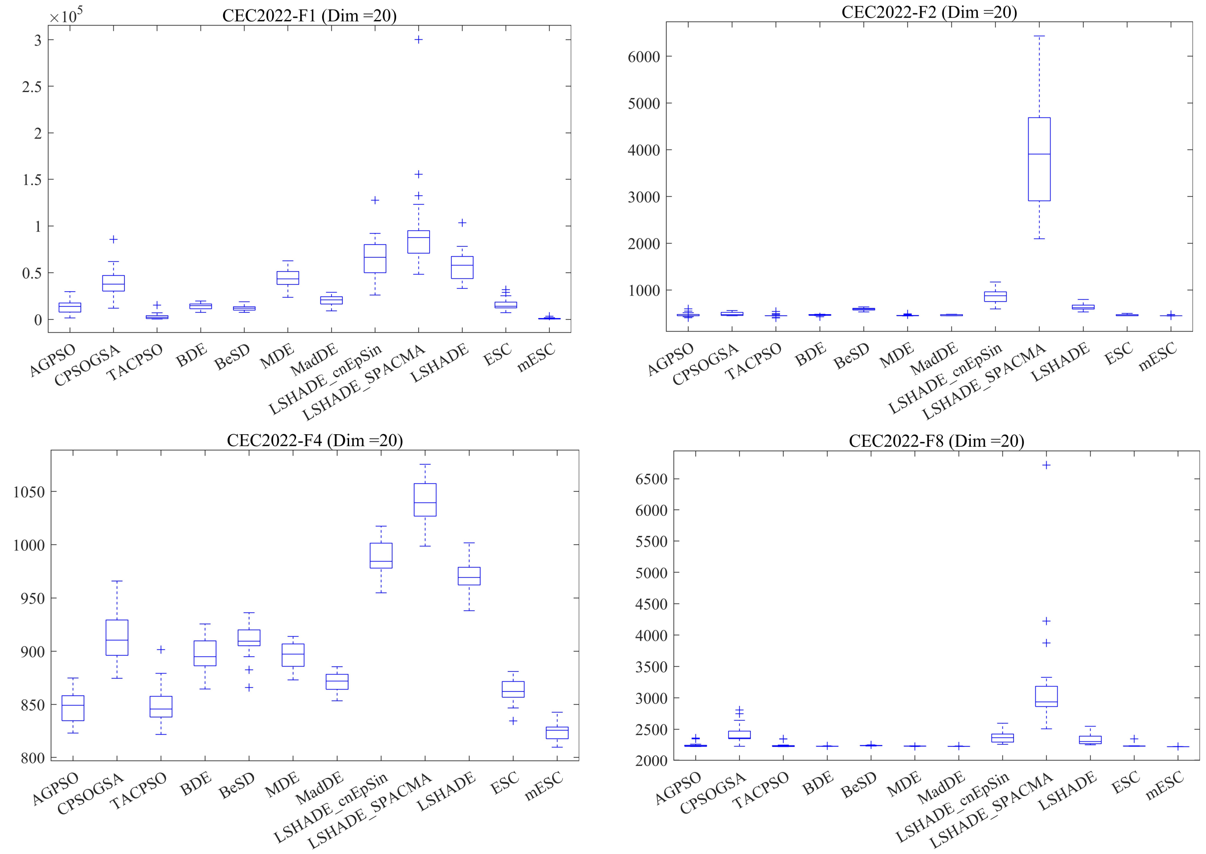

Figure S4. Box plot of mESC and high-performance, winner algorithm on CEC2022.
